# Identification of Histone Lysine Acetoacetylation as a Dynamic Post‐Translational Modification Regulated by HBO1

**DOI:** 10.1002/advs.202300032

**Published:** 2023-06-29

**Authors:** Yan Gao, Xinlei Sheng, Doudou Tan, SunJoo Kim, Soyoung Choi, Sanjita Paudel, Taeho Lee, Cong Yan, Minjia Tan, Kyu Min Kim, Sam Seok Cho, Sung Hwan Ki, He Huang, Yingming Zhao, Sangkyu Lee

**Affiliations:** ^1^ College of Pharmacy Kyungpook National University Daegu 41566 Republic of Korea; ^2^ Ben May Department for Cancer Research The University of Chicago Chicago IL 60637 USA; ^3^ Shanghai Institute of Materia Medica Chinese Academy of Sciences Shanghai 201203 China; ^4^ College of Pharmacy Chosun University Gwangju 61452 South Korea; ^5^ University of Chinese Academy of Sciences Beijing 100049 China; ^6^ School of Pharmacy Sungkyunkwan University Suwon 16419 South Korea; ^7^ Department of Biomedical Science, College of Natural Science Chosun University Gwangju 61452 South Korea

**Keywords:** acetoacetate, acetoacetylation, epigenetics, HBO1, histone marks, novel histone modification

## Abstract

Ketone bodies have long been known as a group of lipid‐derived alternative energy sources during glucose shortages. Nevertheless, the molecular mechanisms underlying their non‐metabolic functions remain largely elusive. This study identified acetoacetate as the precursor for lysine acetoacetylation (Kacac), a previously uncharacterized and evolutionarily conserved histone post‐translational modification. This protein modification is comprehensively validated using chemical and biochemical approaches, including HPLC co‐elution and MS/MS analysis using synthetic peptides, Western blot, and isotopic labeling. Histone Kacac can be dynamically regulated by acetoacetate concentration, possibly via acetoacetyl‐CoA. Biochemical studies show that HBO1, traditionally known as an acetyltransferase, can also serve as an acetoacetyltransferase. In addition, 33 Kacac sites are identified on mammalian histones, depicting the landscape of histone Kacac marks across species and organs. In summary, this study thus discovers a physiologically relevant and enzymatically regulated histone mark that sheds light on the non‐metabolic functions of ketone bodies.

## Graphical abstract

Lysine acetoacetylation (Kacac) is a histone post‐translational modification derived from acetoacetate. It is a conserved and uncharacterized modification that has been confirmed through chemical and biochemical methods. Acetoacetate concentration, possibly mediated by acetoacetyl‐CoA, can regulate this modification dynamically. HBO1, previously recognized as an acetyltransferase, can also function as an acetoacetyl‐transferase. This study has identified 33 Kacac sites on histones in mammalian cells and organs.

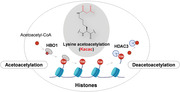

## 1 Introduction

The discovery and characterization of intracellular metabolites and their affiliated metabolic pathways are central to cellular metabolism. Despite the expanding knowledge of cellular metabolites and their roles in metabolism, the non‐metabolic functions of metabolites are relatively understudied. Recently, a multitude of metabolites have been reported as precursors and cofactors for protein post‐translational modifications (PTMs), which regulate cellular functions.^[^ ^1^, ^2^ ^]^ For example, acetate and succinate are precursors for lysine acetylation (Kac) and lysine succinylation (Ksu) via acetyl‐CoA and succinyl‐CoA, respectively, while S‐adenosyl methionine is the cofactor for lysine methylation.^[^ ^3^, ^4^ ^]^ The formation of such modifications can be through either enzymatic or spontaneous chemical processes,^[^ ^1^, ^3^, ^5^ ^]^ indicating a link between cellular metabolism and protein modifications. Recently, a group of lysine acylations have been identified, such as lysine 2‐hydroxyisobutyrylation (Khib) and crotonylation (KCr), which utilize short‐lipid metabolites as precursors and their corresponding CoAs as cofactors.^[^ ^1^ ^]^ These acylations are dynamically regulated by two groups of enzymes, acyl‐transferases, and deacylases, which catalyze the addition and removal of the modifications, respectively.^[^ ^6^ ^]^

**Figure 1:**
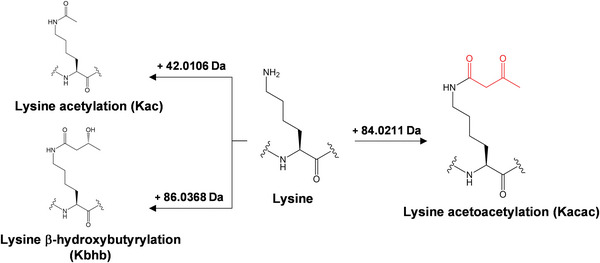
Proteomic approaches for identifying lysine acetoacetylation (Kacac). An illustration of lysine acetoacetylation and its predicted mass shift of +84.0211 Da. Lysine residues can be modified by acylation, such as Kac (+42.0106 Da), and Kacac (+84.0211 Da).

Growing evidence suggests that short‐chain lysine acylations are closely associated with diverse pathophysiological conditions and are involved in disease progression.^[^ ^7^, ^8^, ^9^ ^]^ One such example is lysine *β*‐hydroxylbutyrylation (Kbhb) fueled by *β*‐hydroxylbutyrate (BHB). BHB, acetoacetate, and acetone collectively form ketone bodies. These ketone molecules are generated from fatty acids in the liver via ketogenesis, when glucose is not readily available. The levels of ketone bodies are markedly elevated under extreme physiological changes (such as starvation) or pathological conditions (such as diabetes).^[^ ^10^ ^]^ For many years, these molecules have been known as alternative energy sources for peripheral tissues, such as the heart and brain. However, this long‐standing paradigm was challenged by the discovery that BHB functions as a precursor for histone Kbhb, likely via *β*‐hydroxylbutyryl‐CoA.^[^ ^11^ ^]^ The Kbhb levels can be enzymatically regulated by previously known acetyltransferases and deacetylases.^[^ ^11^ ^]^ In addition, histone Kbhb marks directly stimulate gene expression as assessed by in vitro assays and are associated with active expression of starvation‐related genes. This pattern of regulation by Kbhb marks is different from that of the widely studied histone acetylation marks.^[^ ^11^ ^]^ By performing in vitro biochemical assays, we demonstrated that p300 can catalyze the addition of Kbhb using *β*‐hydroxylbutyryl‐CoA, while both histone deacetylase1 (HDAC1) and HDAC2 can enzymatically remove Kbhb. The discovery of Kbhb and its regulatory machinery prompted us to hypothesize that acetoacetate, another ketone molecule, and a short‐chain fatty acid, can also serve as a precursor for its corresponding PTM, lysine acetoacetylation (Kacac) (**Figure** **1**).

Herein, we report the identification of a novel lysine modification, Kacac, induced by acetoacetate. We utilized diverse biochemical assays to validate the presence and prevalence of Kacac on histones. In total, we detected 33 Kacac sites on mammalian histones. Some of the identified sites are conserved across distinct species, implying evolutionarily conserved functions. Furthermore, this modification can be dynamically regulated by treating cells with ethyl acetoacetate (EAA) and an antagonist of the ketogenesis pathway. Using ex vivo and in vitro assays, we identified HBO1 as a histone acetoacetyltransferase. Thus, our work uncovers a previously uncharacterized PTM pathway that connects ketone bodies to histone epigenetic regulation.

## 2 Results

### 2.1 Identification of Lysine Acetoacetylation on Histone Proteins

Previous studies suggest that short‐chain fatty acids can serve as precursors for lysine acylations.^[^ ^1^, ^6^ ^]^ For example, acetate‐derived Kac, is a consequence of acetyl‐CoA ligation by p300/CBP;^[^ ^3^ ^]^ BHB, derived from ketogenesis, can stimulate histone Kbhb via BHB‐CoA^[^ ^11^ ^]^ (Figure 1). Considering these observations, we hypothesized that acetoacetate acts as a precursor for Kacac in a manner similar to acetate for Kac and BHB for Kbhb.

A PTM is typically identified by mass spectrometry, via measurement of the mass shift at the modified amino acid residues in a peptide sequence. As an example, acetylation induces a mass shift of +42.0106 Da at the modified Lys residues. Based on this principle, the chemical formula of Kacac indicates a mass shift of +84.0211 Da (monoisotopic mass, C_4_H_4_O_2_) in the modified peptides (Figure 1). To hunt for Kacac peptides, we designed a proteomics workflow: core histones were extracted from cultured human cells and rat livers, which were proteolytically digested by trypsin, with or without in vitro lysine propionylation. The resulting tryptic peptides were analyzed by HPLC‐MS/MS followed by protein sequence alignment to identify peptides with a mass shift of +84.0211 Da at Lys residues. This approach allowed us to identify several histone peptides bearing this mass shift, which are presumably caused by Kacac, across species. It is noteworthy that these peptides were detected without the conventional enrichment using modification‐specific antibodies, which suggests appreciable abundances of acetoacetylated peptides.

### 2.2 Validation of Kacac by HPLC Co‐Elution and MS/MS Using Synthetic Peptides

**Figure 2:**
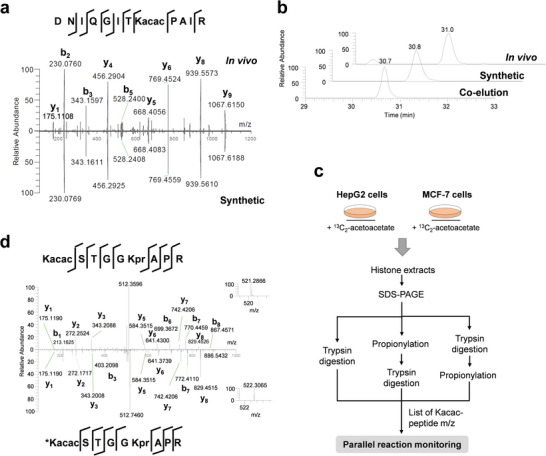
Verification of lysine acetoacetylation (Kacac) using synthetic peptides and isotopic labeling. a) MS/MS spectra of H4K31 Kacac peptides (DNIQGITKacacPAIR) derived from MCF7 cells (upper) and its synthetic counterpart (lower). b) Extracted ion chromatograms of the endogenous H4K31 peptide, synthetic H4K31 peptide, and a mixture of both. c) Experimental workflow for analyzing isotopically labeled Kacac histone peptides. d) MS^2^ spectra of light (upper) and heavy (lower) Kacac peptide (H3K23) KacacQLATKprAAR. The ^13^C_2_‐Kacac peptide, Kacac*QLATKprAAR, was identified in HepG2 cells treated with 10 mm [2,4‐^13^C_2_] acetoacetate and validated by PRM. Inset: MS^1^ spectra of the precursor ion.

Using our proteomics approach and utilizing human HepG2 and MCF7 cell lines, we identified 30 peptides bearing the predicted mass shift at Lys residues, including H3K9 (K_+84.02_STGGKprAPR), H3K18 (K_+84.02_QLATKprAAR), and H4K31 (DNIQGITK_+84.02_PAIR) (Table S1, Supporting Information). Although the observed mass shift perfectly matched to the predicted shift by Kacac, further verification of this modification is still needed, given the possible existence of isomeric modifications. Therefore, we employed a series of biochemical assays to unambiguously demonstrate that the mass shift of +84.0211 Da indeed results from acetoacetylation at lysine residues. We therefore synthesized three representative peptides bearing Kacac: H3K9 (KacacSTGGKprAPR), H3K18 (KacacQLATKprAAR), and H4K31 (DNIQGITKacacPAIR). These synthetic peptides should possess identical chemical properties as that of the endogenous peptides, exhibiting similar fragmentation patterns, and retention times. Therefore, if one cell‐derived peptide and its corresponding synthetic peptide carry the same modification, they are expected to co‐elute in HPLC and display similar fragment patterns (MS/MS spectra).^[^ ^1^ ^]^ In agreement with our expectation, the synthetic peptide (H4K31, DNIQGITKacacPAIR) showed near identical fragmentation patterns with its endogenous counterpart (**Figure** **2**a). In addition, the two peptides co‐eluted in HPLC (Figure 2b). These two lines of evidence strongly suggest that the mass shift of +84.02 Da is indeed attributed to lysine acetoacetylation. Analysis of the other two synthetic peptides with their corresponding cell‐derived counterparts corroborated this conclusion (Figure S1, Supporting Information).

### 2.3 Acetoacetate is a Precursor for Kacac

Our earlier study reported that *β*‐hydroxybutyrate functions as a precursor for Kbhb.^[^ ^11^ ^]^ Given their structural and functional similarities, we hypothesized that acetoacetate acts as a precursor for Kacac, in a fashion similar to BHB for Kbhb. Therefore, we carried out an isotopic labeling experiment to testify this possibility. We dosed HepG2 and MCF7 cells with 10 mm ^13^C_2_‐ EAA for 24 h, followed by extraction and tryptic digestion of core histones. The resulting tryptic peptides from the cells were analyzed by parallel reaction monitoring (PRM)‐mass spectrometry to detect isotopically labeled Kacac sites (Figure 2c). This analysis allowed us to identify five histone Kacac sites bearing ^13^C_2_‐isotopically‐labeled acetoacetylation from HepG2 cells (Figure 2d; Table S2, Supporting Information). Likewise, we identified six isotopically‐labeled Kacac sites on core histones from MCF7 cells (Table S2, Supporting Information). Taken together, these results convincingly demonstrate that acetoacetate acts as a metabolic precursor for histone Kacac, analogous to other types of short‐chain fatty acids for histone lysine acylations.^[^ ^1^ ^]^

### 2.4 Pan Anti‐Kacac Antibody Verifies Histone Kacac

**Figure 3:**
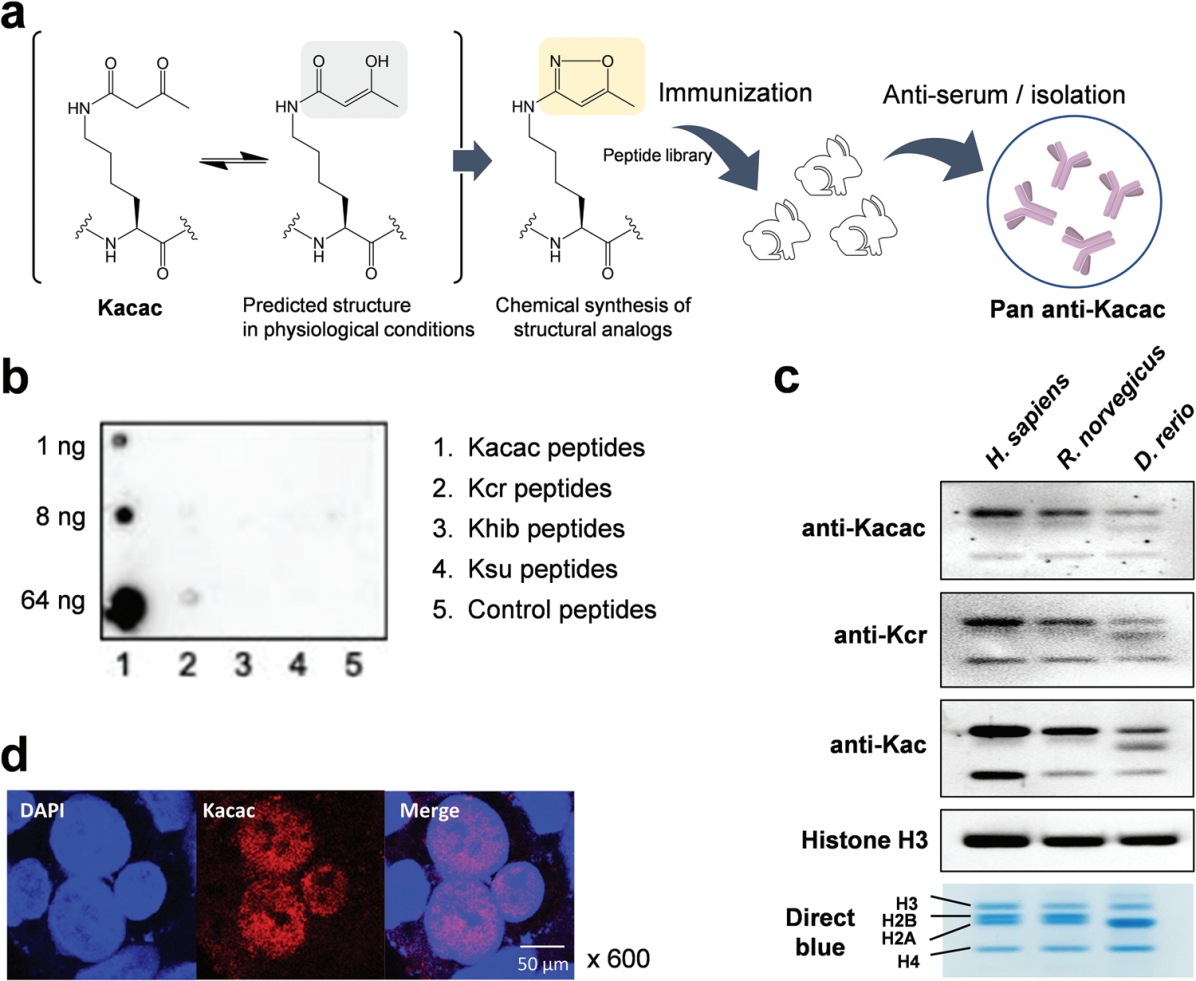
Generation and validation of pan anti‐Kacac antibody. a) Schematic strategy for generating pan anti‐Kacac antibody using structural analogs of Kacac. Peptides bearing methylisoxazole group that mimics acetoacetyl‐lysine were chemically synthesized and injected to rabbits as an antigen. The resulting antibody was extracted from the serum and isolated by affinity purification. b) Dot blot assay verifying the specificity of anti‐Kacac antibody. A dot‐blot assay was performed using the purified anti‐Kacac antibody against peptides bearing Kacac, Kcr, Khib, Ksu, and unmodified K, with 1, 8, and 64 ng of modified peptides blotted for each group. The other six replicates were shown in Figure S2b (Supporting Information). c) Western blot of histone Kacac, Kcr, and Kac in *Homo sapiens*, *Rattus norvegicus*, and *Danio rerio*. (d) Immunofluorescence images of Kacac in HepG2 cells. Blue: DAPI; red: Kacac.

To substantiate the validity of Kacac, we attempted to generate a pan antibody against Kacac. This would provide an orthogonal approach to verify Kacac and offer a valuable reagent for biochemical studies. We first used Kacac‐modified peptides as an immunogen but did not observe the modification‐specific immunogenic response in rabbits. We then turned to an alternative antigen using peptides bearing a Kacac structure mimic. Such an approach has been used to generate antibodies against histidine phosphorylation by Kee et al.^[^ ^12^ ^]^ We argue that under physiological conditions, a methylisoxazole group can be converted to a structural analog of acetoacetyl group (**Figure** **3**a). Therefore, peptides bearing methylisoxazole moiety on lysine residues were synthesized as alternative antigens to generate a pan‐anti‐Kacac antibody (Figure S2a, Supporting Information). The specificity of the generated anti‐Kacac antibodies was confirmed by dot‐blot assays using peptide libraries containing Kacac, Kcr, Khib, Ksu, and unmodified lysine (Figure 3b; Figure S2b, Supporting Information). No binding was observed for 2‐hydroxyisobutyrylated, succinylated, and unmodified peptides, and much lower binding was seen for crotonylated peptides. The high specificity of the antibody allowed for a clear distinction between Kacac and other modifications.

Using this newly generated pan‐anti‐Kacac antibody, we detected histone Kacac in diverse animal species, including *Homo sapiens*, *Rattus norvegicus*, and *Danio rerio* (Figure 3c). Given that ketone bodies are a group of evolutionarily conserved metabolites, it is highly likely that Kacac, similar to acetylation and crotonylation, is prevalent in diverse animals. This result confers additional confidence in the validity of Kacac. Moreover, this antibody also opens doors to various biochemical analyses, such as immunofluorescence and immunoprecipitation. Next, we applied this pan‐anti‐Kacac antibody to HepG2 cells and observed that Kacac signal is highly enriched in the nucleus (Figure 3d), suggesting that histone proteins are likely the hotspot of Kacac and at least part of the regulatory elements of Kacac may exist in the nucleus. This antibody also allowed us to detect several Kacac peptides from the core histones of human HepG2 and MCF7 cells and rat livers (Table S1, Supporting Information). In summary, we validated Kacac by four orthogonal methodologies, HPLC co‐elution, MS/MS, isotopic labeling, and Western blotting using anti‐Kacac antibody.

### 2.5 Histone Kacac is Dynamically Regulated by EAA and Ketogenesis Pathway

Short‐chain acyl‐CoAs can be generated from their corresponding short‐chain lipids. For example, acetyl‐CoA and BHB‐CoA can be generated from acetate and BHB, which consequently serve as precursors for Kac and Kbhb, respectively. Thus, we proposed that histone Kacac can be regulated by intracellular acetoacetate levels and ketogenesis pathway.

**Figure 4:**
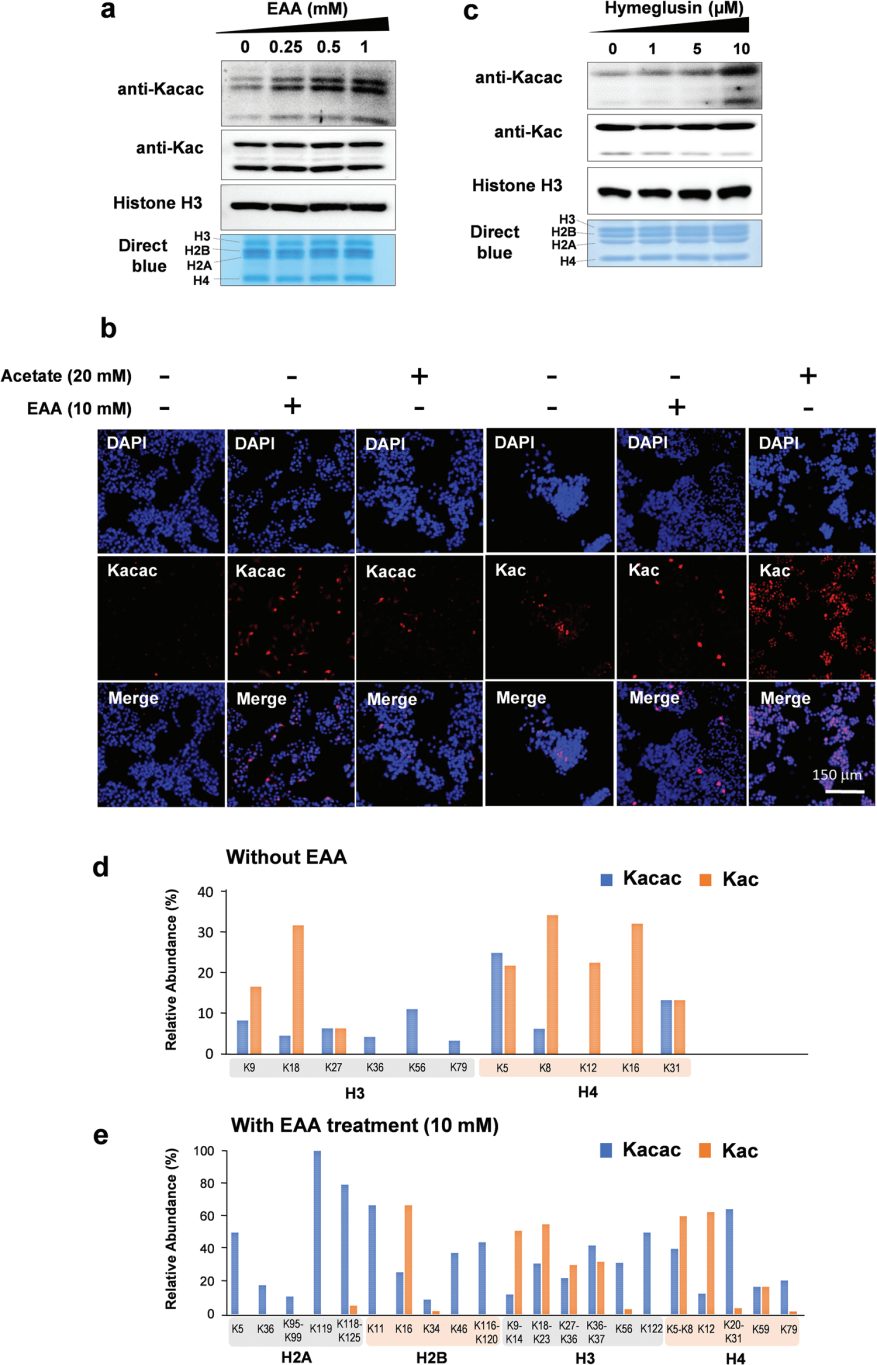
Dynamic regulation of Kacac by acetoacetate and acetoacetyl‐CoA. a) Western blots of Kac and Kacac on core histones extracted from HepG2 cells in response to ethyl acetoacetate (EAA). HepG2 cells were cultured with 0, 0.25, 0.5, and 1 mm of EAA for 24 h before histone extraction. Histones were extracted and subjected to Western blot analysis. b) Immunofluorescence microscopy images of cells treated with acetate (20 mm) or EAA (10 mm). Blue: DAPI; red: Kac or Kacac as indicated. c) Western blots of histone Kac and Kacac from HepG2 cells after treatment with hymeglusin. HepG2 cells were treated with hymeglusin at the concentrations of 0, 1, 5, and 10 mm for 24 h before harvesting, and the histones were extracted for Western blot analysis. d,e) Relative occupancies of Kacac and Kac sites in HepG2 cells under normal conditions (d) or EAA treatment (e). Histones from HepG2 cells were prepared after vehicle or EAA treatment for 24 h, and they were subsequently analyzed by HPLC‐MS/MS to quantify the spectral counts of Kacac peptides. Relative occupancy was calculated by dividing the spectral counts of each Kacac site by the total spectral counts of the site‐containing peptides.

To test this hypothesis, we treated HepG2 cells with 0–1 mm EAA for 24 h. In agreement with our expectation, we observed a concentration‐dependent increase of histone Kacac levels in response to EAA 24 h after treatment (**Figure** **4**a). The increase can be observed as early as 1 h after treatment (data not shown). In comparison, only a slight increase of Kpr levels was observed, with no noticeable change for other histone acylations, Kac, Kcr, and Kbu (Figure 4a; Figure S3a, Supporting Information). It is not surprising that Kbhb was slightly upregulated, considering the conversion from acetoacetate to *β*‐hydroxybutyrate. To substantiate this claim, we treated HepG2 cells with acetate or EAA and performed immunofluorescence microscopy to determine their effect on Kacac and Kac levels. Indeed, EAA markedly elevated the fluorescent signal of Kacac without inducing Kac (Figure 4b). Similar to Kac, heterogeneity was observed in Kacac levels, wherein some cells exhibited stronger signals, which probably resulted from differential uptake of EAA or the variable abundances of the regulatory factors. This result suggests that Kacac and Kac, although both localized to the nucleus, are regulated by distinct mechanisms.

Next, we asked whether Kacac levels are linked to ketogenesis. We exposed cells to various concentrations of hymeglusin, a specific *β*‐lactone inhibitor of eukaryotic hydroxymethylglutaryl‐CoA synthase, an enzyme catalyzing the conversion from acetoacetyl‐CoA to HMG‐CoA. This inhibitor is expected to increase intracellular acetoacetyl‐CoA and decrease HMG‐CoA levels. Indeed, histone Kacac levels increased dramatically after treatment with hymeglusin, which indirectly suggested that acetoacetyl‐CoA acts as a precursor for Kacac (**Figure** 4c). However, this increase in Kacac is not as drastic as acetoacetate treatment probably due to slower flux and accumulation of acetoacetyl‐CoA after hymeglusin treatment. Additionally, the hymeglusin treatment also led to a slight decrease in Kpr and increase in Kcr levels, probably due to the indirect effect of hymeglusin via acetyl‐CoA and the TCA cycle (Figure S3b, Supporting Information). These results suggest that histone Kacac is dynamically regulated by intracellular acetoacetate and acetoacetyl‐CoA levels.

The discrepancy in Kacac and Kac levels upon EAA treatment led us to propose that different regulatory mechanisms may underlie these two modifications. We speculate that some of the histone lysine residues may be more responsive to EAA, which may mediate downstream signaling pathways. Therefore, characterizing the dynamics of histone Kacac in a site‐specific fashion is critical for understanding the function of Kacac. Thus, we used an enrichment‐free HPLC‐MS/MS method to compare the relative abundance of Kacac with Kac. Spectral counts of peptides bearing Kac and Kacac were quantified in HepG2 cells with or without EAA treatment (10 mm). Relative occupancy of a modification site is calculated by dividing the spectra counts (cut‐off: Mascot score >20, expect <2) of peptides containing Kac or Kacac modification by the total spectral counts of the peptides containing the target peptide sequence. In the non‐EAA‐treated samples, both Kacac and Kac were found on six lysine sites, i.e., H3K9, H3K18, H3K27, H4K5, H4K8, and H4K31. Three histone H3 sites, H3K36, H3K56, and H3K79, were only modified by Kacac but not Kac (**Figure** 4d). Overall, the occupancies of Kacac sites were slightly lower than those of Kac sites without the EAA treatment. However, Kacac levels were markedly upregulated in EAA‐treated cells. More Kacac sites were detected, such as H2AK5, H2AK36, H2AK199, H2BK46, H3K122, and H4K79. The overall occupancies of Kacac were higher than those of Kac in the majority of the sites (**Figure** 4e). This line of evidence underscores the possibility that Kacac levels on histones are dynamically upregulated in response to acetoacetate level and ketogenesis. Given the high occupancy and dynamics of Kacac, this modification may have a global impact on the functions of histone proteins, probably regulating transcription of starvation‐related genes, similar to that observed for Kbhb.

### 2.6 HBO1 is a Lysine Acetoacetyltransferase

**Figure 5:**
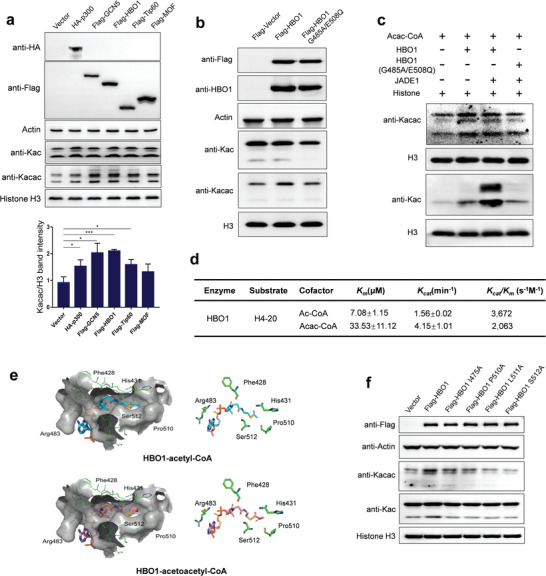
Validation of the acetoacetyl‐transferase activity of HBO1. a) Validating the acetoacetyltransferase activity of histone acetyltransferases (HATs) in cells. Empty vector, HA‐p300, Flag‐GCN5, Flag‐Tip60, and Flag‐MOF were transfected individually into 293T cells, and core histones were extracted from the cells that overexpressed HATs. The levels of Kac and Kacac were determined using Western blot analysis and quantitatively analyzed. Three replicates were conducted to perform the statistical analysis. *p < 0.05, **p < 0.01, ***p < 0.005. b) Histone Kacac levels upon overexpression of HBO1 WT and catalytically‐dead mutant (G485A/E508Q). c) In vitro acetoacetyltransferase assay of HBO1. Recombinant proteins, HBO1 WT, HBO1 mutant, and JADE1 were expressed and purified. The recombinant proteins were incubated with extracted histone proteins and substrate, acac‐CoA or ac‐CoA, and the level of Kacac and Kac were assessed by Western blot analysis. d) Kinetics data of HBO1 toward Kacac and Kac. In the assay, H4‐20 peptide and HBO1 were co‐incubated with varying concentrations of either acetyl‐CoA or acetoacetyl‐CoA for 30 min at 30 °C. The resulting fluorescent CoAS‐CPM complex was measured as an indicator of enzymatic activity. The kinetic constants, Km and Kcat, were determined using the Michaelis–Menten model. All data are represented as means ± SEM (n = 3). e) Predicted binding modes of acetyl‐coenzyme A (CoA) and acetoacetyl‐CoA to HBO1. Acetyl‐CoA is shown in cartoon representation in cyan, acetoacetyl‐CoA in gray pink and their interacting amino acid residues are labeled. f) The histone Kacac levels in response to the overexpression of WT and mutant (Pro510A, Leu511A, Ile475A, and Ser512A) HBO1.

The dynamics and prevalence of Kacac prompted us to pursue its underlying regulatory mechanisms. Increasing evidence indicates that the classical acetyltransferases can also regulate a variety of newly discovered acyl modifications. For instance, p300 is known as a promiscuous acyl‐transferase that can catalyze the transfer of various acyl moieties to lysine residues, including acetyl, lactyl, succinyl, and methacryl groups.^[^ ^13^ ^]^ Considering these observations, we hypothesized that some of the acetyltransferases could function as acetoacetyltransferases. To test this hypothesis, we transfected HEK293T cells with gene constructs that encode five conventional acetyltransferases individually, namely p300, GCN5, HBO1, Tip60, and MOF (**Figure** **5**a). Overexpression (OE) of these enzymes upregulated Kac levels. Meanwhile, they all elevated histone Kacac levels to varying degrees, among that GCN5 and HBO1 OE led to the most pronounced increase. The fold changes of Kacac upon GCN5 and HBO1 OE are higher than those of Kac, corroborating the dynamics of this modification. To identify the sites regulated by GCN5 and HBO1, histone peptides from these acetyltransferase‐OE cells were analyzed by PRM‐mass spectrometry analyses. The occupancies for two Kacac sites, H2AK5 and H4K12, increased in GCN5 OE cells, while H2BK34, H4K12, and H4K79 occupancies showed more than two‐fold increase with HBO1 OE. Additionally, H2AK5 and H2BK16 were only detected in HBO1 OE cells and not mock‐treated cells (Table S3, Supporting Information). These results demonstrate HBO1 is an acetoacetyltransferase that regulates various histone Kacac sites.

To substantiate the enzymatic activity of HBO1, we overexpressed the wild‐type and a catalytically dead mutant (G485A/E508Q) of HBO1 in HEK293T cells. The level of histone Kacac was enhanced upon overexpression of the wild‐type HBO1, whereas this increase was abrogated by the mutant HBO1 (**Figure** 5b). To further corroborate the ex vivo result, we carried out an in vitro assay using recombinant proteins. The wild‐type HBO1 catalyzed the Kacac reaction; however, the catalytically dead mutant almost did not exhibit this activity (**Figure** 5c). JADE1 is a scaffolding protein known to promote HBO1‐mediated acetylation reaction. As a control, Kac level was increased by HBO1 and further elevated by JADE1; nevertheless, the HBO1 mutant abolished this increase. However, the addition of JADE1 did not result in a further increase in Kacac, suggesting that its acetoacetyltransferase and acetyl‐transferase activities can be distinctly regulated. To further investigate the enzymatic activities of HBO1, we conducted a kinetic assay based on a previously published method.^[^ ^14^ ^]^ In this assay, we utilized an in vitro fluorometric acylation assay that employs acetoacetyl‐CoA as the cofactor and synthetic human histone H4 peptides as the substrate. Using this assay, we quantitatively compared the acyl‐transferase activities of HBO1 in the Kac and Kacac reactions. HBO1 exhibited Kcat/Km of 2063 s^−1^ m ^−1^ for Kacac, which accounted for approximately 56% of its Kcat/Km for Kac (**Figure** 5d). Collectively, these results demonstrate that HBO1 catalyzes Kacac both in vitro and in cells, and JADE1 does not have a synergetic effect on the acetoacetyltransferase activity of HBO1 in vitro.

To better understand the molecular mechanism underlying the acetoacetyl‐transferase activity of HBO1, we performed 3D in silico molecular modeling of acetoacetyl‐CoA bound to HBO1 using AutoDock based on its binding to acetyl‐CoA (**Figure** 5e). The CoA binding pocket in HBO1 allows proper fitting of acetoacetyl‐CoA. Furthermore, the acetoacetyl moiety is positioned at the bottom of the pocket, forming a hydrophobic interaction with Pro510 and Leu511 residues (Figure S4, Supporting Information). Similar to acetyl‐CoA, the carbonyl group adjacent to the sulfur atom forms a hydrogen bond with the nitrogen atom of Ile475. In addition, the *β*‐carbonyl group of the acetoacetyl moiety forms an additional hydrogen bond with the nitrogen atom of Ser512 on the backbone, stabilizing the binding of acetoacetyl‐CoA. To verify the crucial amino acid residues involved in the interaction between HBO1 and acetoacetyl‐CoA, we conducted an overexpression experiment using both the wild‐type (WT) and multiple mutant variants of HBO1 (Pro510A, Leu511A, Ile475A, and Ser512A) in HEK293T cells. The overexpression of WT HBO1 led to an increase in histone Kacac levels. However, this effect was abolished in cells expressing the mutant HBO1 variants (**Figure** 5f). These findings provide compelling evidence that HBO1 binds to acetoacetyl‐CoA and exerts its catalytic activity through the predicted key amino acid residues.

### 2.7 Identification of HDAC3 as a Lysine Deacetoacetylase

**Figure 6:**
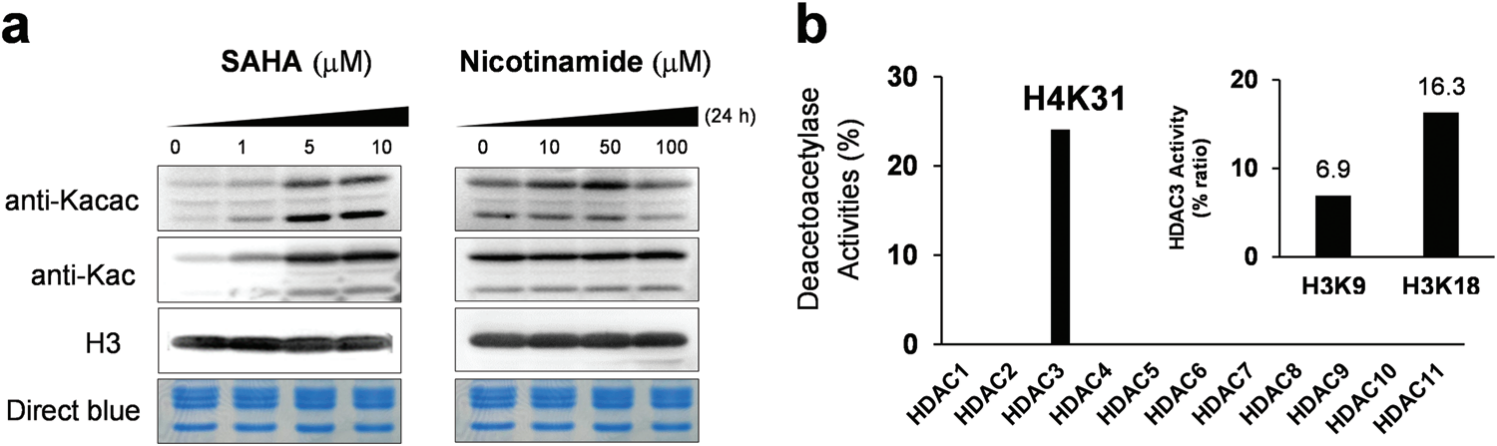
Histone deacetoacetylation (Kacac) catalyzed by HDAC3. a) Immunoblot of histone Kacac levels on core histones after SAHA or nicotinamide treatment for 24 h. b) In vitro deacetoacetylation assay by LC‐MS/MS. The synthetic peptides bearing Kacac on H3K9, H3K18, and H4K31 were incubated with individual HDACs (HDAC classes 1 and 2, HDAC1‐11), and the enzyme activities were determined as the peak area ratios of the peptide ions (unmodified peptide/total acetoacetylated peptide).

Another essential aspect of the regulation of PTM_S_ is their removal. Histone modifications are known to be erased by histone deacetylases (HDACs) family,^[^ ^15^ ^]^ including sirtuins (SIRTs). For example, sirtuin 5 (SIRT5) carries out desuccinylation and demalonylation by removing succinyl and malonyl moieties from target lysine residues.^[^ ^16^ ^]^ Additionally, a recent screen of HDACs revealed that HDAC3 catalyzes the removal of lysine methacrylation in vitro.^[^ ^17^ ^]^ Here, we presume that Kacac might be regulated by the HDACs family.^[^ ^18^ ^]^ To test our hypothesis, we treated HepG2 cells with an HDAC inhibitor, suberoylanilide hydroxamic acid (SAHA), or a SIRTs inhibitor, nicotinamide, and histone Kacac levels were examined by immunoblotting 24 h post‐treatment. After SAHA treatment, histone Kacac levels, in agreement with Kac levels, increased concentration‐dependently, whereas no significant change occurred after nicotinamide treatment (**Figure** **6**a).

To further determine which that specific HDAC enzyme carries out deacetoacetylase activity, we performed in vitro screening of individual HDACs 1–11 using synthetic Kacac peptides, H3K9 (KacacSTGGKprAPR), H3K18 (KacacQLATKprAAR), and H4K31 (DNIQGITKacacPAIRR). By quantifying the ratios of unmodified peptides via LC‐MS/MS, we discovered that only HDAC3 displayed noticeable deacetoacetylation activity, where 6.9%, 16.3%, and 24.1% of the modified peptides were deacetoacetylated for H3K9, H3K18, and H4K31, respectively (**Figure** 6b). This result demonstrated that HDAC3 can catalytically remove histone acetoacetylation in vitro.

### 2.8 Mapping Histone Kacac Marks

**Figure 7:**
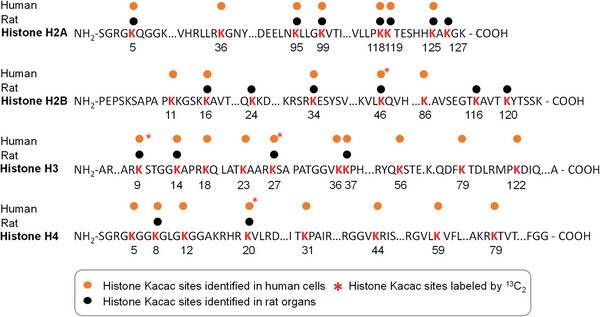
Kacac map on human and rat core histones. Schematic sequences of the four core histone proteins, with Kacac sites color‐coded by their datasets in this study. Yellow dots indicate Kacac sites detected in human HepG2 cells; black dots mark Kacac sites from rat organs. * denotes sites induced by ^13^C_2_‐ethyl acetoacetate treatment (10 mm) in HepG2 cells.

To further expand the catalog of Kacac sites, we analyzed Kacac in additional rat organs, including the liver, kidney, lung, and spleen. Here, histone proteins were extracted, typically digested, and subjected to our HPLC‐MS/MS analysis. Our analysis identified 11, 13, 10, and 14 Kacac sites on core histones from the liver, kidney, lung, and spleen samples, respectively (Figure S5 and Table S4, Supporting Information). To acquire a more comprehensive understanding of Kacac sites, we integrated the identified Kacac sites from rat organs with those detected in human HepG2 and MCF7 cells and marked them on the core histones (**Figure** **7**). In total, 33 Kacac sites were identified, and a majority of these sites were detected in both human and rat. The spectra of all the identified Kacac peptides were manually inspected and representative MS^2^ spectra for each of the 33 Kacac sites are shown in Figure S6 (Supporting Information). Next, we summarized all the Kacac sites identified in our study and aligned them with the other common histone lysine short‐chain acylations, including succinylation, lactylation, crotonylation, and acetylation. Many of these Kacac sites overlap with previously identified lysine acylations. (Figure S7, Supporting Information). We also applied the abovementioned approach to identify Kacac sites in human, mouse, and fruit fly cells. The identified Kacac sites were indicated on a consensus Histone H3 and H4 sequence. Overall, we detected 10, 5, and 7 Kacac sites from human MCF7 cells, mouse MEF cells, and *Drosophila* S2 cells, respectively (Figure S8, Supporting Information). In particular, Kacac sites on H3K9, H3K18, H3K36, and H4K8 were detected across all the three species, and half of the other Kacac sites were detected in more than one species.

## 3 Discussion

Recent studies show that the short‐chain lysine acylations on histone proteins are regulated by intracellular short‐chain fatty acids and their corresponding acyl‐CoAs.^[^ ^1^ ^]^ These histone PTMs modulate chromatin structure and gene expression.^[^ ^1^ ^]^ They are closely associated with different diseases, such as cancer and metabolic diseases, and physiological changes (such as fasting).^[^ ^19^, ^20^ ^]^ Therefore, a metabolite‐regulated PTM offers a potential feedback mechanism to couple cellular metabolism with gene expression.

Ketone bodies are a group of metabolites that provide energy to diverse organs in low‐glucose conditions. During ketogenesis, acetoacetate is converted to BHB in hepatic mitochondria; conversely, BHB can also be turned to acetoacetate, resulting in the accumulation of acetyl‐CoA in extrahepatic mitochondria.^[^ ^21^ ^]^ Although the metabolic roles of these ketone body molecules have been recognized, their non‐metabolic functions remain to be discovered. Emerging lines of evidence suggest diverse functions of ketone bodies in addition to metabolism^[^ ^22^ ^]^; however, the molecular mechanisms underlying their functions are not known. Identification of histone Kacac marks thus uncovers an additional avenue through which cells can sense starvation and induce downstream signaling pathways to maintain homeostasis by regulating histone modifications.

Growing evidence demonstrates that lysine acylations are distributed through various organelles. We started to focus on Kacac sites on histone proteins specifically, as they potentially have profound impact on transcription. Histone lysine modifications, with the exception of Kac and Kme, such as crotonylation, succinylation, and malonylation, are not always detectable without immune‐affinity enrichment due to low stoichiometry.^[^ ^23^ ^]^ In contrast, our non‐enrichment workflow could identify dozens of Kacac sites, and our preliminary results indicate that a number of Kacac sites may exist at high stoichiometry. The relative occupancies of histone Kacac sites are on par with Kac under normal culture conditions. Moreover, the levels of Kacac were upregulated by EAA treatment independent of Kac. This implies that Kacac is a dynamic modification responsive to starvation and disruption of ketogenesis pathway. In addition, the discrepancy in Kacac and Kac trends is indicative of a distinctive regulatory mechanism. This high stoichiometry of Kacac may confer the capability to regulate chromatin functions in relevant pathological and physiological conditions. Further, many Kacac sites on H2A and H2B were highly upregulated upon the EAA treatment, whereas they were not detected in the control group. The mechanism underlying this discrimination is not understood. Further investigation is necessary to understand the mechanism by which these sites are involved in regulating cellular responses to EAA.

Functional characterization of a novel histone modification demands an understanding of its regulatory components, particularly enzymatic writers and erasers that add and remove the modifications, respectively. For example, p300 was reported to act as a Kbhb transferase, and HDAC1 and HDAC2 were reported to remove Kbhb.^[^ ^24^ ^]^ In this study, we uncovered the acetoacetyl‐transferase activity of HBO1, a multifunctional MYST acetyltransferase specific for histone H3 and H4 and a positive regulator of DNA replication.^[^ ^25^, ^26^ ^]^ In addition to HBO1, other acetyltransferases also contribute to Kacac levels, and different Kacac sites were observed in GCN5 and HBO1 OE cells, implying crosstalk between acetoacetyl‐transferases in a site‐specific manner. Possibly, those Kacac sites may respond differently to various environmental cues, under the regulation of distinct acetoacetyl‐transferases. In agreement with the results from the EAA treatment, overexpression of these enzymes also led to a more pronounced increase in Kacac levels than in Kac levels. This finding further corroborates that Kacac is governed by a different subset of regulatory elements than those of Kac. We also analyzed the mechanisms underlying the removal of Kacac and reported HDAC3 as a histone lysine deacetoacetylase. However, our assay was performed with only a small subset of peptides in vitro. Thus, whether other HDACs could carry out deacetoacetylase activity toward a different subset of substrates under more physiologically relevant conditions remains to be studied.

Furthermore, Kacac level was found to be stimulated by elevated acetoacetyl‐CoA concentration, as revealed by hymeglusin treatment. Acetoacetyl‐CoA is produced from the ketogenesis pathway via condensation of two acetyl‐CoA molecules or the breakdown of HMG‐CoA within the mitochondria. Alternatively, it is likely synthesized from acetoacetate by acetoacetyl‐CoA synthetase (AACS) in the cytosol. Short chain acyl‐CoA molecules are not known to directly transport across mitochondrial membranes, but the nuclear localization of Kacac revealed by immunofluorescence implies a nuclear pool of acetoacetyl‐CoA. It remains an open question as to if and how acetoacetyl‐CoA is available in the nucleus. One possibility is that a fraction of AACS is localized to the nucleus that synthesizes acetoacetyl‐CoA from acetoacetate. If this hypothesis is valid, it leads to a related question regarding the origin of nuclear acetoacetate. Furthermore, our study also raises other questions regarding the regulatory mechanisms and functions of Kacac. Specifically, what are the reader proteins of Kacac? What are the epigenetic functions of histone Kacac? Which non‐histone proteins can be modified by Kacac and what are the functional consequences?

## 4 Experimental Section

See the Supporting Information.

## Conflict of Interest

Y.Z. is a founder, board member, advisor to, and inventor on patents licensed to PTM Bio Inc. (Hangzhou, China and Chicago, IL, USA) and Maponos Therapeutics Inc. (Chicago, IL, USA). The other authors declare no competing interests.

## Supporting information

Supporting Information

Supporting Information

## Data Availability

The data that support the findings of this study are available from the corresponding author upon reasonable request.
