# Supplementary material for: Identification of Histone Lysine Acetoacetylation as a Dynamic Post‐Translational Modification Regulated by HBO1
Source: Adv Sci (Weinh). 2023 Jun 29;10(25):2300032. doi: 10.1002/advs.202300032 (PMC10477889; doi:10.1002/advs.202300032)
Supplement: Supplementary file 1 — Supporting Information [file ADVS-10-2300032-s002.pdf]

ADVANCED SCIENCE
Open Access

Supporting Information

for *Adv. Sci.*, DOI 10.1002/advs.202300032

Identification of Histone Lysine Acetoacetylation as a Dynamic Post-Translational Modification Regulated by HBO1

Yan Gao, Xinlei Sheng, Doudou Tan, SunJoo Kim, Soyoung Choi, Sanjita Paudel, Taeho Lee, Cong Yan, Minjia Tan, Kyu Min Kim, Sam Seok Cho, Sung Hwan Ki, He Huang, Yingming Zhao* and Sangkyu Lee**

Supporting Information

Identification of histone lysine acetoacetylation as a dynamic post-translational modification regulated by HBO1

Yan Gao[#], Xinlei Sheng[#], Doudou Tan[#], SunJoo Kim[#], Soyoung Choi, Sanjita Paudel, Taeho Lee, Cong Yan, Minjia Tan, Kyu Min Kim, Sam Seok Cho, Sung Hwan Ki, He Huang, Yingming Zhao* and Sangkyu Lee*

Supporting methods

Cell culture and histone extraction.

Cell culture - Human HepG2 cells, MCF7 cells, and mouse MEF cells, were grown in DMEM (Hyclone Laboratories Inc., Logan, UT, USA) supplemented with 10% (v/v) fetal bovine serum (FBS, Hyclone Laboratories Inc., Logan, UT, USA), 1% (v/v) GlutaMAX (Gibco, Thermo Fisher Scientific, Inc., CA, USA) and 1% (v/v) penicillin-streptomycin (Gibco, Thermo Fisher Scientific, Inc., CA, USA) at 37°C with 95% air and 5% CO_2. *Drosophila* S2 cells were grown in Schneider's medium (Gibco, Thermo Fisher Scientific, Inc., CA, USA) containing 10% (v/v) FBS at 26°C. To increase the intracellular level of Kacac, HepG2, MCF7, MEF, and S2 cells were treated with a concentration gradient of ethyl-acetoacetate (Sigma-Aldrich, St. Louis, MO, USA) for 24 hrs before harvesting.

Histone extraction - Extraction of core histones from HepG2, MCF7, MEF and S2 cells was carried out as previously described [1-4]. The culture medium was removed when cells reached 80% confluence and cells were washed with 1× Dulbecco's phosphate-buffered saline (DPBS, Gibco, Thermo Fisher Scientific, Inc., CA, USA). Cells were detached by 0.25% trypsin–EDTA (Gibco, Thermo Fisher Scientific, Inc., CA, USA) at 37°C for 3 mins. The cell suspension was centrifuged at 1,000 g for 3 min. Harvested cells were washed with ice-cold DPBS, resuspended in lysis buffer (10 mM HEPES pH 7.0, 10 mM KCl, 1.5 mM $MgCl_2$, 0.34 M sucrose, 0.5% NP40) with pipetting, and incubated on ice for 30 min with gentle stirring. The cell lysate was centrifuged at 2,000 g at 4°C for 10 min. After discarding the supernatant, the pellet was washed with washing buffer (10 mM HEPES pH 7.0, 10 mM KCl, 1.5 mM $MgCl_2$, 0.34 M sucrose), and then the histone proteins were extracted with 0.4 N H_2SO_4 at 4°C for 4 hrs. The suspension was centrifuged at 16,000 g for 20 min at 4°C, and the histone-containing supernatant was saved in a low protein binding tube for subsequent analyses.

Hymeglusin treatment - To decrease the intracellular level of acetoacetate, HepG2 cells were treated with hymeglusin (Sigma-Aldrich, St. Louis, MO, USA) at concentrations of 0, 1, 5, and 10 μM for 24 hrs before harvesting.

Treatment of $^{13}C_2$-ethyl acetoacetate - To verify whether acetoacetate is the precursor of histone Kacac, ethyl acetoacetate-1,3-$^{13}C_2$ (Sigma-Aldrich, St. Louis, MO, USA) was added to HepG2 and MCF7 cells. Cells were treated with 10 mM ethyl acetoacetate for 24 hrs before harvesting.

Animal experiments and histone extraction. For histone extraction from rat tissues, 5-week-old SD male rats were obtained from Charles River, Korea (Seoul, Korea) (IRB No 2021-0025). Upon arrival, the animals were randomized and housed as four animals per cage and acclimated for one week. The animal room was maintained at 23 ± 3°C and 50 ± 10% relative humidity. A 12-hr cycle of light and dark was used. All the animal procedures followed the guidelines recommended by the Society of Toxicology (USA) in 1989. After scarified under inhalation anesthetic, liver, kidney, lung, and spleen tissues were isolated. Tissue samples were homogenized using a glass Dounce homogenizer (20 strokes) in ice-cold lysis buffer. The homogenate was passed through two layers of cheesecloth and then centrifuged at 2,000 g at 4°C for 10 min. The supernatant was discarded, and cell pellet was briefly washed with wash buffer. Histones were extracted from cell pellet using 0.4 N H_2SO_4 at 4°C for 4 hrs and subsequently processed as described above. All animal use and experiments were conducted according to Y.Z.'s approved animal protocol (ACUP#72296) at the University of Chicago.

For histone precipitation, trichloroacetic acid was added to the histone-containing supernatants to a final concentration of 30% (v/v) and precipitated on ice for 12 hrs. The suspensions were centrifuged at 2,000 g at 4°C for 10 mins. Precipitated histone pellets were washed with ice-cold acetone and dried by SpeedVac (Labconco Co, Kansas City, MO, USA). Histone proteins were dissolved in distilled water and quantified using the BCA assay (Thermo Fisher Scientific, Inc., CA, USA). Extracted histone proteins were stored in a -80°C freezer, until further analysis.

Co-elution of Kacac peptides using synthetic peptides. The synthetic peptides used in this study (H3K9acac, m/z 523.83, KacacSTGGKprAPR; H3K18acac, m/z 563.83 KacacQLATKprAAR; H4K31acac, m/z 705.39, DNIQGITKacacPAIR) were purchased from AnaSpec, Inc. (Fremont, CA, USA). The lysine acetoacetylated peptide from tryptic digests of

human MCF7 histones, its synthetic counterpart, and their mixture were injected into a nano-HPLC system and analyzed by a Q-Exactive mass spectrometer (Thermo Fisher Scientific, San Jose, CA) . The MS/MS spectra and retention times of the cell-derived and synthetic peptides were compared.

Synthesis of HG191001. The structural analog of Kacac (HG191001) was synthesized based on the commercially available Cbz-Lys-OMe strategy (**Figure S2a**). The synthesis starts with the preparation of iodide by diazonium formation ($NaNO_2$, H_2SO_4) and substitution (I_2) [5]. The Cbz-protected isoxazole derivative was introduced by reaction with 3-amino-5-methylisozole [6]. The resulting HG191001 was obtained using sequential reactions of 2 as described in Liu *et al.,* 2008 [7].

Development of Kacac-specific antibodies. A peptide library containing the synthesized structural analogs of Kacac was prepared as an antigen to generate anti-Kacac antibodies by PTM Biolabs Inc. (Hangzhou, Zhejiang, China) [8, 9]. For immunization, antigen solution containing a library of Kacac analog peptides (XXXXX-Kacac-XXXXX, X represents a random amino acid residue) was mixed with complete Freund's adjuvant (Sigma-Aldrich, St. Louis, MO, USA) at a ratio of 1:1(v/v) and injected to five New Zealand rabbits. After 9 weeks of immunization, blood was collected from carotid arteries. The collected blood sample was incubated at 37°C for 2 hrs and then transferred to 4°C for overnight incubation. Blood sample was centrifuged at 6,000 *g* at 4°C for 20 min, and the antiserum-containing supernatant was collected. The generated antibody was purified using an affinity column pre-activated with 20 mL of phosphate-buffered saline (PBS). Antiserum was loaded to the activated affinity column, and the pass-through serum was reloaded. Column was washed with PBS until $OD_{280nm} < 0.05$. The purified antibody was eluted with 0.2 M glycine (pH 2.8) and neutralized with 1 M Tris-buffered saline, pH 8.0.

Dot-blot assay. Antigen was prepared at a final concentration of 10-50 μg/mL in TBST buffer (Tris-buffered saline, pH 7.4, 0.5% (v/v) Tween 20). Polyvinylidene fluoride (PVDF) membrane was gridded to position antigen spotting. Membrane was activated with methanol, washed by TBST, and spotted with the following antigens: Kacac peptides, crotonyllysine (Kcr) peptides, 2-hydroxyisobutyryllysine (Khib) peptides, succinyllysine (Ksu) peptides, and bovine serum albumin (BSA) peptides with an amount of 1, 8 and 64 ng. The modified peptide

libraries used in this assay are composed of randomized peptides with a sequence of XXXXX-K(modified)-XXXXX, where X represents a random amino acid residue. Membrane was air-dried and incubated with blocking buffer (5% (v/v) non-fat milk in TBST) for 30 mins. Membrane was washed in TBST buffer for 10 mins for 3 times. The primary antibody was diluted to 1:2000 in blocking buffer and incubated with membrane for 1 hr at room temperature (RT). Membrane was then washed in TBST buffer for 10 min for 3 times, and the secondary antibody was applied for 30 mins at RT. Membrane was washed in TBST buffer for 10 mins for 3 times. For blot detection, membrane was treated with HRP luminol substance for around 30 sec to 60 sec and assessed by using a Lumino image analyzer (LAS-4000, Fujifilm, Lexington, MA, USA).

Sample preparation for proteomic analysis.

In-gel digestion with chemical propionylation – Twenty micrograms of each histone sample from cells or animal tissue was resolved by 15% SDS-PAGE and visualized by Coomassie protein stain (Abcam, Cambridge, MA, USA). Histone bands were excised, decolorized with 50% ethanol, and dehydrated with acetonitrile (ACN). Samples were subjected to in-gel digestion with or without chemical derivatization. For chemical derivatization, dehydrated gel bands were hydrated in 100 mM ammonium bicarbonate (ABC) buffer. A volume of 25 μL of propionylation reagent (propionic anhydride: ACN at a ratio of 1:3 (v/v)) was added and ammonium hydroxide (NH_4OH) was added immediately to re-establish pH to 8.0 [10]. The reaction was kept at 37°C for 1 hr. Propionylated samples were digested with trypsin (Promega, Madison, WI, USA) at a ratio of 50:1 (w/w) at 37°C for 12 hrs. In a parallel experiment, histone proteins were subjected to in-gel digestion without derivatization, and the resulting peptides were extracted in 75% ACN/0.1% trifluoroacetic acid (TFA). Peptide extracts were dried in a SpeedVac without heating and re-constituted in 100 mM ABC buffer. The resuspended peptides were propionylated as described above.

Global immunoprecipitation of Kacac - Dried histone samples were dissolved in 100 mM ABC bufferat a protein concentration of 1 mg/mL, pH 8.0. For each 100 μL histone solution, 25 μL of propionylation reagent was added and NH_4OH was added immediately to re-establish pH to 8.0. Propionylated histones were digested with trypsin (Promega, Madison, WI, USA) at a ratio of 50:1 (w/w) at 37°C for 12 hrs. In a separate workflow, histone proteins were subjected to trypsin digestion followed by propionylation derivatization as described above. Propionylated histone peptides were desalted using the sep-pak cartridges (Waters, Milford, MA, USA) and

dried in SpeedVac. Acetoacetylated peptides were enriched by agarose beads conjugated with the generated Kacac-specific antibody (PTM Biolabs, Hangzhou, CN). Desalted histone peptides were dissolved in NETN buffer (2 mM EDTA, 0.04 M Tris-HCl, 0.2 M NaCl, and 1% NP40, pH 8.0). The peptide solution was centrifuged at 16,000 g for 10 mins at 4°C to remove possible precipitates. Pan-anti-Kacac antibody-conjugated beads were mixed with peptide solution and incubated at 4°C with gentle rotation for 12 hrs. To wash away the unbound peptides, peptides mixture was centrifuged at 1,000 g at 4°C for 1 min, and the supernatant was removed carefully. The remaining beads were washed with ice-cold NETN buffers 3 times and deionized water twice. The enriched Kacac peptides were eluted with 50 μL 0.15% (v/v) TFA for 3 times. The eluates were combined and dried in a SpeedVac without heating. Dried peptides were stored in a -80°C freezer.

LC-MS/MS analysis. Before MS analysis, dried peptide samples were desalted using Zip-Tip (5 μg) (Millipore, Milford, MA, USA). Desalted peptides were dissolved in 20 μL solvent A (2% ACN, 0.1% formic acid). Two microliter peptide sample was injected into a nano-LC (Eksigent, Dublin, CA, USA) integrated with an auto-sampler. A 60-min gradient nano-LC was employed to separate the peptides, and the flow rate was set to 300 nL/min. LC gradient started from 3% solvent B (0.1% formic acid in ACN) and ended with 28% solvent B. The nano-LC was integrated with an LTQ Orbitrap Velos (Thermo Fisher Scientific, San Jose, CA) at Mass Spectrometry Convergence Research Center. MS was run in positive mode. For data-dependent scan, precursor ions were scanned from m/z 300-1,500 m/z with a resolution of 60,000, with the lock mass-enabled (m/z at 445.12). The automatic gain control (AGC) target was 1.0×10^6 for the MS scan. For MS/MS scan, the top 10 most abundant precursor ions with an intensity more than 1.0×10^5 were fragmented in high-energy collisional dissociation mode with a resolution of 7,500 with 28% normalized collision energy and isolation width of 2.0 m/z.

Peak alignment. The raw MS data were searched in Mascot (version 2.3.0, Matrix Science Ltd, London, UK) against a human or rat or mouse database for protein and peptide identification. Lysine acetoacetylation, lysine acetylation (Kac), lysine propionylation (Kpr), methionine oxidation, protein *N*-terminal acetylation and lysine methylation were specified as variable modifications, while cysteine carbamidomethylation was set as the stable modification. Mass tolerance was set to 10 ppm for precursor ions and 0.05 Da for fragment ions. To filter out low-quality PTM identifications, we further discounted all peptides with a Mascot score <

20, significance threshold $p > 0.05$, and all peptides with C-terminal lysine modifications (unless the modified peptides are at the C-terminus of proteins). All spectra of Kacac-modified peptides were manually inspected to ensure high-quality peptide identification. The mass spectrometry proteomics data have been deposited to ProteomeXchange Consortium via the PRIDE partner repository with the dataset identifier PXD025392 [11].

Quantification of Kacac peptides by PRM. For the relative quantification of Kacac peptides, PRM was conducted. Target m/z values of Kacac peptides were acquired from our DDA scans (as listed in **Table S1**). Target m/z values of heavy-labeled Kacac sequences were calculated by replacing $^{12}C_2$ with $^{13}C_2$ in the identified Kacac sequences. For the full scan, precursor ions were scanned from 300 to 1,500 m/z with a resolution of 60,000 and the lock mass-enabled (m/z at 445.12). The AGC target was set to 1.0×10^6. For MS/MS, resolution was set to 7,500 with 35% normalized collision energy and 1.0 m/z isolation window. MS was run on positive mode. Fold changes of Kacac peptides were measured by the ratios of peak areas.

Stoichiometry of histone Kacac. Histones were extracted from MCF7 and HepG2 cells cultured with and without acetoacetate treatment. Histone peptides were prepared by in-gel digestion after chemical propionylation and analyzed by MS/MS using data-dependent scanning. The PTM stoichiometry (%) reported in this study is represented by the ratio of the number of MS/MS spectra for peptides bearing Kacac or Kac divided by the total number of MS/MS spectra for the same peptide sequence with any modifications (cut-off: Mascot score > 20, expect < 2).

Western blot. Ten micrograms of histone proteins were mixed with 4× SDS buffer (200 mM Tris-Cl, pH 6.8, 400 mM dithiothreitol (DTT), 8% SDS, 0.4% bromophenol blue, 40% glycerol) and denatured by heating at 95°C for 3 min. Denatured sample was cooled to the RT and loaded onto a 15% SDS-PAGE gel. SDS-PAGE was run at 120 V for 90 min. The proteins were then electro-transferred to a PVDF membrane (Roche, Basel, CH) by wet transfer at a voltage of 90 V for 120 min on ice. After transfer, the membrane was incubated with 5% (v/v) BSA (GenDEPOT, Barker, TX, USA) in TBST at RT for 2 hrs.

The membrane was incubated with the indicated primary antibody: anti-Kacac, anti-Kac, anti-Kpr, anti-Kcr, anti-Kbhb, anti-Kbu (PTM Biolabs, Hangzhou, CN), and anti-histone H3 (Cell Signaling Technology, Beverly, MA, USA). Primary antibodies were diluted at 1:1000

(v/v) in 5% (v/v) BSA and applied to membrane at 4°C overnight. Membrane was washed in TBST buffer for 10 min with gentle shaking for 3 times, and then incubated with the matching secondary antibody: horseradish peroxidase (HRP)-conjugated anti-mouse (#7076, Cell Signaling Technology, Danvers, MA, USA) or anti-rabbit antibody (#7074, Cell Signaling Technology, Danvers, MA, USA) with a dilution of 1:5000 (v/v) in TBST at RT for 90 min. Membrane was washed by TBST buffer 3 times. For visualization, membrane was dampened in 1 mL ECL reagent (GE Healthcare, Chicago, IL, USA) and analyzed by Image Quant LAS 4000 mini (GE Healthcare, Chicago, IL, USA) and iBright 1500 (Thermo Fisher Scientific, Inc., CA, USA).

Immunoaffinity purification of HBO1 and JADE1 from HEK293T cells. Plasmids encoding wild-type or mutant FLAG-HBO1 were individually transfected into HEK293T cells with or without the plasmid encoding FLAG-JADE1. Cells were collected 48 hrs after transfection. Then, cells were washed with ice-cold PBS and lysed with lysis buffer (50 mM Tris-HCl pH 7.5, 150 mM NaCl, Triton X-100 1%, 1 mM EDTA, 1 mM DTT, 8% glycerol, and protease inhibitors) on ice for 30 mins. After centrifugation at 13,000 g and 4°C for 10 mins, the supernatant was collected and incubated with 10 μL of FLAG-M2 beads at 4 °C for 2 hrs. After incubation, the supernatant was discarded and the FLAG-M2 beads were washed 3 times with wash buffer (20 mM Tris-HCl pH 7.5, 150 mM NaCl, 0.1% Triton X-100, 1 mM EDTA, 1 mM DTT, 8% glycerol, and protease inhibitor). The target protein was eluted by elution buffer (20 mM Tris-HCl pH 7.5, 150 mM NaCl, 0.1% NP40, 1 mM DTT, 10% glycerinum, and protease inhibitors).

HBO1 and GCN5 plasmids construction and overexpression. GCN5 coding sequence (NM_021078.3) was inserted into the vector pCMV-Flag between EcoRI and NotI sites. Similarly, HBO1 coding sequence (NM_007067.5) was inserted between NheI and NotI sites. These plasmids were transfected into HEK293T cells using Liposomal (YEASEN, Shanghai, China). Briefly, cells were seeded in a 6-well plate with 2×10^6 cells/well and ensured to have a 90% confluence at the time of transfection. Cells were transfected with 4 μg of vector plasmid or overexpression plasmid. After 48h, the transfected cells were harvested, and the proteins were extracted for Western blot analysis.

***In vitro* enzymatic assay for HBO1.** The synthetic H4-20 peptide, consisting of 20 amino acid

residues from the N-terminus of histone H4, was used as the acyl acceptors in the assay. The sequence of the H4-20 peptide is SGRGKGGKGLGKGGAKRHRGGK-biotin. The plasmid containing the full-length HBO1 gene was transfected into 293T cells for 48 hours, and the HBO1 protein was isolated using affinity purification. For the enzymatic assay, 200 mM of the H4-20 peptide and 100 nM of HBO1 were co-incubated with varying concentrations of either acetyl-CoA (Sigma-Aldrich, A2181) or acetoacetyl-CoA (Sigma-Aldrich, A1625) for 30 minutes at 30°C in a reaction buffer containing 50 mM HEPES pH 8.0 and 0.1 mM EDTA. To quench the enzymatic reaction and generate the fluorescent CoAS-CPM complex, 50 mM of 7-diethylamino-3-(40-maleimidylphenyl)-4-methylcoumarin (CPM; Sigma-Aldrich, 96669) in dimethyl sulfoxide (DMSO) solution was added. The fluorescence intensity of the CoAS-CPM complex was measured using a microplate reader (SpectraMax M5, Molecular Devices, Sunnyvale, CA, USA) with fixed excitation and emission wavelengths of 392 and 482 nm, respectively. Duplicate experiments were performed, and the kinetic constants Km and Kcat were determined using the Michaelis-Menten model.

Molecular modeling. Molecular modeling was conducted using AutoDock 4.2 [12]. Crystal structure coordinates of HBO1 were downloaded from the Protein Data Bank (PDB entry 5GK9, www.pdb.org). All solvent molecules were removed. Structures of HBO1 and Ac-CoA were extracted from 5GK9, and the structure of acetoacetyl-CoA was built based on the structure of acetyl-CoA using PyMol (v2.0, Schrödinger LLC). The crystal structure of HBO1 was used as the receptor, and the CoA binding pocket was defined using AutoGrid. The grid size was set to 42×46×42 points with a grid spacing of 0.375 Å. The grid box was centered on the ligand from the corresponding crystal structure complexes. The Lamarckian genetic algorithm was used for docking with the following settings: GA runs number of 100, maximum number of 25,000,000 energy evaluations, initial population of 150 randomly placed individuals, maximum number of 27,000 generations, mutation rate of 0.02, and crossover rate of 0.80. The conformation with the lowest predicted binding free energy of the most frequently occurring binding mode was selected. The interaction between ligand and protein was analyzed with LigPlot+ (v2.2) [13].

Histone acylation assay. Histones were extracted from HEK293T cells using a standard acid extraction protocol [1]. The reaction mixtures (containing 100 μM of acetoacetyl-CoA or acetyl-CoA, 2 μg of indicated enzymes, 100 nM TSA, and 4 μg of extracted histones) were

incubated in reaction buffer (25 mM Tris-HCl pH 8.0, 150 mM NaCl, 10% glycerol, 1 mM DTT) at 37°C for 1 hr. After incubation, 5× SDS loading buffer was added to the mixture to quench the reaction, and the levels of Kac and Kacac were determined by Western blot analysis.

HDACs enzyme screening for lysine deacetoacetylation. Three synthetic peptides with Kacac sites (H3K9aa; KacacSTGGKprAPR, H3K18aa; KacacQLATKprAAR, and H4K31aa; DNIQGITKacacPAIR) were incubated with 500 μM HDAC1-11 in 50 μL HDAC reaction buffer (130 mM NaCl, 3 mM KCl, 1 mM $MgCl_2$ in 25 mM Tris-HCl, pH 8.0) at 37°C for 30 min. After the reaction, the samples were dried using a SpeedVac (Thermo Fisher Scientific, Waltham, MA, USA), followed by desalting using C18 Zip-Tip (Merch Millipore, Burlington, MA, USA). Peptide samples were then dissolved in Solution A (0.1% Formic acid in 98% H_2O, 2% ACN) for analysis by mass spectrometry.

Table S1. List of all the identified Kacac sites on core histones of human HepG2 and MCF7 cells and rat liver

Histone	Sites	Modified sequnece	Mascot Score	Observed m/z	Charge	Expect	Identification		
							DDA	IP	Species
H2A	K5	G**Kacac**QGGKprAR	35 .8	471 .260	2+	0.09	O		R
		A**Kacac**QGGKprAR	37.9	478.271	2+	0.03		O	H
	K36	**Kacac**GNYSER	59.3	469.220	2+	0.00	O		H
	K95	NDEELN**Kacac**LLGK	23.7	678.854	2+	2.00	O		R, H
		NDEELN**Kacac**LLGKpr	24.7	706.867	2+	1.50	O		R
		NDEELN**Kacac**LLGRVTIAQGGVLPNIQAVLLPK	23.4	1099.637	3+	0.13		O	H
	K99	NDEELNKLLG**Kacac**	27.0	678.854	2+	1.00	O		R, H
		NDEELNKprLLG**Kacac**	63.8	706.874	2+	0.00		O	R, H
	K118	VTIAQGGVLPNIQAVLLP**Kacac**KTESHH**Kacac**	23.5	982.896	3+	0.42	O		R
		ATIAGGGVIPHIHKacSLIG**Kacac**Kac	34.5	722.433	3+	0.02		O	R, H
	K119	ATIAGGGVIPHIHKacSLIGKac**Kacac**	31.5	542.076	4+	0.04		O	R, H
		**Kacac**TESHHKpr	23.4	503.752	2+	1.10	O		R, H
		VTIAQGGVLPNIQAVLLPK**Kacac**TESHHKacac	23.7	737.425	4+	0.35	O		R
	K125	VTIAQGGVLPNIQAVLLPKacacKTESHH**Kacac**	23.5	982.896	3+	0.42	O		R
		VTIAQGGVLPNIQAVLLPK**Kacac**TESHH**Kacac**	23.7	737.425	4+	0.35	O		R
		VTIAQGGVLPNIQAVLLPKacKacTESHH**Kacac**	25.0	982.900	3+	0.24	O		R
		VTIAQGGVLPNIQAVLLPKmeKmeTESHH**Kacac**	34.1	723.418	4+	0.04		O	R
	K127	KprTESHHKV**Kacac**	24.1	617.334	2+	1.20		O	R, H
H2B	K11	SAPAP**Kacac**KGSK	27.8	527.798	2+	0.59	O		H
	K16	**Kacac**AVTKacAQK	54.2	500.296	2+	0.00	O		R, H

	K34	**Kacac**ESYSVYVYK	48.2	675.334	2+	0.00	O		R, H
		**Kacac**ESYSVYVYKpr	35.5	703.351	2+	0.11	O		R, H
	K46	VL**Kacac***QVHPDTGISSK	48.6	796.934	2+	0.01	O		R, H
	K86	LAHYS**Kacac**R	37.3	479.758	2+	0.09		O	H
	K116	HAVSEGT**Kacac**AVTKacacYTSSKpr	22.9	1009.519	2+	1.80	O		R
	K120	HAVSEGTKacacAVT**Kacac**YTSSKpr	22.9	1009.519	2+	1.80	O		R
	K9	**Kacac**STGGKacAPR	47.8	514.278	2+	0.01	O		R, H
		**Kacac***STGGKprAPR	51.0	521.285	2+	0.00	O	O	R, H, M,
		**Kacac**STGGKprAPRme	28.7	528.295	2+	0.35		O	R, H
	K14	KmeSTGG**Kacac***APR	33.4	500.277	2+	0.21	O		R, H
	K18	**Kacac**QLATKacAAR	33.4	556.825	2+	0.21	O		H, M
		**Kacac**QLATKprAAR	57.0	563.831	2+	0.00	O		H, M,
	K23	KacQLAT**Kacac**AARme	26.0	563.832	2+	1.10	O		R, H
		KprQLAT**Kacac**AARme	27.1	570.837	2+	0.86	O	O	R, H
**H3**	K27	**Kacac***SAPATGGVK	49.7	500.276	2+	0.01	O		R, H
		KacacSAPATGGVK(tri)K(tri)PHR	47.0	401.243	3+	0.05	O		H
		**Kacac**SAPATGGVKacKmePHRme	23.9	529.968	3+	1.90	O		H
		**Kacac**SAPATGGVKmeKprPHR	29.4	794.466	2+	0.29	O		H
		**Kacac**SAPATGGVKmeKprPHRme	29.6	801.459	2+	0.42	O		H
		**Kacac**SAPATGGVKprKPHR	59.9	525.309	3+	0.00	O		H
		**Kacac***SAPATGGVKprKprPHR	56.4	815.454	2+	0.00	O		R, H
		**Kacac**SAPATGGVKprKprPHRme	31.9	822.465	2+	0.30	O		H
		**Kacac**SAPATGGVKacacKacacPHR	28.3	843.465	2+	0.85	O		H
		**Kacac**SAPATGGVKacKacacPHR	72.1	822.463	2+	0.00	O		H

		**Kacac**SAPATGGVKKacacPHR	44.7	801.455	2+	0.02	O	H
	K36	KacacSAPATGGV**Kacac**KacacPHR	28.3	843.465	2+	0.85	O	H, M
		KacacSAPATGGV**Kacac**KacPHR	53	534.642	3+	0.015	O	H, M
	K37	KacacSAPATGGVKacac**Kacac**PHR	28.3	843.465	2+	0.85	O	R, H
		KacacSAPATGGVKac**Kacac**PHR	72.1	822.463	2+	0.00	O	R, H
		KacacSAPATGGVK**Kacac**PHR	44.7	801.455	2+	0.02	O	R
	K56	RmeYQ**Kacac**STELLIR	34.0	752.923	2+	0.18	O	H
		YQ**Kacac**STELLIR	31.6	667.869	2+	0.32	O	H
	K79	EIAQDF**Kacac**TDLR	38.0	710.359	2+	0.12	O	H
	K122	RVTIMP**Kacac**DIQLAR	30.7	812.963	2+	0.37	O	H
**H4**	K5	G**Kacac**GGKacGLGK	47.4	464.268	2+	0.01	O	H
		G**Kacac**GGKprGLGKpr	25.8	499.288	2+	0.81	O	H
		G**Kacac**GGKprGLGKprGGAKprR	72.0	761.938	2+	0.00	O	H, M
		G**Kacac**GGKprGLGKprGGAKacR	91.0	754.930	2+	0.00	O	H, M
		G**Kacac**GGKdiGLGKprGGAKprR	41.0	759.960	2+	0.13	O	H, M
	K8	GKprGG**Kacac**GLGKpr	25.5	499.286	2+	0.95	O	R, H
		GKprGG**Kacac**GLGKdiGGAKprR	39.0	759.960	2+	0.17	O	H, M
	K12	GLG**Kacac**GGAKacR	25.7	485.276	2+	0.95	O	H
	K20	**Kacac**VLRDNIQGITKPAIR	35.3	636.059	3+	0.02	O	R, H
		**Kacac***VLRDNIQGITKPprAIR	35.4	654.733	3+	0.02	O	H
		**Kacac***VLRmeDNIQGITKprPAIR	24.8	659.405	3+	0.14	O	H
	K31	DNIQGIT**Kacac**PAIR	50.0	705.390	2+	0.01	O	R, H
	K44	GGV**Kacac**RdiISGLIYEETR	38.0	597.330	2+	0.52	O	H
	K59	GVL**Kacac**VFLENVIR	46.0	735.938	2+	0.01	O	H

K79	**Kacac**TVTAMDVVYALK	52.0	761.914	2+	0.00	O	H
	**Kacac**TVTAMDVVYALKmeRme	54.8	853.965	2+	0.00	O	H
	**Kacac**TVTAMDVVYALKpr	34.7	789.935	2+	0.17	O	H
	**Kacac**TVTAMDVVYALKR	87.6	839.963	2+	0.00	O	H

*, isotopically labeled acetoacetyl-lysine. H, human HepG2 and MCF cells; R, rat liver; M, mouse MEF cells. DDA, data-dependent acquisition; IP, immunoprecipitation.

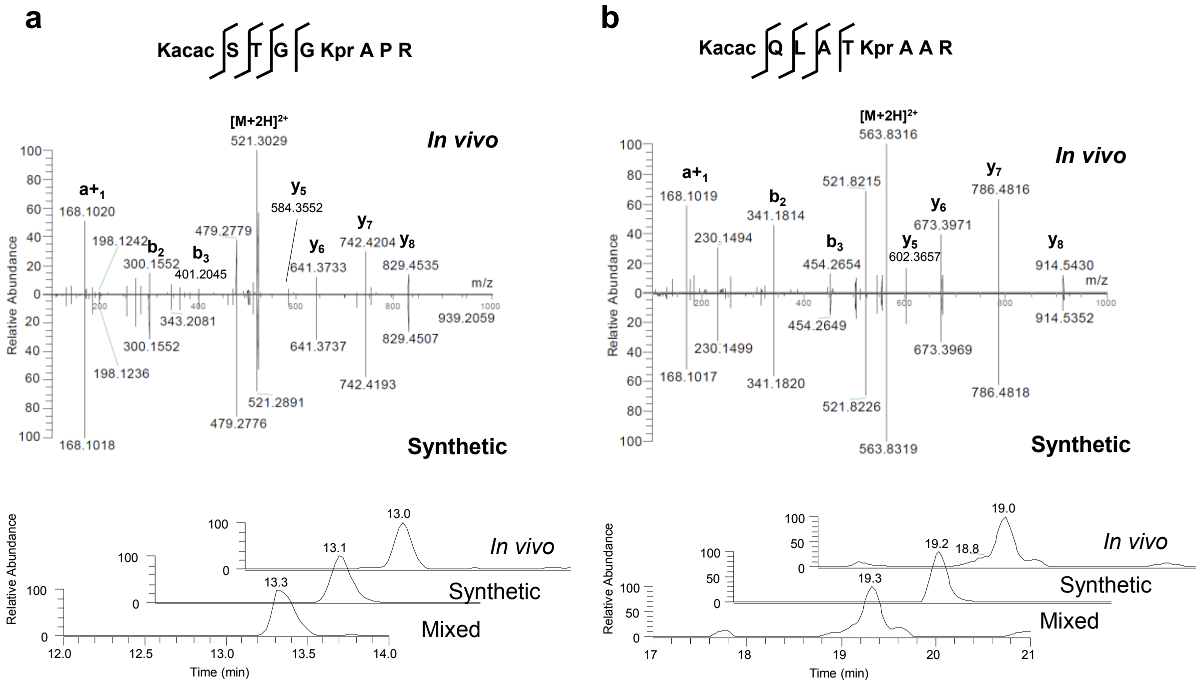

Figure S1. MS^2 spectra and chromatograms of Kacac peptides derived from MCF7 cells. MS/MS spectra (upper) and extracted ion chromatograms (lower) of H3K9 (KacacSTGGKprAPR) (**a**) and H3K18 (KacacQLATKprAAR) (**b**) peptides derived from MCF7 cells and its synthetic counterpart.

Table S2. A list of identified isotope-labeled $^{13}C_2$-Kacac sites on histones from human HepG2 and MCF7 cells

Sites	Detected sequences	Score	Observed m/z	Charge	Expect	Human cells	
						HepG2	MCF7
H2AZ2_K13	DSGKprA**Kacac***AK	26.1	473.762	2+	1.50	O	
H2AZ2_K15	AKprA**Kacac***AVSR	32.7	486.795	2+	0.29	O	
H3K9	**Kacac***STGGKprAPR	32.5	522.290	2+	0.06	O	
H3K14	KprSTGG**Kacac***APR	42.0	522.280	2+	0.12		O
H3K18	**Kacac***QLATKprAAR	44.0	564.830	2+	0.10		O
H3K23	KprQLAT**Kacac***AAR	45.0	5640830	2+	0.17		O
H3K27	**Kacac***SAPATGGVKprKprPHR	35.1	544.646	3+	0.00	O	
	**Kacac***SAPATGGVK	33.5	501.290	2+	0.08	O	
H4K5	G**Kacac***GGKacGLGKprGGAKprR	37.0	755.920	2+	0.82		O
H4K12	GKprGGKacGLG**Kacac***GGAKprR	38.0	755.920	2+	0.63		O
H4K16	GKprGGKprGLGKprGGA**Kacac***R	46.0	762.930	2+	0.12		O
H4K20	**Kacac***VLRDNIQGITKprPAIR	22.8	660.067	3+	0.02	O	
	**Kacac***VLRmeDNIQGITKprPAIR	24.4	655.391	3+	0.50	O	
	**Kacac***VLRDNIQGITKprPAIR	36.8	655.390	3+	0.02	O	

*, $^{13}C_2$-acetoacetylation on lysine residue.

HepG2 and MCF7 cells were treated with 10 mM ethyl acetoacetate-1,3-$^{13}C_2$ for 24 hrs, and histones were extracted for MS analysis. $^{13}C_2$-Kacac sites were identified by PRM and quantified using peak areas of the precursor ions.

a

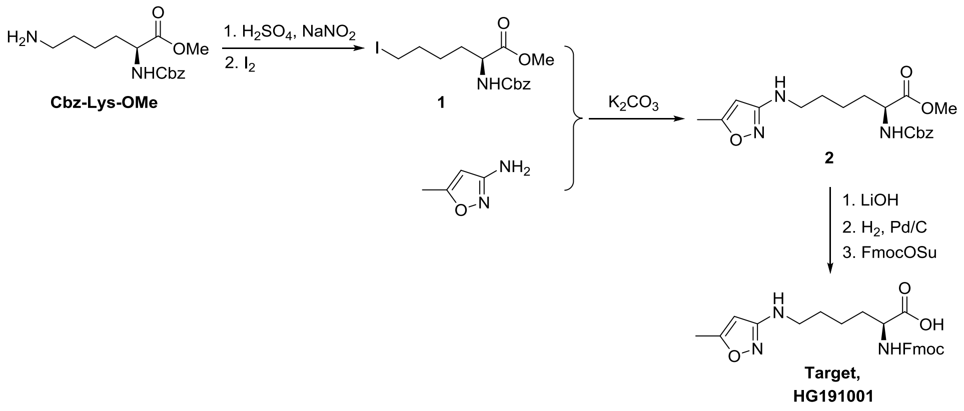

b

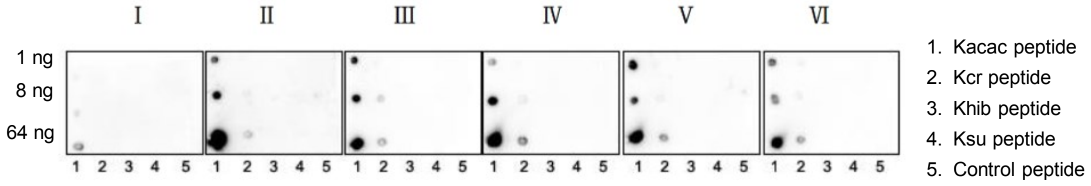

Figure S2. Generation and validation of pan anti-acetoacetyl-lysine antibody. (**a**) Workflow for synthesizing the structural analogs of Kacac. (**b**) Dot-blot assays for the purified antibody against Kacac, Kcr, Khib, Ksu, and unmodified K in six batches. For each group, 1, 8, and 64 ng of the indicated peptides were blotted.

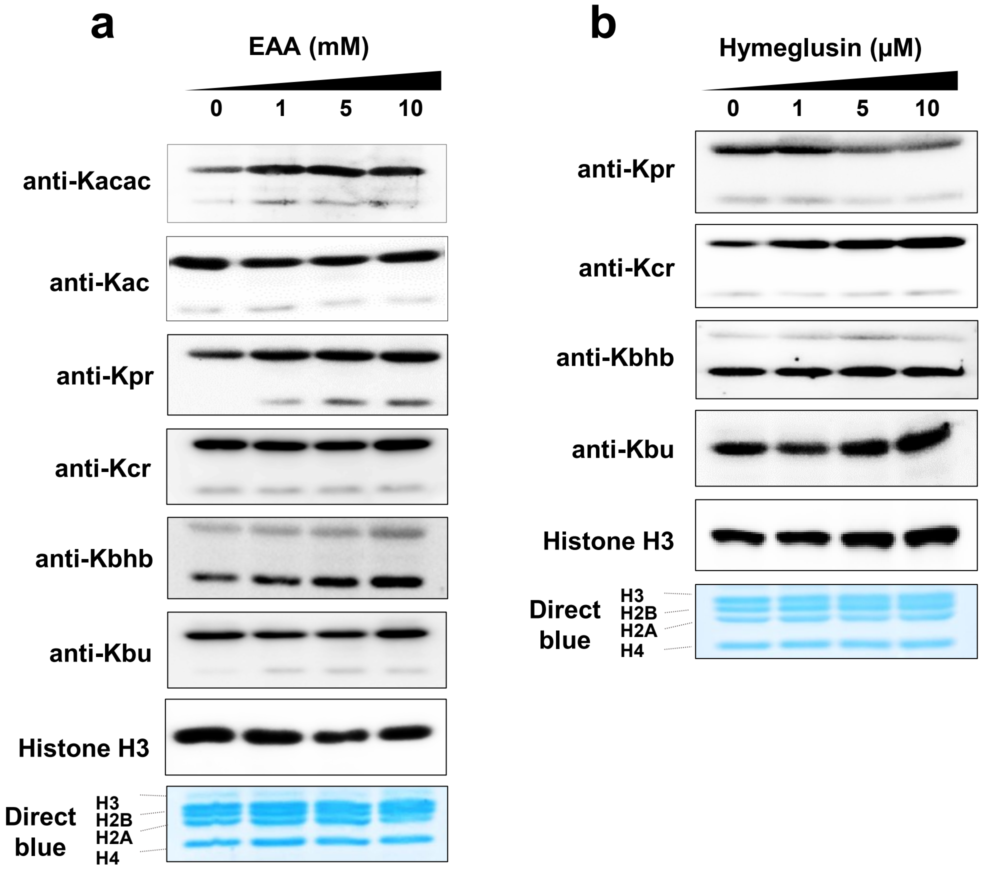

Figure S3. Dynamic regulation of histone acylations in response to acetoacetate and hymeglusin treatments. (**a**) Western blot of four lysine acylations (Kpr, Kcr, Kbhb, and Kbu) on histones from HepG2 cells after ethyl acetoacetate (EAA) treatment for 24 hrs. (**b**) Western blot of lysine acylations on HepG2 histones after hymeglusin treatment for 24 hrs. Kpr, propionyl-lysine; Kcr, crotonyl-lysine; Kbhb, beta-hydroxybutyryl-lysine; Kbu, butyryl-lysine.

Table S3. Quantification of Kacac peptides on histones from GCN5 or HBO1 OE HEK293T cells

Modified sites	Peptide sequences	GCN5/Mock	HBO1/Mock
		Ratio	Ratio
H2AK5	G**Kacac**QGGKprAR	**G only**	**H only**
H2BK16	**Kacac**AVTKacAQK	-	**H only**
H2BK34	**Kacac**ESYSVYVYK	0.63	**2.22**
H2BK86	LAHYS**Kacac**R	0.69	0.81
H3K9	**Kacac**STGGKprAPR	0.98	1.25
H3K18	**Kacac**QLATKprAAR	0.35	0.68
H3K27	**Kacac**SAPATGGVKprKprPHR	0.86	0.76
H3K79	EIAQDF**Kacac**TDLR	0.66	0.96
H4K12	GLG**Kacac**GGAKacR	**3.61**	**2.46**
H4K79	**Kacac**TVTAMDVVYALK	-	**3.50**

Histones were extracted from HEK293T cells transfected with GCN5 or HBO1 OE plasmids. Histone lysine acetoacetylation sites were detected by PRM and quantified using the peak areas of precursor ions. The samples were analyzed in duplicates (n = 2).

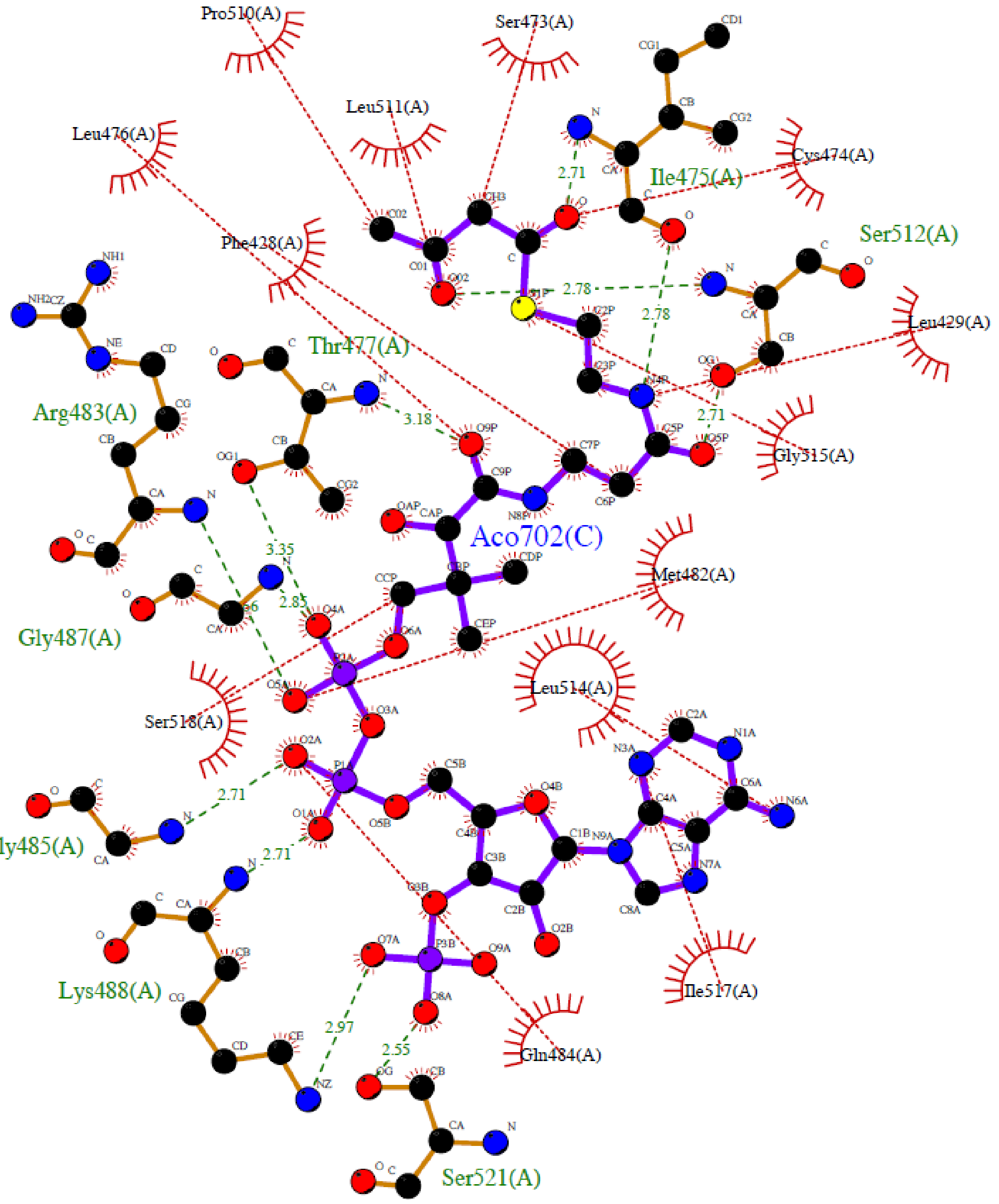

Figure S4. Predicted model of HBO1 binding to acetoacetyl-CoA. *In silico* molecular modeling of acetoacetyl-CoA and HBO1 interaction. Black: carbon; blue: nitrogen; red: oxygen; violet: phosphate. The backbone of acetoacetyl-CoA is highlighted in violet. Amino acid residues in proximity to acetoacetyl-CoA molecule are labeled, and the hydrogen bonds are indicated by green dashed lines.

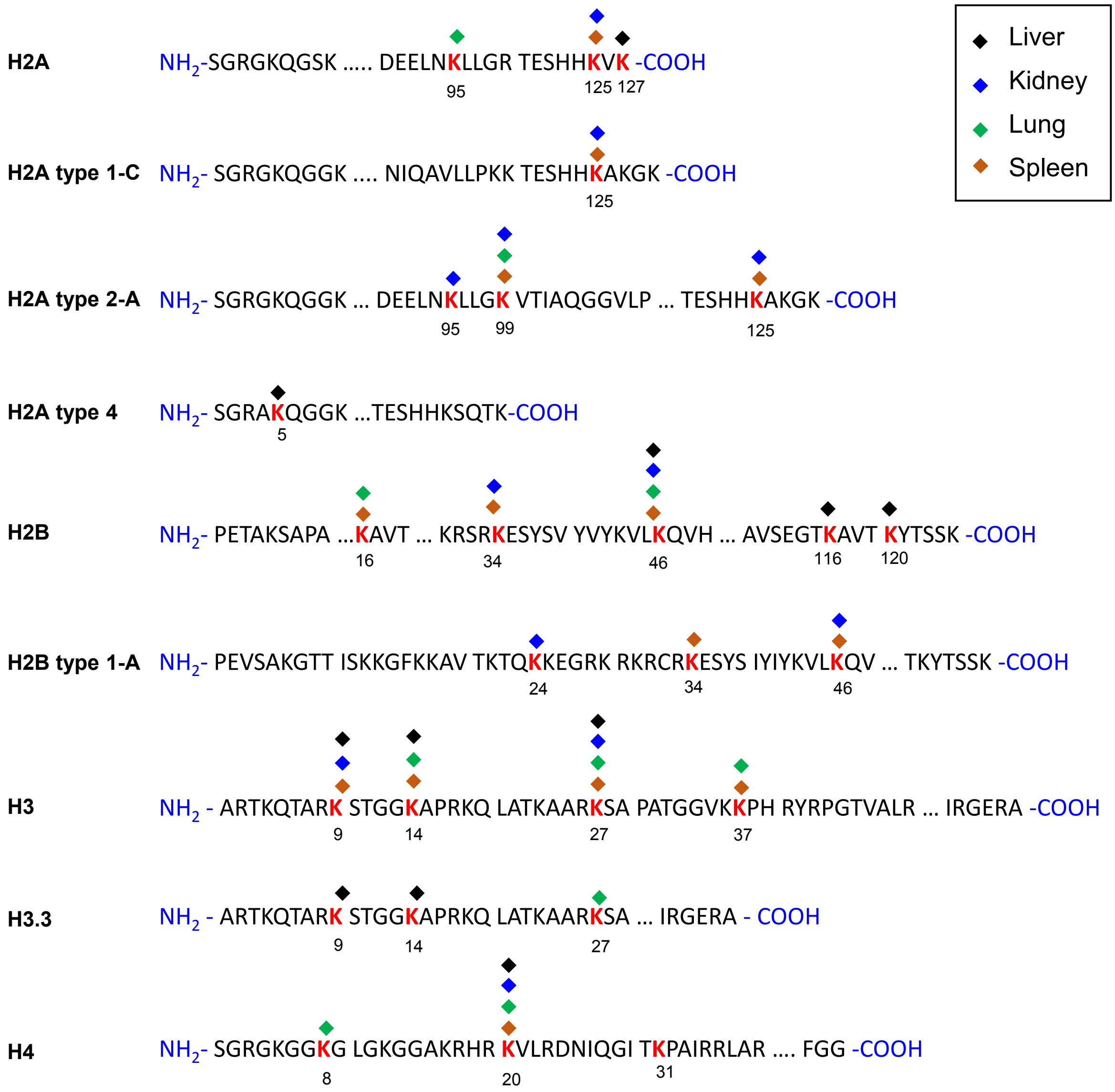

Figure S5. Map of the identified histone Kacac sites across rat organs. A summary of the identified Kacac sites on core histones extracted from the liver, kidney, lung, and spleen of *Rattus norvegicus*.

Table S4. A list of identified Kacac sites on histones from rat liver, kidney lung and spleen.

Sites	Detected sequences	Score	Observed m/z	Charge	Expect	Tissue			
						Liver	Kidney	Lung	Spleen
H2A_K95	NDEELN**Kacac**LLGKVTIAQGGVLPNIQAVLLPK	27.36	1090.2861	2	0.11			O	
H2A_K125	VTIAQGGVLPNIQAVLLPKmeKmeTESHH**Kacac**	26.63	723.4182	4	0.13		O		
	VTIAQGGVLPNIQAVLLPKacKacTESHH**Kacac**	26.04	737.4233	4	0.17				O
H2A_K127	KprTESHHKV**Kacac**	24.00	617.3344	2	1.2	O			
H2A type1C_K125	VTIAQGGVLPNIQAVLLPKmeKmeTESHH**Kacac**	26.63	723.4182	4	0.13		O		
	VTIAQGGVLPNIQAVLLPKacKacTESHH**Kacac**	26.04	737.4233	4	0.17				O
H2A type2A_K95	NDEELN**Kacac**LLGKVTIAQGGVLPNIQAVLLPK	27.36	1090.2861	3	0.11		O		
H2A type2A_K99	NDEELNKLLG**Kacac**VTIAQGGVLPNIQAVLLPK	38.8	817.9692	4	0.01		O		O
	NDEELNKLLG**Kacac**	23.5	678.8585	2	1.40			O	
H2A type2A_K125	VTIAQGGVLPNIQAVLLPKmeKmeTESHH**Kacac**	26.63	723.4182	4	0.13		O		
	VTIAQGGVLPNIQAVLLPKacKacTESHH**Kacac**	26.04	737.4233	4	0.17				O
H2A type4_K5	A**Kacac**QGGKpr	20.55	364.7015	2	1.60	O			
H2B_K16	**Kacac**AITKacAQKpr	24.36	535.3137	2	1.20			O	O
H2B_K34	SR**Kacac**ESYSVYVYK	23.47	796.8989	2	1.10		O		O
H2B_K46	VL**Kacac**QVHPDTGISSKpr	49	824.949	2	0.00	O	O	O	O
	VL**Kacac**QVHPDTGISSK	37.54	796.9308	2	0.06	O	O		
H2B_K116	HAVSEGT**Kacac**AVTKacacYTSSKpr	22.9	1009.519	2	1.80	O			
H2B_K120	HAVSEGTKacacAVT**Kacac**YTSSKpr	22.9	1009.519	2	1.80	O			
H2B type 1-A_K24	KprAVTKTQ**Kacac**	23.79	522.3111	2	1.50		O		

H2B type 1-A_K34	**Kacac**ESYSIYIYKprVLKpr	22.91	915.5016	2	1.70				O
H2B type 1-A_K46	VL**Kacac**QVHPDTGISSKpr	47.35	824.945	2	0.01		O		O
H3_K9	**Kacac**STGGKpr	28.29	359.1985	2	0.50	O	O		O
H3_K14	KprSTGG**Kacac**APR	22.88	521.2822	2	1.10	O		O	O
H3_K27	**Kacac**SAPATGGVKpr	48.93	528.2999	2	0.00	O	O	O	O
	**Kacac**SAPATGGVK	65.52	500.2847	2	0.00	O		O	
H3_K37	KacacSAPATGGVK**Kacac**PHR	21.82	534.6387	3	1.80			O	O
H3.3_K9	**Kacac**STGGKpr	28.29	359.1985	2	0.50	O			
H3.3_K14	KprSTGG**Kacac**APR	20.36	521.2818	2	2.00	O			
H3.3_K27	**Kacac**SAPSTGGVKprKprPHR	24.3	549.315	3	0.87			O	
H4_K8	GG**Kacac**GLGKprGGAKpr	20.63	563.3187	2	2.00			O	
H4_K20	**Kacac**VLRDNIQGITKprPAIR	45.63	981.5924	2	0.00	O	O	O	O
	**Kacac**VLRDNIQGITKPAIR	28.14	636.056	3	0.06	O	O	O	O
H4_K31	**Kacac**VLRDNIQGITKacacPAIR	43.26	981.59	2	0.00		O		

Histone lysine acetoacetylation sites were identified by PRM. Cut-off: peptide score ≥ 20, expect value ≤ 2.

Figure S6. Representative MS[2] spectra of the identified 33 Kacac sites.

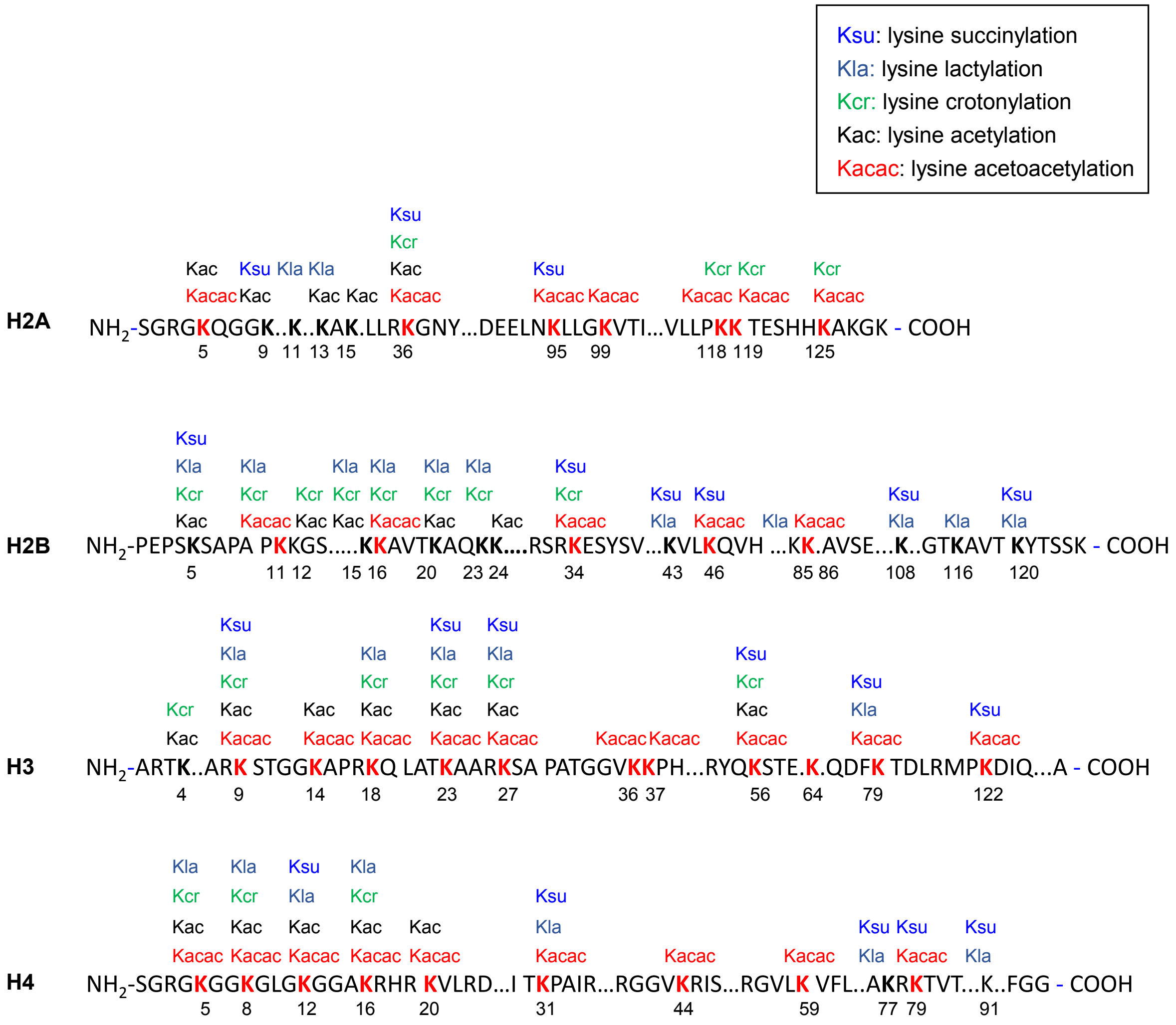

Figure S7. Overlapping histone Kacac sites with succinylation (Ksu), lactylation (Kla), crotonylation (Kcr), and acetylation (Kac).

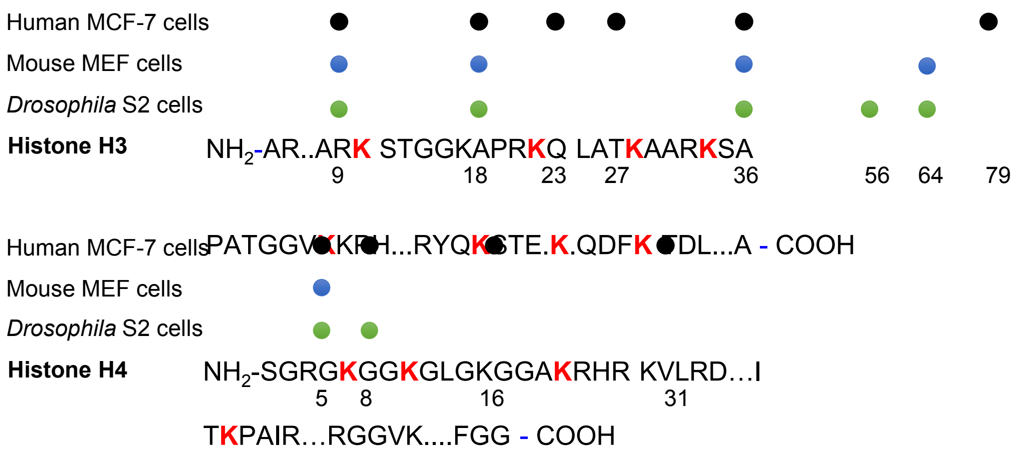

Figure S8. Map of the identified Kacac sites on histones H3 and H4 in human MCF7, mouse MEF, and *Drosophila* S2 cells.

References

[1] Shechter D, Dormann HL, Allis CD, Hake SB. Extraction, purification and analysis of histones. Nat Protoc 2007;2:1445-57.

[2] Garcia BA, Shabanowitz J, Hunt DF. Characterization of histones and their post-translational modifications by mass spectrometry. Curr Opin Chem Biol 2007;11:66-73.

[3] Huang H, Lin S, Garcia BA, Zhao Y. Quantitative proteomic analysis of histone modifications. Chem Rev 2015;115:2376-418.

[4] Tan M, Luo H, Lee S, Jin F, Yang JS, Montellier E, et al. Identification of 67 histone marks and histone lysine crotonylation as a new type of histone modification. Cell 2011;146:1016-28.

[5] Adamczyk M, Johnson DD, Reddy RE. Collagen cross-links. Synthesis of immunoreagents for development of assays for deoxypyridinoline, a marker for diagnosis of osteoporosis. Bioconjug Chem 2000;11:124-30.

[6] El-Serwy WS, Mohamed NA, EI-serwy WS, Mahmoud AH. Synthesis, biological evaluation and molecular modeling studies of some 5-methylisoxazole derivatives as anti-inflammatory agents. J Chem Pharm Re. 2017;9:117-27.

[7] Liu F, Thomas J, Burke TR, Jr. Synthesis of a Homologous Series of Side Chain Extended Orthogonally-Protected Aminooxy-Containing Amino Acids. Synthesis (Stuttg) 2008;15:2432-8.

[8] Qiang L, Xiao H, Campos EI, Ho VC, Li G. Development of a PAN-specific, affinity-purified anti-acetylated lysine antibody for detection, identification, isolation, and intracellular localization of acetylated protein. J Immunoassay Immunochem 2005;26:13-23.

[9] Guan KL, Yu W, Lin Y, Xiong Y, Zhao S. Generation of acetyllysine antibodies and affinity enrichment of acetylated peptides. Nat Protoc 2010;5:1583-95.

[10] Garcia BA, Mollah S, Ueberheide BM, Busby SA, Muratore TL, Shabanowitz J, et al. Chemical derivatization of histones for facilitated analysis by mass spectrometry. Nat Protoc 2007;2:933-8.

[11] Perez-Riverol Y, Csordas A, Bai J, Bernal-Llinares M, Hewapathirana S, Kundu DJ, et al. The PRIDE database and related tools and resources in 2019: improving support for quantification data. Nucleic Acids Res 2019;47:D442-D50.

[12] Morris GM, Huey R, Lindstrom W, Sanner MF, Belew RK, Goodsell DS, et al. AutoDock4 and AutoDockTools4: Automated docking with selective receptor flexibility. J Comput Chem 2009;30:2785-91.
[13] Laskowski RA, Swindells MB. LigPlot+: multiple ligand-protein interaction diagrams for drug discovery. J Chem Inf Model 2011;51:2778-86.
