# Supplementary material for: Identification of Histone Lysine Acetoacetylation as a Dynamic Post‐Translational Modification Regulated by HBO1
Source: Adv Sci (Weinh). 2023 Jun 29;10(25):2300032. doi: 10.1002/advs.202300032 (PMC10477889; doi:10.1002/advs.202300032)
Supplement: Supplementary file 2 — Supporting Information [file ADVS-10-2300032-s001.pdf]

ADVANCED SCIENCE
Open Access

Supporting Information

for *Adv. Sci.*, DOI 10.1002/advs.202300032

Identification of Histone Lysine Acetoacetylation as a Dynamic Post-Translational Modification Regulated by HBO1

Yan Gao, Xinlei Sheng, Doudou Tan, SunJoo Kim, Soyoung Choi, Sanjita Paudel, Taeho Lee, Cong Yan, Minjia Tan, Kyu Min Kim, Sam Seok Cho, Sung Hwan Ki, He Huang, Yingming Zhao* and Sangkyu Lee**

Figure S6. Representative MS^2 spectra of the identified 33 Kacac sites

H2AK5	GKacacQGGKprAR	Human HepG2 cells

MS/MS Fragmentation of GKQGGKAR
Found in A0A0U1RR32, Histone H2A OS=Homo sapiens OX=9606 GN=hCG_2039566 PE=3 SV=1

Match to Query 2585: 940.505448 from(471.260000,2+)
Title: File:20201204_C12_HepG2_ingel_1_1.mzXML Scans:4306 RT:31.9268min Charge:2+ Fragmentation:cid
Data file 20201204_C12_HepG2_ingel_1_1.mgf

Click mouse within plot area to zoom in by factor of two about that point
Or, Plot from 100 to 950 Da Full range
Label all possible matches ○ Label matches used for scoring ◉

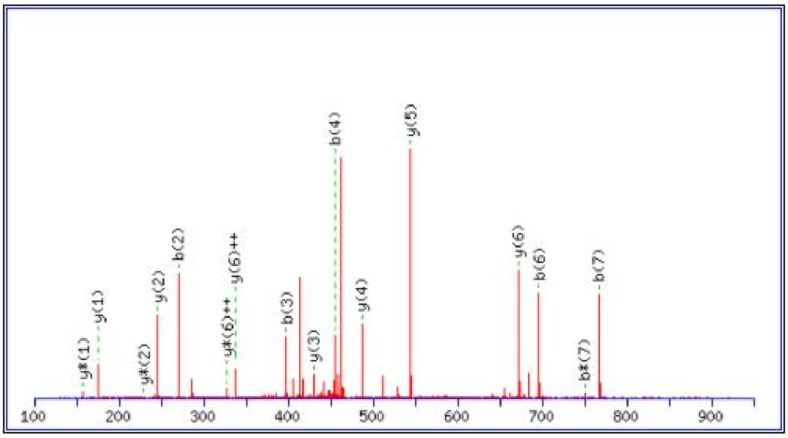

Monoisotopic mass of neutral peptide Mr(calc): 940.5090
Variable modifications:
K2 : Acetoacetyl (K)
K6 : Propionyl (K)
Ions Score: 36 **Expect:** 0.046
Matches : 16/54 fragment ions using 32 most intense peaks (help)

#	b	b++	b*	b*++	Seq.	y	y++	y*	y*++	#
1	58.0287	29.5180			G					8
2	270.1448	135.5761	253.1183	127.0628	K	884.4948	442.7511	867.4683	434.2378	7
3	398.2034	199.6053	381.1769	191.0921	Q	672.3787	336.6930	655.3522	328.1797	6
4	455.2249	228.1161	438.1983	219.6028	G	544.3202	272.6637	527.2936	264.1504	5
5	512.2463	256.6268	495.2198	248.1135	G	487.2987	244.1530	470.2722	235.6397	4
6	696.3675	348.6874	679.3410	340.1741	K	430.2772	215.6423	413.2507	207.1290	3
7	767.4046	384.2060	750.3781	375.6927	A	246.1561	123.5817	229.1295	115.0684	2
8					R	175.1190	88.0631	158.0924	79.5498	1

H2AK36	KacacGNYSER	Human HepG2 cells

MS/MS Fragmentation of **KGNYSER**
Found in **A0A0U1RR32**, Histone H2A OS=Homo sapiens OX=9606 GN=hCG_2039566 PE=3 SV=1

Match to Query 2520: 936.426248 from(469.220400,2+)
Title: File:20201204_C12_HepG2_ingel_1_1.mzXML Scans:5692 RT:37.0247min Charge:2+ Fragmentation:cid
Data file 20201204_C12_HepG2_ingel_1_1.mgf

Click mouse within plot area to zoom in by factor of two about that point
Or, Plot from 100 to 900 Da Full range
Label all possible matches ○ Label matches used for scoring ◉

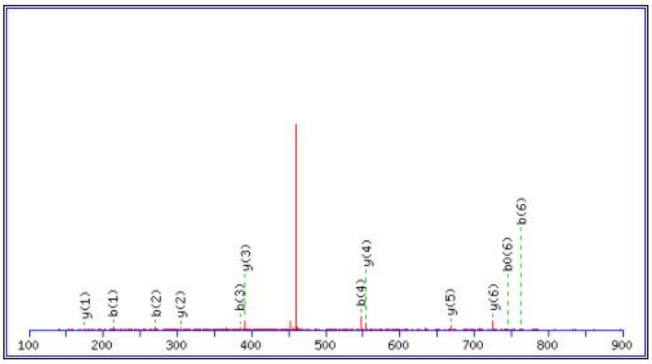

Monoisotopic mass of neutral peptide Mr(calc): 936.4301
Variable modifications:
K1 : Acetoacetyl (K)
Ions Score: 59 **Expect:** 8.5e-005
Matches : 12/62 fragment ions using 16 most intense peaks (help)

#	b	b++	b*	b*++	b0	b0++	Seq.	y	y++	y*	y*++	y0	y0++	#
1	**213.1234**	107.0653	196.0968	98.5520			K							7
2	**270.1448**	135.5761	253.1183	127.0628			G	***725.3213***	363.1643	708.2947	354.6510	707.3107	354.1590	6
3	**384.1878**	192.5975	367.1612	184.0842			N	***668.2998***	334.6536	651.2733	326.1403	650.2893	325.6483	5
4	**547.2511**	274.1292	530.2245	265.6159			Y	***554.2569***	277.6321	537.2304	269.1188	536.2463	268.6268	4
5	634.2831	317.6452	617.2566	309.1319	616.2726	308.6399	S	***391.1936***	196.1004	374.1670	187.5872	373.1830	187.0951	3
6	**763.3257**	382.1665	746.2992	373.6532	745.3151	373.1612	E	***304.1615***	152.5844	287.1350	144.0711	286.1510	143.5791	2
7							R	***175.1190***	88.0631	158.0924	79.5498			1

H2AK95	NDEELNKacacLLGKpr	Human HepG2 cells

MS/MS Fragmentation of **NDEELNKLLGK**
Found in **A0A0U1RR32**, Histone H2A OS=Homo sapiens OX=9606 GN=hCG_2039566 PE=3 SV=1

Match to Query 11841: 1411.720048 from(706.867300,2+)
Title: File:20201204_C12_HepG2_ingel_3_1.mzXML Scans:27534 RT:117.0000min Charge:2+ Fragmentation:cid
Data file 20201204_C12_HepG2_ingel_3_1.mgf

Click mouse within plot area to zoom in by factor of two about that point
Or, Plot from 200 to 1500 Da Full range
Label all possible matches ○ Label matches used for scoring ◉

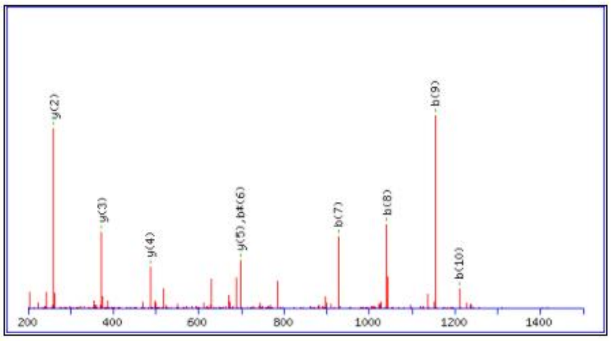

Monoisotopic mass of neutral peptide Mr(calc): 1411.7194
Variable modifications:
K7 : Acetoacetyl (K)
K11 : Propionyl (K)
Ions Score: 25 **Expect:** 0.16
Matches : 9/104 fragment ions using 18 most intense peaks (help)

#	b	b^{++}	b^*	b^{*++}	b^0	b^{0++}	Seq	y	y^{++}	y^*	y^{*++}	y^0	y^{0++}	#
1	115.0502	58.0287	98.0237	49.5155			N							11
2	230.0771	115.5422	213.0506	107.0289	212.0666	106.5369	D	1298.6838	649.8455	1281.6573	641.3323	1280.6733	640.8403	10
3	359.1197	180.0635	342.0932	171.5502	341.1092	171.0582	E	1183.6569	592.3321	1166.6303	583.8188	1165.6463	583.3268	9
4	488.1623	244.5848	471.1358	236.0715	470.1518	235.5795	E	1054.6143	527.8108	1037.5877	519.2975	1036.6037	518.8055	8
5	601.2464	301.1268	584.2198	292.6136	583.2358	292.1216	L	925.5717	463.2895	908.5451	454.7762			7
6	715.2893	358.1483	698.2628	349.6350	697.2788	349.1430	N	812.4876	406.7475	795.4611	398.2342			6
7	***927.4054***	464.2063	910.3789	455.6931	909.3949	455.2011	K	***698.4447***	349.7260	681.4182	341.2127			5
8	***1040.4895***	520.7484	1023.4629	512.2351	1022.4789	511.7431	L	***486.3286***	243.6679	469.3021	235.1547			4
9	***1153.5735***	577.2904	1136.5470	568.7771	1135.5630	568.2851	L	***373.2445***	187.1259	356.2180	178.6126			3
10	***1210.5950***	605.8011	1193.5685	597.2879	1192.5844	596.7959	G	***260.1605***	130.5839	243.1339	122.0706			2
11							K	203.1390	102.0731	186.1125	93.5599			1

H2AK99	NDEELNKLLGKacac	Human HepG2 cells

MS/MS Fragmentation of **NDEELNKLLGK**
Found in **A0A0U1RR32**, Histone H2A OS=Homo sapiens OX=9606 GN=hCG_2039566 PE=3 SV=1

Match to Query 8405: 1355.692648 from(678.853600,2+)
Title: File:20201204_C12_HepG2_ingel_1_1.mzXML Scans:16011 RT:73.7993min Charge:2+ Fragmentation:cid
Data file 20201204_C12_HepG2_ingel_1_1.mgf

Click mouse within plot area to zoom in by factor of two about that point
Or, Plot from 100 to 1400 Da Full range
Label all possible matches ○ Label matches used for scoring ◉

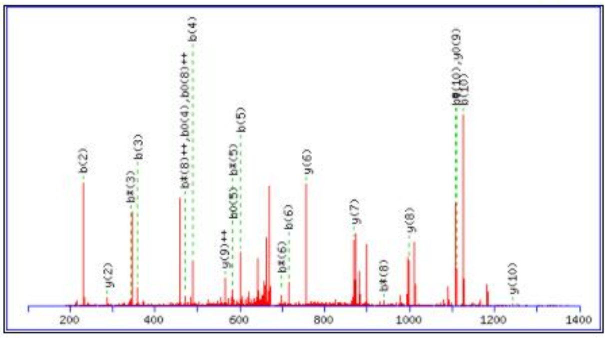

Monoisotopic mass of neutral peptide Mr(calc): 1355.6932
Variable modifications:
K11 : Acetoacetyl (K)
Ions Score: 27 **Expect:** 1
Matches : 23/104 fragment ions using 48 most intense peaks (help)

#	b	b^{++}	b^{*}	b^{*++}	b^{0}	b^{0++}	Seq	y	y^{++}	y^{*}	y^{*++}	y^{0}	y^{0++}	#
1	115.0502	58.0287	98.0237	49.5155			N							11
2	***230.0771***	115.5422	213.0506	107.0289	212.0666	106.5369	D	***1242.6576***	621.8324	1225.6311	613.3192	1224.6470	612.8272	10
3	***359.1197***	180.0635	342.0932	171.5502	341.1092	171.0582	E	1127.6307	564.3190	1110.6041	555.8057	1109.6201	555.3137	9
4	***488.1623***	244.5848	471.1358	236.0715	470.1518	235.5795	E	***998.5881***	499.7977	981.5615	491.2844	980.5775	490.7924	8
5	***601.2464***	301.1268	584.2198	292.6136	583.2358	292.1216	L	***869.5455***	435.2764	852.5189	426.7631			7
6	***715.2893***	358.1483	698.2628	349.6350	697.2788	349.1430	N	***756.4614***	378.7343	739.4349	370.2211			6
7	843.3843	422.1958	826.3577	413.6825	825.3737	413.1905	K	642.4185	321.7129	625.3919	313.1996			5
8	956.4684	478.7378	939.4418	470.2245	938.4578	469.7325	L	514.3235	257.6654	497.2970	249.1521			4
9	1069.5524	535.2798	1052.5259	526.7666	1051.5418	526.2746	L	401.2395	201.1234	384.2129	192.6101			3
10	***1126.5739***	563.7906	1109.5473	555.2773	1108.5633	554.7853	G	***288.1554***	144.5813	271.1288	136.0681			2
11							K	231.1339	116.0706	214.1074	107.5573			1

H2AK118	ATIAGGGVIPHIHKacSLIGKacacKac	Rat liver

MS/MS Fragmentation of **ATIAGGGVIPHIHKSLIGKK**
Found in **A0A0A0MXW3**, Histone H2A OS=Rattus norvegicus OX=10116 GN=H2afz PE=3 SV=1
Match to Query 8799: 2164.270096 from(542.074800,4+)
Title: File:20200819_Histone_IP.mzXML Scans:13266 RT:53.8495min Charge:4+ Fragmentation:cid
Data file 20200819_Histone_IP_liver.mgf

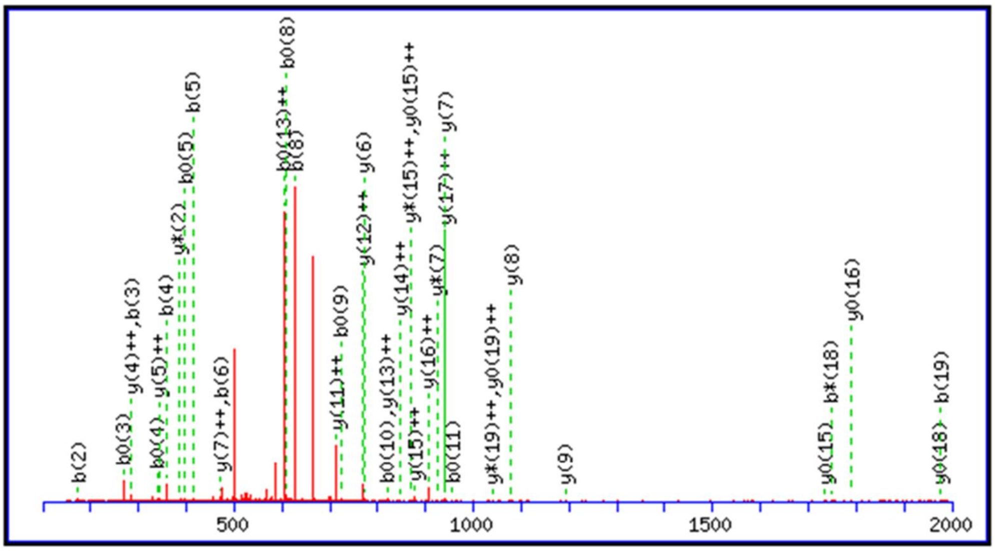

Monoisotopic mass of neutral peptide Mr(calc): 2164.2368
Variable modifications: K14 : Acetyl (K) **K19 : Acetoacetyl (K)** **K20 :** Acetyl (K)
Ions Score: 21 **Expect:** 0.16
Matches : 39/190 fragment ions using 126 most intense peaks

#	b	b^{++}	b^{*}	b^{*++}	b^{0}	b^{0++}	Seq.	y	y^{++}	y^{*}	y^{*++}	y^{0}	y^{0++}	#
1	72.0444	36.5258					A							20
2	**173.0921**	87.0497			155.0815	78.0444	T	2094.2070	1047.6071	2077.1804	1039.0939	2076.1964	1038.6018	19
3	**286.1761**	143.5917			268.1656	134.5864	I	1993.1593	997.0833	1976.1328	988.5700	1975.1487	988.0780	18
4	**357.2132**	179.1103			339.2027	170.1050	A	1880.0752	940.5413	1863.0487	932.0280	1862.0647	931.5360	17
5	**414.2347**	207.6210			396.2241	198.6157	G	1809.0381	905.0227	1792.0116	896.5094	1791.0276	896.0174	16
6	**471.2562**	236.1317			453.2456	227.1264	G	1752.0167	876.5120	1734.9901	867.9987	1734.0061	867.5067	15
7	528.2776	264.6425			510.2671	255.6372	G	1694.9952	848.0012	1677.9687	839.4880	1676.9846	838.9960	14
8	**627.3461**	314.1767			609.3355	305.1714	V	1637.9737	819.4905	1620.9472	810.9772	1619.9632	810.4852	13
9	740.4301	370.7187			722.4196	361.7134	I	1538.9053	769.9563	1521.8788	761.4430	1520.8948	760.9510	12
10	837.4829	419.2451			819.4723	410.2398	P	1425.8213	713.4143	1408.7947	704.9010	1407.8107	704.4090	11
11	974.5418	487.7745			956.5312	478.7693	H	1328.7685	664.8879	1311.7419	656.3746	1310.7579	655.8826	10
12	1087.6259	544.3166			1069.6153	535.3113	I	**1191.7096**	596.3584	1174.6830	587.8452	1173.6990	587.3531	9
13	1224.6848	612.8460			1206.6742	603.8407	H	**1078.6255**	539.8164	1061.5990	531.3031	1060.6150	530.8111	8
14	1394.7903	697.8988	1377.7637	689.3855	1376.7797	688.8935	K	**941.5666**	471.2869	924.5401	462.7737	923.5560	462.2817	7
15	1481.8223	741.4148	1464.7958	732.9015	1463.8118	732.4095	S	**771.4611**	386.2342	754.4345	377.7209	753.4505	377.2289	6
16	1594.9064	797.9568	1577.8798	789.4436	1576.8958	788.9516	L	684.4291	342.7182	667.4025	334.2049			5
17	1707.9905	854.4989	1690.9639	845.9856	1689.9799	845.4936	I	571.3450	286.1761	554.3184	277.6629			4
18	1765.0119	883.0096	1747.9854	874.4963	1747.0014	874.0043	G	458.2609	229.6341	441.2344	221.1208			3
19	**1977.1280**	989.0676	1960.1015	980.5544	1959.1174	980.0624	K	401.2395	201.1234	384.2129	192.6101			2
20							K	189.1234	95.0653	172.0968	86.5520			1

**H2AK119**	**ATIAGGGVIPHIHKacSLIGKacKacac**	**Rat liver**

MS/MS Fragmentation of **ATIAGGGVIPHIHKSLIGKK**
Found in **A0A0A0MXW3**, Histone H2A OS=Rattus norvegicus OX=10116 GN=H2afz PE=3 SV=1
Match to Query 7273: 2164.273696 from(542.075700,4+)
Title: File:20200819_Histone_IP_Kidney.mzXML Scans:11454 RT:51.3880min Charge:4+ Fragmentation:cid
Data file 20200819_Histone_IP_Kidney.mgf

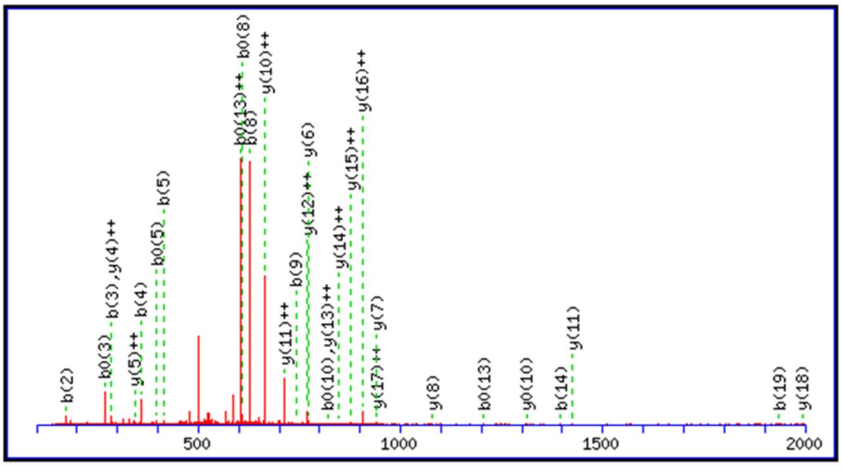

Monoisotopic mass of neutral peptide Mr(calc): 2164.2368
Variable modifications: K14 : Acetyl (K) **K19 :** Acetyl (K) **K20 : Acetoacetyl (K)**
Ions Score: 31 **Expect:** 0.038
Matches : 30/190 fragment ions using 86 most intense peaks

#	b	b^{++}	b^{*}	b^{*++}	b^{0}	b^{0++}	Seq.	y	y^{++}	y^{*}	y^{*++}	y^{0}	y^{0++}	#
1	72.0444	36.5258					A							20
2	173.0921	87.0497			155.0815	78.0444	T	2094.2070	1047.6071	2077.1804	1039.0939	2076.1964	1038.6018	19
3	286.1761	143.5917			268.1656	134.5864	I	1993.1593	997.0833	1976.1328	988.5700	1975.1487	988.0780	18
4	357.2132	179.1103			339.2027	170.1050	A	1880.0752	940.5413	1863.0487	932.0280	1862.0647	931.5360	17
5	414.2347	207.6210			396.2241	198.6157	G	1809.0381	905.0227	1792.0116	896.5094	1791.0276	896.0174	16
6	471.2562	236.1317			453.2456	227.1264	G	1752.0167	876.5120	1734.9901	867.9987	1734.0061	867.5067	15
7	528.2776	264.6425			510.2671	255.6372	G	1694.9952	848.0012	1677.9687	839.4880	1676.9846	838.9960	14
8	627.3461	314.1767			609.3355	305.1714	V	1637.9737	819.4905	1620.9472	810.9772	1619.9632	810.4852	13
9	740.4301	370.7187			722.4196	361.7134	I	1538.9053	769.9563	1521.8788	761.4430	1520.8948	760.9510	12
10	837.4829	419.2451			819.4723	410.2398	P	1425.8213	713.4143	1408.7947	704.9010	1407.8107	704.4090	11
11	974.5418	487.7745			956.5312	478.7693	H	1328.7685	664.8879	1311.7419	656.3746	1310.7579	655.8826	10
12	1087.6259	544.3166			1069.6153	535.3113	I	1191.7096	596.3584	1174.6830	587.8452	1173.6990	587.3531	9
13	1224.6848	612.8460			1206.6742	603.8407	H	1078.6255	539.8164	1061.5990	531.3031	1060.6150	530.8111	8
14	1394.7903	697.8988	1377.7637	689.3855	1376.7797	688.8935	K	941.5666	471.2869	924.5401	462.7737	923.5560	462.2817	7
15	1481.8223	741.4148	1464.7958	732.9015	1463.8118	732.4095	S	771.4611	386.2342	754.4345	377.7209	753.4505	377.2289	6
16	1594.9064	797.9568	1577.8798	789.4436	1576.8958	788.9516	L	684.4291	342.7182	667.4025	334.2049			5
17	1707.9905	854.4989	1690.9639	845.9856	1689.9799	845.4936	I	571.3450	286.1761	554.3184	277.6629			4
18	1765.0119	883.0096	1747.9854	874.4963	1747.0014	874.0043	G	458.2609	229.6341	441.2344	221.1208			3
19	1935.1174	968.0624	1918.0909	959.5491	1917.1069	959.0571	K	401.2395	201.1234	384.2129	192.6101			2
20							K	231.1339	116.0706	214.1074	107.5573			1

H2AK125	VTIAQGGVLPNIQAVLLPKmeKmeTESHHKacac	Human HepG2 cells

MS/MS Fragmentation of VTIAQGGVLPNIQAVLLPKKTESHHK
Found in A0A0U1RR32, Histone H2A OS=Homo sapiens OX=9606 GN=hCG_2039566 PE=3 SV=1

Match to Query 20230: 2889.642096 from(723.417800,4+)
Title: File:20201204_C12_HepG2_ingel_1_1.mzXML Scans:20376 RT:89.3377min Charge:4+ Fragmentation:cid
Data file 20201204_C12_HepG2_ingel_1_1.mgf

Click mouse within plot area to zoom in by factor of two about that point
Or, Plot from 200 to 2000 Da Full range
Label all possible matches ○ Label matches used for scoring ◉

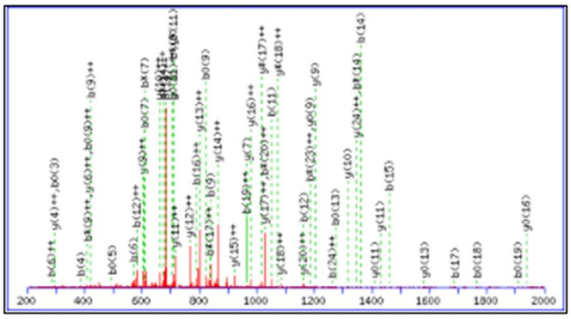

Monoisotopic mass of neutral peptide Mr(calc): 2889.6440
Variable modifications:
K19 : Methyl (KR)
K20 : Methyl (KR)
K26 : Acetoacetyl (K)
Ions Score: 64 **Expect:** 0.0018
Matches : 68/284 fragment ions using 181 most intense peaks (help)

#	b	b++	b*	b*++	b0	b0++	Seq.	y	y++	y*	y*++	y0	y0++	#
1	100.0757	50.5415					V							26
2	201.1234	101.0653			183.1128	92.0600	T	2791.5829	1396.2951	2774.5563	1387.7818	2773.5723	1387.2898	25
3	314.2074	157.6074			296.1969	148.6021	I	2690.5352	1345.7712	2673.5086	1337.2580	2672.5246	1336.7660	24
4	385.2445	193.1259			367.2340	184.1206	A	2577.4511	1289.2292	2560.4246	1280.7159	2559.4406	1280.2239	23
5	513.3031	257.1552	496.2766	248.6419	495.2926	248.1499	Q	2506.4140	1253.7106	2489.3875	1245.1974	2488.4035	1244.7054	22
6	570.3246	285.6659	553.2980	277.1527	552.3140	276.6607	G	2378.3554	1189.6814	2361.3289	1181.1681	2360.3449	1180.6761	21
7	627.3461	314.1767	610.3195	305.6634	609.3355	305.1714	G	2321.3340	1161.1706	2304.3074	1152.6574	2303.3234	1152.1653	20
8	726.4145	363.7109	709.3879	355.1976	708.4039	354.7056	V	2264.3125	1132.6599	2247.2860	1124.1466	2246.3019	1123.6546	19
9	839.4985	420.2529	822.4720	411.7396	821.4880	411.2476	L	2165.2441	1083.1257	2148.2176	1074.6124	2147.2335	1074.1204	18
10	936.5513	468.7793	919.5247	460.2660	918.5407	459.7740	P	2052.1600	1026.5837	2035.1335	1018.0704	2034.1495	1017.5784	17
11	1050.5942	525.8007	1033.5677	517.2875	1032.5837	516.7955	N	1955.1073	978.0573	1938.0807	969.5440	1937.0967	969.0520	16
12	1163.6783	582.3428	1146.6517	573.8295	1145.6677	573.3375	I	1841.0643	921.0358	1824.0378	912.5225	1823.0538	912.0305	15
13	1291.7369	646.3721	1274.7103	637.8588	1273.7263	637.3668	Q	1727.9803	864.4938	1710.9537	855.9805	1709.9697	855.4885	14
14	1362.7740	681.8906	1345.7474	673.3774	1344.7634	672.8853	A	1599.9217	800.4645	1582.8952	791.9512	1581.9111	791.4592	13
15	1461.8424	731.4248	1444.8158	722.9116	1443.8318	722.4196	V	1528.8846	764.9459	1511.8580	756.4327	1510.8740	755.9406	12
16	1574.9265	787.9669	1557.8999	779.4536	1556.9159	778.9616	L	1429.8162	715.4117	1412.7896	706.8985	1411.8056	706.4064	11
17	1688.0105	844.5089	1670.9840	835.9956	1670.0000	835.5036	L	1316.7321	658.8697	1299.7056	650.3564	1298.7215	649.8644	10
18	1785.0633	893.0353	1768.0367	884.5220	1767.0527	884.0300	P	1203.6480	602.3277	1186.6215	593.8144	1185.6375	593.3224	9
19	1927.1739	964.0906	1910.1473	955.5773	1909.1633	955.0853	K	1106.5953	553.8013	1089.5687	545.2880	1088.5847	544.7960	8
20	2069.2845	1035.1459	2052.2580	1026.6326	2051.2739	1026.1406	K	964.4847	482.7460	947.4581	474.2327	946.4741	473.7407	7
21	2170.3322	1085.6697	2153.3056	1077.1565	2152.3216	1076.6644	T	822.3741	411.6907	805.3475	403.1774	804.3635	402.6854	6
22	2299.3748	1150.1910	2282.3482	1141.6778	2281.3642	1141.1857	E	721.3264	361.1668	704.2998	352.6536	703.3158	352.1615	5
23	2386.4068	1193.7070	2369.3803	1185.1938	2368.3962	1184.7018	S	592.2838	296.6455	575.2572	288.1323	574.2732	287.6402	4
24	2523.4657	1262.2365	2506.4392	1253.7232	2505.4552	1253.2312	H	505.2518	253.1295	488.2252	244.6162			3
25	2660.5246	1330.7660	2643.4981	1322.2527	2642.5141	1321.7607	H	368.1928	184.6001	351.1663	176.0868			2
26							K	231.1339	116.0706	214.1074	107.5573			1

H2AK127	KprTESHHKVKacac	Rat liver

MS/MS Fragmentation of **KTESHHKVK**
Found in **M0RDM4**, Histone H2A OS=Rattus norvegicus OX=10116 GN=LOC680322 PE=3 SV=1
Match to Query 3046: 1232.654248 from(617.334400,2+)
Title: File:20200819_Histone_IP.mzXML Scans:1001 RT:6.0979min Charge:2+ Fragmentation:cid
Data file 20200819_Histone_IP_liver.mgf

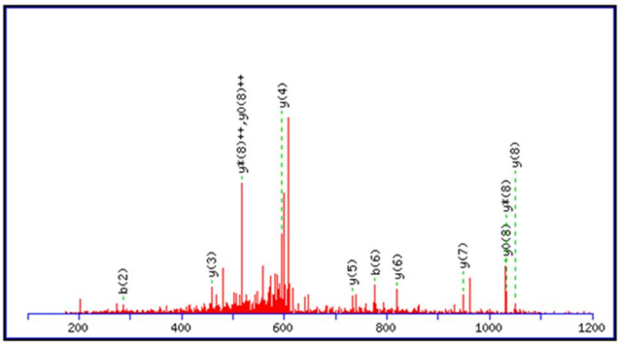

Monoisotopic mass of neutral peptide Mr(calc): 1232.6513 **Variable modifications: K1 :** Propionyl (K) **K9 :** Acetoacetyl (K) **Ions Score:** 24 **Expect:** 1.2 **Matches :** 12/84 fragment ions using 33 most intense peaks

#	b	b^{++}	b^{*}	b^{*++}	b^{0}	b^{0++}	Seq.	y	y^{++}	y^{*}	y^{*++}	y^{0}	y^{0++}	#
1	185.1285	93.0679	168.1019	84.5546			K							9
2	**286.1761**	143.5917	269.1496	135.0784	268.1656	134.5864	T	***1049.5374***	525.2724	1032.5109	516.7591	1031.5269	516.2671	8
3	415.2187	208.1130	398.1922	199.5997	397.2082	199.1077	E	***948.4898***	474.7485	931.4632	466.2352	930.4792	465.7432	7
4	502.2508	251.6290	485.2242	243.1157	484.2402	242.6237	S	***819.4472***	410.2272	802.4206	401.7139	801.4366	401.2219	6
5	639.3097	320.1585	622.2831	311.6452	621.2991	311.1532	H	***732.4151***	366.7112	715.3886	358.1979			5
6	**776.3686**	388.6879	759.3420	380.1747	758.3580	379.6826	H	***595.3562***	298.1817	578.3297	289.6685			4
7	904.4635	452.7354	887.4370	444.2221	886.4530	443.7301	K	***458.2973***	229.6523	441.2708	221.1390			3
8	1003.5320	502.2696	986.5054	493.7563	985.5214	493.2643	V	330.2023	165.6048	313.1758	157.0915			2
9							K	231.1339	116.0706	214.1074	107.5573			1

H2BK11	SAPAPKacacKGSK	Human HepG2 cells

MS/MS Fragmentation of **SAPAPKKGSK**
Found in **O60814**, Histone H2B type 1-K OS=Homo sapiens OX=9606 GN=HIST1H2BK PE=1 SV=3

Match to Query 5533: 1053.581448 from(527.798000,2+)
Title: File:20201204_C12_HepG2_ingel_3_1.mzXML Scans:1712 RT:18.1012min Charge:2+ Fragmentation:cid
Data file 20201204_C12_HepG2_ingel_3_1.mgf

Click mouse within plot area to zoom in by factor of two about that point
Or, Plot from 150 to 1100 Da Full range
Label all possible matches ○ Label matches used for scoring ◉

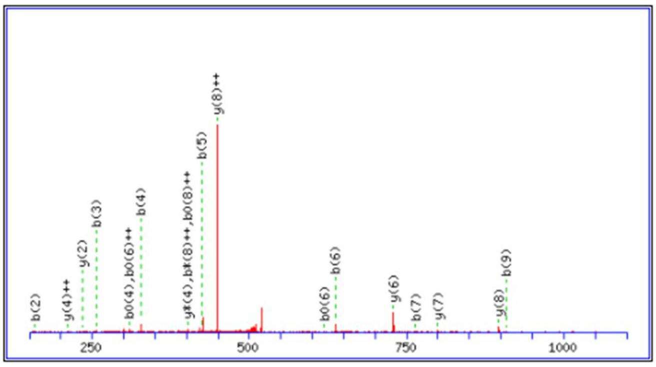

Monoisotopic mass of neutral peptide Mr(calc): 1053.5818
Variable modifications:
K6 : Acetoacetyl (K)
Ions Score: 28 **Expect:** 0.075
Matches : 19/96 fragment ions using 54 most intense peaks (help)

#	b	b^{++}	b^{*}	b^{*++}	b^{0}	b^{0++}	Seq.	y	y^{++}	y^{*}	y^{*++}	y^{0}	y^{0++}	#
1	88.0393	44.5233			70.0287	35.5180	S							10
2	***159.0764***	80.0418			141.0659	71.0366	A	967.5571	484.2822	950.5306	475.7689	949.5465	475.2769	9
3	***256.1292***	128.5682			238.1186	119.5629	P	**896.5200**	448.7636	879.4934	440.2504	878.5094	439.7584	8
4	***327.1663***	164.0868			309.1557	155.0815	A	**799.4672**	400.2373	782.4407	391.7240	781.4567	391.2320	7
5	***424.2191***	212.6132			406.2085	203.6079	P	**728.4301**	364.7187	711.4036	356.2054	710.4195	355.7134	6
6	***636.3352***	318.6712	619.3086	310.1579	618.3246	309.6659	K	631.3774	316.1923	614.3508	307.6790	613.3668	307.1870	5
7	***764.4301***	382.7187	747.4036	374.2054	746.4195	373.7134	K	419.2613	210.1343	402.2347	201.6210	401.2507	201.1290	4
8	821.4516	411.2294	804.4250	402.7162	803.4410	402.2241	G	291.1663	146.0868	274.1397	137.5735	273.1557	137.0815	3
9	***908.4836***	454.7454	891.4571	446.2322	890.4730	445.7402	S	**234.1448**	117.5761	217.1183	109.0628	216.1343	108.5708	2
10							K	147.1128	74.0600	130.0863	65.5468			1

H2BK16	KacacAVTKacAQK	Human HepG2 cells

MS/MS Fragmentation of KAVTKAQK
Found in O60814, Histone H2B type 1-K OS=Homo sapiens OX=9606 GN=HIST1H2BK PE=1 SV=3

Match to Query 4497: 998.576448 from(500.295500,2+)
Title: File:20201204_C12_HepG2_ingel_3_1.mzXML Scans:8281 RT:43.4923min Charge:2+ Fragmentation:cid
Data file 20201204_C12_HepG2_ingel_3_1.mgf

Click mouse within plot area to zoom in by factor of two about that point
Or, Plot from 100 to 1000 Da Full range
Label all possible matches ○ Label matches used for scoring ◉

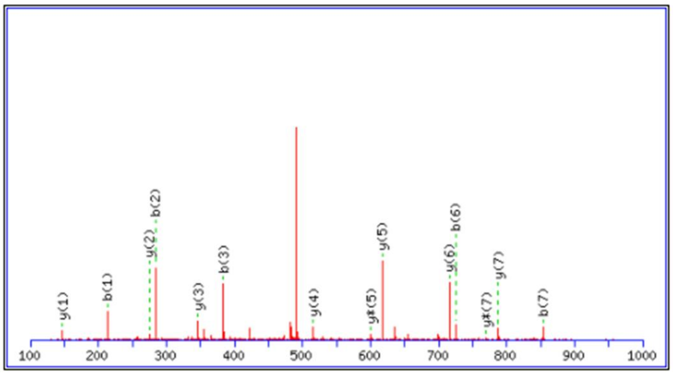

Monoisotopic mass of neutral peptide Mr(calc): 998.5760
Variable modifications:
K1 : Acetoacetyl (K)
K5 : Acetyl (K)
Ions Score: 54 **Expect:** 0.0022
Matches : 14/70 fragment ions using 26 most intense peaks (help)

#	b	b⁺⁺	b*	b*⁺⁺	b⁰	b⁰⁺⁺	Seq.	y	y⁺⁺	y*	y*⁺⁺	y⁰	y⁰⁺⁺	#
1	**213.1234**	107.0653	196.0968	98.5520			K							8
2	**284.1605**	142.5839	267.1339	134.0706			A	***787.4672***	394.2373	770.4407	385.7240	769.4567	385.2320	7
3	**383.2289**	192.1181	366.2023	183.6048			V	***716.4301***	358.7187	699.4036	350.2054	698.4196	349.7134	6
4	484.2766	242.6419	467.2500	234.1287	466.2660	233.6366	T	***617.3617***	309.1845	600.3352	300.6712	599.3511	300.1792	5
5	654.3821	327.6947	637.3556	319.1814	636.3715	318.6894	K	***516.3140***	258.6607	499.2875	250.1474			4
6	**725.4192**	363.2132	708.3927	354.7000	707.4087	354.2080	A	***346.2085***	173.6079	329.1819	165.0946			3
7	**853.4778**	427.2425	836.4512	418.7293	835.4672	418.2373	Q	***275.1714***	138.0893	258.1448	129.5761			2
8							K	***147.1128***	74.0600	130.0863	65.5468			1

H2BK34	**Kacac**ESYSVYVYK	Human HepG2 cells

MS/MS Fragmentation of **KESYSVYVYK**
Found in **O60814**, Histone H2B type 1-K OS=Homo sapiens OX=9606 GN=HIST1H2BK PE=1 SV=3

Match to Query 10687: 1348.652648 from(675.333600,2+)
Title: File:20201204_C12_HepG2_ingel_3_1.mzXML Scans:16947 RT:75.9022min Charge:2+ Fragmentation:cid
Data file 20201204_C12_HepG2_ingel_3_1.mgf

Click mouse within plot area to zoom in by factor of two about that point
Or, Plot from 100 to 1400 Da Full range
Label all possible matches ○ Label matches used for scoring ◉

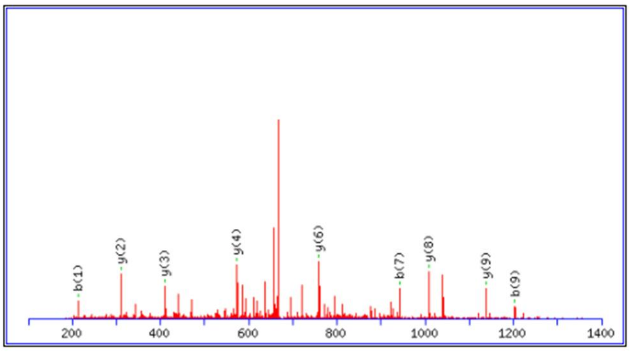

Monoisotopic mass of neutral peptide Mr(calc): 1348.6551
Variable modifications:
K1 : Acetoacetyl (K)
Ions Score: 48 **Expect:** 0.00033
Matches : 9/96 fragment ions using 12 most intense peaks (help)

#	b	b^{++}	b^{*}	b^{*++}	b^{0}	b^{0++}	Seq.	y	y^{++}	y^{*}	y^{*++}	y^{0}	y^{0++}	#
1	**213.1234**	107.0653	196.0968	98.5520			K							10
2	342.1660	171.5866	325.1394	163.0733	324.1554	162.5813	E	*1137.5463*	569.2768	1120.5197	560.7635	1119.5357	560.2715	9
3	429.1980	215.1026	412.1714	206.5894	411.1874	206.0973	S	*1008.5037*	504.7555	991.4771	496.2422	990.4931	495.7502	8
4	592.2613	296.6343	575.2348	288.1210	574.2508	287.6290	Y	921.4716	461.2395	904.4451	452.7262	903.4611	452.2342	7
5	679.2933	340.1503	662.2668	331.6370	661.2828	331.1450	S	*758.4083*	379.7078	741.3818	371.1945	740.3978	370.7025	6
6	778.3618	389.6845	761.3352	381.1712	760.3512	380.6792	V	671.3763	336.1918	654.3497	327.6785			5
7	**941.4251**	471.2162	924.3985	462.7029	923.4145	462.2109	Y	*572.3079*	286.6576	555.2813	278.1443			4
8	1040.4935	520.7504	1023.4670	512.2371	1022.4829	511.7451	V	*409.2445*	205.1259	392.2180	196.6126			3
9	**1203.5568**	602.2821	1186.5303	593.7688	1185.5463	593.2768	Y	*310.1761*	155.5917	293.1496	147.0784			2
10							K	147.1128	74.0600	130.0863	65.5468			1

H2BK46	VLKacacQVHPDTGISSK	Human HepG2 cells

MS/MS Fragmentation of VLKQVHPDTGISSK
Found in O60814, Histone H2B type 1-K OS=Homo sapiens OX=9606 GN=HIST1H2BK PE=1 SV=3

Match to Query 14322: 1591.854248 from(796.934400,2+)
Title: File:20201204_C12_HepG2_ingel_3_1.mzXML Scans:14058 RT:64.9465min Charge:2+ Fragmentation:cid
Data file 20201204_C12_HepG2_ingel_3_1.mgf

Click mouse within plot area to zoom in by factor of two about that point
Or, Plot from 200 to 1600 Da Full range
Label all possible matches ○ Label matches used for scoring ◉

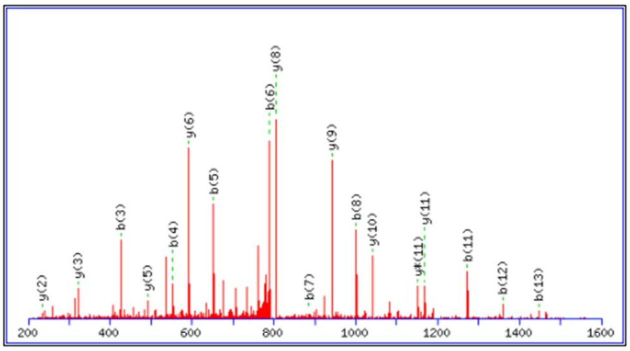

Monoisotopic mass of neutral peptide Mr(calc): 1591.8570
Variable modifications:
K3 : Acetoacetyl (K)
Ions Score: 49 **Expect:** 0.00026
Matches : 18/136 fragment ions using 42 most intense peaks (help)

#	b	b^{++}	b^{*}	b^{*++}	b^{0}	b^{0++}	Seq.	y	y^{++}	y^{*}	y^{*++}	y^{0}	y^{0++}	#
1	100.0757	50.5415					V							14
2	213.1598	107.0835					L	1493.7958	747.4016	1476.7693	738.8883	1475.7853	738.3963	13
3	*425.2758*	213.1416	408.2493	204.6283			K	1380.7118	690.8595	1363.6852	682.3462	1362.7012	681.8542	12
4	*553.3344*	277.1709	536.3079	268.6576			Q	*1168.5957*	584.8015	1151.5691	576.2882	1150.5851	575.7962	11
5	*652.4028*	326.7051	635.3763	318.1918			V	*1040.5371*	520.7722	1023.5106	512.2589	1022.5265	511.7669	10
6	*789.4617*	395.2345	772.4352	386.7212			H	*941.4687*	471.2380	924.4421	462.7247	923.4581	462.2327	9
7	*886.5145*	443.7609	869.4880	435.2476			P	*804.4098*	402.7085	787.3832	394.1953	786.3992	393.7032	8
8	*1001.5415*	501.2744	984.5149	492.7611	983.5309	492.2691	D	707.3570	354.1821	690.3305	345.6689	689.3464	345.1769	7
9	1102.5891	551.7982	1085.5626	543.2849	1084.5786	542.7929	T	*592.3301*	296.6687	575.3035	288.1554	574.3195	287.6634	6
10	1159.6106	580.3089	1142.5841	571.7957	1141.6000	571.3037	G	*491.2824*	246.1448	474.2558	237.6316	473.2718	237.1395	5
11	*1272.6947*	636.8510	1255.6681	628.3377	1254.6841	627.8457	I	434.2609	217.6341	417.2344	209.1208	416.2504	208.6288	4
12	*1359.7267*	680.3670	1342.7001	671.8537	1341.7161	671.3617	S	*321.1769*	161.0921	304.1503	152.5788	303.1663	152.0868	3
13	*1446.7587*	723.8830	1429.7322	715.3697	1428.7482	714.8777	S	*234.1448*	117.5761	217.1183	109.0628	216.1343	108.5708	2
14							K	147.1128	74.0600	130.0863	65.5468			1

H2BK86	LAHYSKacacR	Human HepG2 cells

MS/MS Fragmentation of LAHYSKR
Found in Q96A08, Histone H2B type 1-A OS=Homo sapiens OX=9606 GN=HIST1H2BA PE=1 SV=3

Match to Query 627: 957.502048 from(479.758300,2+)
Title: File:20210213_HepG2_Kaa_C12.mzXML Scans:1520 RT:16.9090min Charge:2+ Fragmentation:cid
Data file 20210213_HepG2_Kaa_C12.mgf

Click mouse within plot area to zoom in by factor of two about that point
Or, Plot from 100 to 850 Da Full range
Label all possible matches ○ Label matches used for scoring ◉

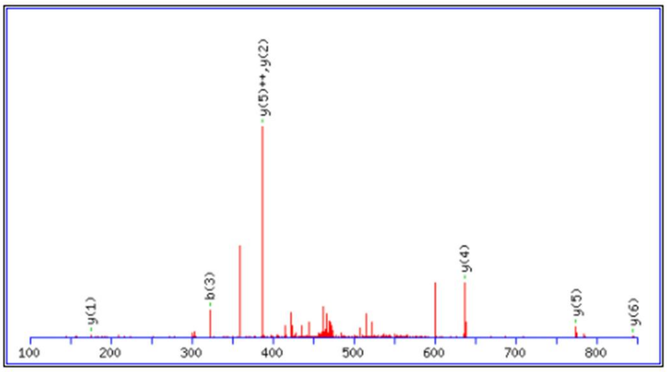

Monoisotopic mass of neutral peptide Mr(calc): 957.5032
Variable modifications:
K6 : Acetoacetyl (K)
Ions Score: 37 **Expect:** 0.031
Matches : 7/50 fragment ions using 8 most intense peaks (help)

#	b	b^{++}	b^*	b^{*++}	b^0	b^{0++}	Seq.	y	y^{++}	y^*	y^{*++}	y^0	y^{0++}	#
1	114.0913	57.5493					L							7
2	185.1285	93.0679					A	845.4264	423.2169	828.3999	414.7036	827.4159	414.2116	6
3	322.1874	161.5973					H	774.3893	387.6983	757.3628	379.1850	756.3787	378.6930	5
4	485.2507	243.1290					Y	637.3304	319.1688	620.3039	310.6556	619.3198	310.1636	4
5	572.2827	286.6450			554.2722	277.6397	S	474.2671	237.6372	457.2405	229.1239	456.2565	228.6319	3
6	784.3988	392.7030	767.3723	384.1898	766.3882	383.6978	K	387.2350	194.1212	370.2085	185.6079			2
7							R	175.1190	88.0631	158.0924	79.5498			1

\

**H2BK116**	**HAVSEGTKacacAVTKacacYTSSKpr**	**Rat liver**

MS/MS Fragmentation of HAVSEGTKAVTKYTSSK
Found in M0R4L7, Histone H2B OS=Rattus norvegicus OX=10116 GN=Hist1h2bl PE=3 SV=1

Match to Query 11836: 2017.023248 from(1009.518900,2+)
Title: File:20201016_gel_2_global.mzXML Scans:12812 RT:72.3862min Charge:2+ Fragmentation:cid
Data file 20201016_gel_2_global.mgf

Click mouse within plot area to zoom in by factor of two about that point
Or, Plot from 200 to 2000 Da Full range
Label all possible matches ○ Label matches used for scoring ●

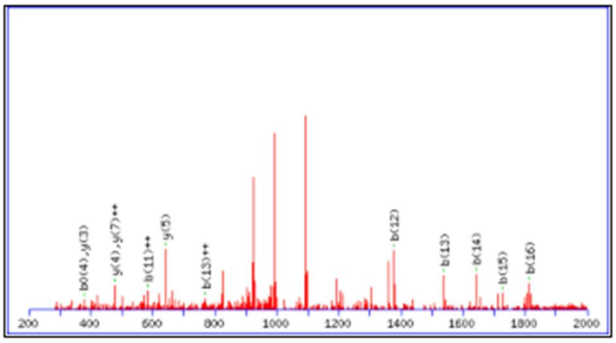

Monoisotopic mass of neutral peptide Mr(calc): 2017.0004
Variable modifications:
K8 : Acetoacetyl (K)
K12 : Acetoacetyl (K)
K17 : Propionyl (K)
Ions Score: 28 **Expect:** 0.04
Matches : 12/170 fragment ions using 18 most intense peaks (help)

#	b	b^{++}	b^{*}	b^{*++}	b^{0}	b^{0++}	Seq.	y	y^{++}	y^{*}	y^{*++}	y^{0}	y^{0++}	#
1	138.0662	69.5367					H							17
2	209.1033	105.0553					A	1880.9488	940.9780	1863.9222	932.4647	1862.9382	931.9727	16
3	308.1717	154.5895					V	1809.9117	905.4595	1792.8851	896.9462	1791.9011	896.4542	15
4	395.2037	198.1055			377.1932	189.1002	S	1710.8432	855.9253	1693.8167	847.4120	1692.8327	846.9200	14
5	524.2463	262.6268			506.2358	253.6215	E	1623.8112	812.4092	1606.7847	803.8960	1605.8006	803.4040	13
6	581.2678	291.1375			563.2572	282.1323	G	1494.7686	747.8879	1477.7421	739.3747	1476.7581	738.8827	12
7	682.3155	341.6614			664.3049	332.6561	T	1437.7472	719.3772	1420.7206	710.8639	1419.7366	710.3719	11
8	894.4316	447.7194	877.4050	439.2061	876.4210	438.7141	K	1336.6995	668.8534	1319.6729	660.3401	1318.6889	659.8481	10
9	965.4687	483.2380	948.4421	474.7247	947.4581	474.2327	A	1124.5834	562.7953	1107.5568	554.2821	1106.5728	553.7900	9
10	1064.5371	532.7722	1047.5106	524.2589	1046.5265	523.7669	V	1053.5463	527.2768	1036.5197	518.7635	1035.5357	518.2715	8
11	1165.5848	**583.2960**	1148.5582	574.7828	1147.5742	574.2907	T	954.4779	477.7426	937.4513	469.2293	936.4673	468.7373	7
12	***1377.7009***	689.3541	1360.6743	680.8408	1359.6903	680.3488	K	853.4302	427.2187	836.4036	418.7055	835.4196	418.2134	6
13	***1540.7642***	**770.8857**	1523.7377	762.3725	1522.7536	761.8805	Y	**641.3141**	321.1607	624.2875	312.6474	623.3035	312.1554	5
14	***1641.8119***	821.4096	1624.7853	812.8963	1623.8013	812.4043	T	**478.2508**	239.6290	461.2242	231.1157	460.2402	230.6237	4
15	***1728.8439***	864.9256	1711.8174	856.4123	1710.8333	855.9203	S	**377.2031**	189.1052	360.1765	180.5919	359.1925	180.0999	3
16	***1815.8759***	908.4416	1798.8494	899.9283	1797.8654	899.4363	S	290.1710	145.5892	273.1445	137.0759	272.1605	136.5839	2
17							K	203.1390	102.0731	186.1125	93.5599			1

H2BK120	HAVSEGTKacacAVTKacacYTSSKpr	Rat liver

MS/MS Fragmentation of HAVSEGTKAVTKYTSSK
Found in M0R4L7, Histone H2B OS=Rattus norvegicus OX=10116 GN=Hist1h2bl PE=3 SV=1

Match to Query 11836: 2017.023248 from(1009.518900,2+)
Title: File:20201016_gel_2_global.mzXML Scans:12812 RT:72.3862min Charge:2+ Fragmentation:cid
Data file 20201016_gel_2_global.mgf

Click mouse within plot area to zoom in by factor of two about that point
Or, Plot from 200 to 2000 Da Full range
Label all possible matches ○ Label matches used for scoring ◉

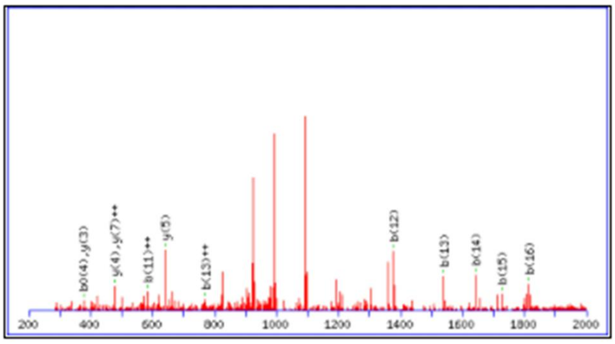

Monoisotopic mass of neutral peptide Mr(calc): 2017.0004
Variable modifications:
K8 : Acetoacetyl (K)
K12 : Acetoacetyl (K)
K17 : Propionyl (K)
Ions Score: 28 **Expect:** 0.04
Matches : 12/170 fragment ions using 18 most intense peaks (help)

#	b	b^{++}	b^{*}	b^{*++}	b^{0}	b^{0++}	Seq.	y	y^{++}	y^{*}	y^{*++}	y^{0}	y^{0++}	#
1	138.0662	69.5367					H							17
2	209.1033	105.0553					A	1880.9488	940.9780	1863.9222	932.4647	1862.9382	931.9727	16
3	308.1717	154.5895					V	1809.9117	905.4595	1792.8851	896.9462	1791.9011	896.4542	15
4	395.2037	198.1055			377.1932	189.1002	S	1710.8432	855.9253	1693.8167	847.4120	1692.8327	846.9200	14
5	524.2463	262.6268			506.2358	253.6215	E	1623.8112	812.4092	1606.7847	803.8960	1605.8006	803.4040	13
6	581.2678	291.1375			563.2572	282.1323	G	1494.7686	747.8879	1477.7421	739.3747	1476.7581	738.8827	12
7	682.3155	341.6614			664.3049	332.6561	T	1437.7472	719.3772	1420.7206	710.8639	1419.7366	710.3719	11
8	894.4316	447.7194	877.4050	439.2061	876.4210	438.7141	K	1336.6995	668.8534	1319.6729	660.3401	1318.6889	659.8481	10
9	965.4687	483.2380	948.4421	474.7247	947.4581	474.2327	A	1124.5834	562.7953	1107.5568	554.2821	1106.5728	553.7900	9
10	1064.5371	532.7722	1047.5106	524.2589	1046.5265	523.7669	V	1053.5463	527.2768	1036.5197	518.7635	1035.5357	518.2715	8
11	1165.5848	**583.2960**	1148.5582	574.7828	1147.5742	574.2907	T	954.4779	477.7426	937.4513	469.2293	936.4673	468.7373	7
12	***1377.7009***	689.3541	1360.6743	680.8408	1359.6903	680.3488	K	853.4302	427.2187	836.4036	418.7055	835.4196	418.2134	6
13	***1540.7642***	**770.8857**	1523.7377	762.3725	1522.7536	761.8805	Y	**641.3141**	321.1607	624.2875	312.6474	623.3035	312.1554	5
14	***1641.8119***	821.4096	1624.7853	812.8963	1623.8013	812.4043	T	**478.2508**	239.6290	461.2242	231.1157	460.2402	230.6237	4
15	***1728.8439***	864.9256	1711.8174	856.4123	1710.8333	855.9203	S	**377.2031**	189.1052	360.1765	180.5919	359.1925	180.0999	3
16	***1815.8759***	908.4416	1798.8494	899.9283	1797.8654	899.4363	S	290.1710	145.5892	273.1445	137.0759	272.1605	136.5839	2
17							K	203.1390	102.0731	186.1125	93.5599			1

H3K9	KacacSTGGKprAPR	Human HepG2 cells

MS/MS Fragmentation of KSTGGKAPR
Found in Q71DI3, Histone H3.2 OS=Homo sapiens OX=9606 GN=HIST2H3A PE=1 SV=3

Match to Query 4270: 1040.554448 from(521.284500,2+)
Title: File:20201204_C12_HepG2_ingel_1_1.mzXML Scans:6956 RT:41.6342min Charge:2+ Fragmentation:cid
Data file 20201204_C12_HepG2_ingel_1_1.mgf

Click mouse within plot area to zoom in by factor of two about that point
Or, Plot from 100 to 1050 Da Full range
Label all possible matches ○ Label matches used for scoring ◉

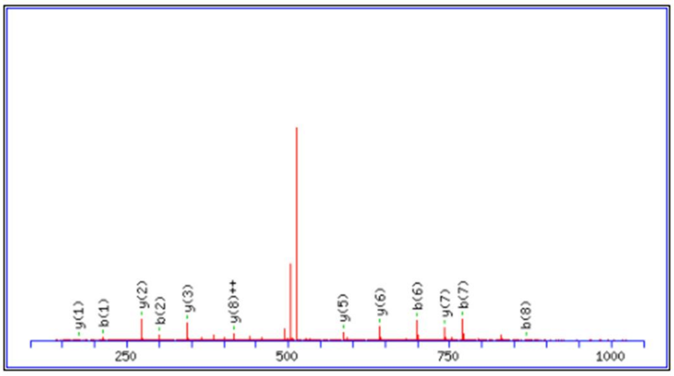

Monoisotopic mass of neutral peptide Mr(calc): 1040.5614
Variable modifications:
K1 : Acetoacetyl (K)
K6 : Propionyl (K)
Ions Score: 51 **Expect:** 0.003
Matches : 12/82 fragment ions using 18 most intense peaks (help)

#	b	b^{++}	b^{*}	b^{*++}	b^{0}	b^{0++}	Seq.	y	y^{++}	y^{*}	y^{*++}	y^{0}	y^{0++}	#
1	213.1234	107.0653	196.0968	98.5520			K							9
2	300.1554	150.5813	283.1288	142.0681	282.1448	141.5761	S	829.4526	415.2300	812.4261	406.7167	811.4421	406.2247	8
3	401.2031	201.1052	384.1765	192.5919	383.1925	192.0999	T	742.4206	371.7139	725.3941	363.2007	724.4100	362.7087	7
4	458.2245	229.6159	441.1980	221.1026	440.2140	220.6106	G	641.3729	321.1901	624.3464	312.6768			6
5	515.2460	258.1266	498.2195	249.6134	497.2354	249.1214	G	584.3515	292.6794	567.3249	284.1661			5
6	699.3672	350.1872	682.3406	341.6740	681.3566	341.1819	K	527.3300	264.1686	510.3035	255.6554			4
7	770.4043	385.7058	753.3777	377.1925	752.3937	376.7005	A	343.2088	172.1081	326.1823	163.5948			3
8	867.4571	434.2322	850.4305	425.7189	849.4465	425.2269	P	272.1717	136.5895	255.1452	128.0762			2
9							R	175.1190	88.0631	158.0924	79.5498			1

H3K14	KmeSTGGKacacAPR	Human HepG2 cells

MS/MS Fragmentation of KSTGGKAPR
Found in Q71DI3, Histone H3.2 OS=Homo sapiens OX=9606 GN=HIST2H3A PE=1 SV=3

Match to Query 4481: 998.538648 from(500.276600,2+)
Title: File:20201204_C12_HepG2_ingel_3_2.mzXML Scans:5821 RT:33.6292min Charge:2+ Fragmentation:cid
Data file 20201204_C12_HepG2_ingel_3_2.mgf

Click mouse within plot area to zoom in by factor of two about that point
Or, Plot from 100 to 1000 Da Full range
Label all possible matches ○ Label matches used for scoring ◉

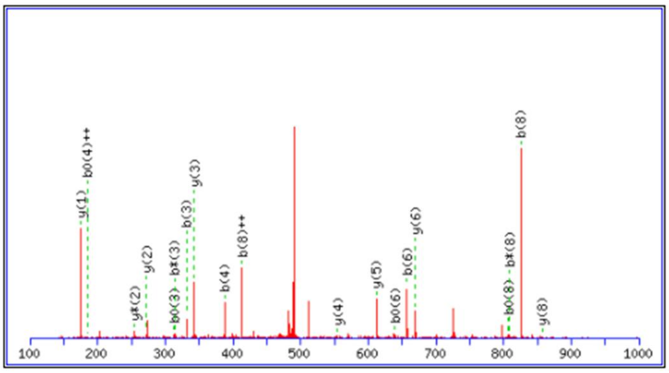

Monoisotopic mass of neutral peptide Mr(calc): 998.5509
Variable modifications:
K1 : Methyl (KR)
K6 : Acetoacetyl (K)
Ions Score: 33 **Expect:** 0.13
Matches : 19/82 fragment ions using 57 most intense peaks (help)

#	b	b^{++}	b^{*}	b^{*++}	b^{0}	b^{0++}	Seq.	y	y^{++}	y^{*}	y^{*++}	y^{0}	y^{0++}	#
1	143.1179	72.0626	126.0913	63.5493			K							9
2	230.1499	115.5786	213.1234	107.0653	212.1394	106.5733	S	*857.4476*	429.2274	840.4210	420.7141	839.4370	420.2221	8
3	**331.1976**	166.1024	314.1710	157.5892	313.1870	157.0972	T	770.4155	385.7114	753.3890	377.1981	752.4050	376.7061	7
4	**388.2191**	194.6132	371.1925	186.0999	370.2085	185.6079	G	*669.3679*	335.1876	652.3413	326.6743			6
5	445.2405	223.1239	428.2140	214.6106	427.2300	214.1186	G	*612.3464*	306.6768	595.3198	298.1636			5
6	**657.3566**	329.1819	640.3301	320.6687	639.3461	320.1767	K	*555.3249*	278.1661	538.2984	269.6528			4
7	728.3937	364.7005	711.3672	356.1872	710.3832	355.6952	A	*343.2088*	172.1081	326.1823	163.5948			3
8	**825.4465**	413.2269	808.4199	404.7136	807.4359	404.2216	P	*272.1717*	136.5895	255.1452	128.0762			2
9							R	*175.1190*	88.0631	158.0924	79.5498			1

H3K18	KacacQLATKprAAR	Human HepG2 cells

MS/MS Fragmentation of KQLATKAAR
Found in Q71DI3, Histone H3.2 OS=Homo sapiens OX=9606 GN=HIST2H3A PE=1 SV=3

Match to Query 5435: 1125.648848 from(563.831700,2+)
Title: File:20201204_C12_HepG2_ingel_1_1.mzXML Scans:13639 RT:65.6817min Charge:2+ Fragmentation:cid
Data file 20201204_C12_HepG2_ingel_1_1.mgf

Click mouse within plot area to zoom in by factor of two about that point
Or, Plot from 150 to 1150 Da Full range
Label all possible matches ○ Label matches used for scoring ◉

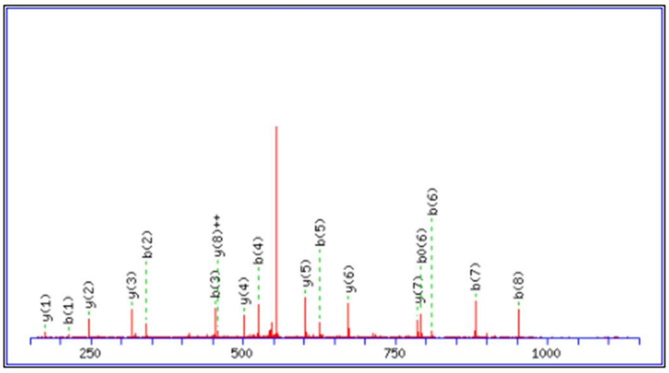

Monoisotopic mass of neutral peptide Mr(calc): 1125.6506
Variable modifications:
K1 : Acetoacetyl (K)
K6 : Propionyl (K)
Ions Score: 57 **Expect:** 0.00028
Matches : 17/80 fragment ions using 30 most intense peaks (help)

#	b	b^{++}	b^{*}	b^{*++}	b^{0}	b^{0++}	Seq.	y	y^{++}	y^{*}	y^{*++}	y^{0}	y^{0++}	#
1	*213.1234*	107.0653	196.0968	98.5520			K							9
2	*341.1819*	171.0946	324.1554	162.5813			Q	914.5418	457.7745	897.5152	449.2613	896.5312	448.7693	8
3	*454.2660*	227.6366	437.2395	219.1234			L	**786.4832**	393.7452	769.4567	385.2320	768.4727	384.7400	7
4	*525.3031*	263.1552	508.2766	254.6419			A	**673.3992**	337.2032	656.3726	328.6899	655.3886	328.1979	6
5	*626.3508*	313.6790	609.3243	305.1658	608.3402	304.6738	T	**602.3620**	301.6847	585.3355	293.1714	584.3515	292.6794	5
6	*810.4720*	405.7396	793.4454	397.2264	792.4614	396.7343	K	**501.3144**	251.1608	484.2878	242.6475			4
7	*881.5091*	441.2582	864.4825	432.7449	863.4985	432.2529	A	**317.1932**	159.1002	300.1666	150.5870			3
8	*952.5462*	476.7767	935.5197	468.2635	934.5356	467.7715	A	**246.1561**	123.5817	229.1295	115.0684			2
9							R	**175.1190**	88.0631	158.0924	79.5498			1

H3K23	KprQLATKacacAARme	Human HepG2 cells

MS/MS Fragmentation of **KQLATKAAR**
Found in **Q71DI3**, Histone H3.2 OS=Homo sapiens OX=9606 GN=HIST2H3A PE=1 SV=3

Match to Query 5618: 1139.659248 from(570.836900,2+)
Title: File:20201204_C12_HepG2_ingel_1_1.mzXML Scans:9252 RT:49.9000min Charge:2+ Fragmentation:cid
Data file 20201204_C12_HepG2_ingel_1_1.mgf

Click mouse within plot area to zoom in by factor of two about that point
Or, Plot from 150 to 1150 Da Full range
Label all possible matches ○ Label matches used for scoring ◉

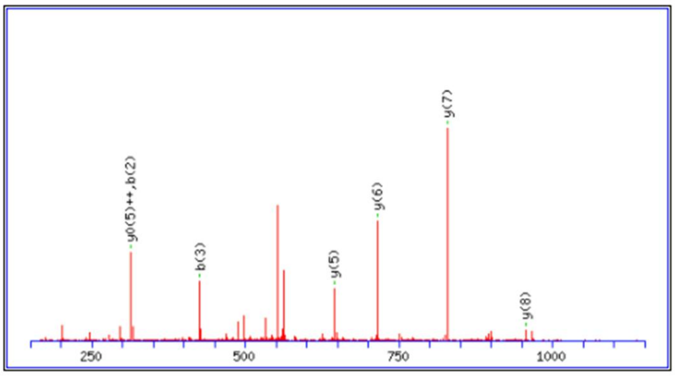

Monoisotopic mass of neutral peptide Mr(calc): 1139.6662
Variable modifications:
K1 : Propionyl (K)
K6 : Acetoacetyl (K)
R9 : Methyl (KR)
Ions Score: 27 **Expect:** 0.45
Matches : 7/80 fragment ions using 10 most intense peaks (help)

#	b	b^{++}	b^{*}	b^{*++}	b^{0}	b^{0++}	Seq.	y	y^{++}	y^{*}	y^{*++}	y^{0}	y^{0++}	#
1	185.1285	93.0679	168.1019	84.5546			K							9
2	**313.1870**	157.0972	296.1605	148.5839			Q	***956.5524***	478.7798	939.5258	470.2665	938.5418	469.7745	8
3	**426.2711**	213.6392	409.2445	205.1259			L	***828.4938***	414.7505	811.4672	406.2373	810.4832	405.7452	7
4	497.3082	249.1577	480.2817	240.6445			A	***715.4097***	358.2085	698.3832	349.6952	697.3992	349.2032	6
5	598.3559	299.6816	581.3293	291.1683	580.3453	290.6763	T	***644.3726***	322.6899	627.3461	314.1767	626.3620	313.6847	5
6	810.4720	405.7396	793.4454	397.2264	792.4614	396.7343	K	543.3249	272.1661	526.2984	263.6528			4
7	881.5091	441.2582	864.4825	432.7449	863.4985	432.2529	A	331.2088	166.1081	314.1823	157.5948			3
8	952.5462	476.7767	935.5197	468.2635	934.5356	467.7715	A	260.1717	130.5895	243.1452	122.0762			2
9							R	189.1346	95.0709	172.1081	86.5577			1

H3K27	KacacSAPATGGVKprKprPHR	Human HepG2 cells

MS/MS Fragmentation of KSAPATGGVKKPHR
Found in Q71DI3, Histone H3.2 OS=Homo sapiens OX=9606 GN=HIST2H3A PE=1 SV=3

Match to Query 12563: 1628.893048 from(815.453800,2+)
Title: File:20201204_C12_HepG2_ingel_1_1.mzXML Scans:10383 RT:54.0352min Charge:2+ Fragmentation:cid
Data file 20201204_C12_HepG2_ingel_1_1.mgf

Click mouse within plot area to zoom in by factor of two about that point
Or, Plot from 200 to 1700 Da Full range
Label all possible matches ○ Label matches used for scoring ◉

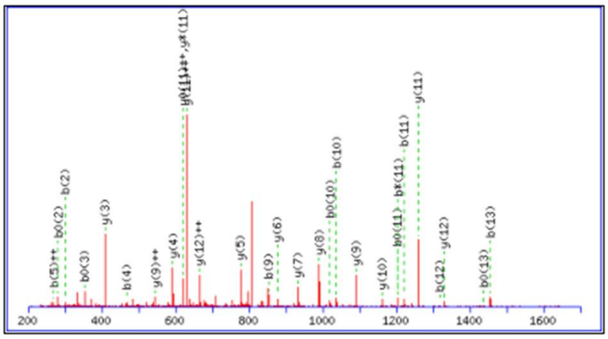

Monoisotopic mass of neutral peptide Mr(calc): 1628.8998
Variable modifications:
K1 : Acetoacetyl (K)
K10 : Propionyl (K)
K11 : Propionyl (K)
Ions Score: 58 **Expect:** 0.0018
Matches : 29/188 fragment ions using 57 most intense peaks (help)

#	b	b^{++}	b^*	b^{*++}	b^0	b^{0++}	Seq	y	y^{++}	y^*	y^{*++}	y^0	y^{0++}	#
1	213.1234	107.0653	196.0968	98.5520			K							14
2	**300.1554**	150.5813	283.1288	142.0681	282.1448	141.5761	S	1417.7910	709.3992	1400.7645	700.8859	1399.7805	700.3939	13
3	371.1925	186.0999	354.1660	177.5866	353.1819	177.0946	A	***1330.7590***	665.8831	1313.7324	657.3699	1312.7484	656.8779	12
4	**468.2453**	234.6263	451.2187	226.1130	450.2347	225.6210	P	***1259.7219***	630.3646	1242.6953	621.8513	1241.7113	621.3593	11
5	539.2824	270.1448	522.2558	261.6316	521.2718	261.1395	A	***1162.6691***	581.8382	1145.6426	573.3249	1144.6586	572.8329	10
6	640.3301	320.6687	623.3035	312.1554	622.3195	311.6634	T	***1091.6320***	546.3196	1074.6055	537.8064	1073.6214	537.3144	9
7	697.3515	349.1794	680.3250	340.6661	679.3410	340.1741	G	***990.5843***	495.7958	973.5578	487.2825			8
8	754.3730	377.6901	737.3464	369.1769	736.3624	368.6849	G	***933.5629***	467.2851	916.5363	458.7718			7
9	**853.4414**	427.2243	836.4149	418.7111	835.4308	418.2191	V	***876.5414***	438.7743	859.5148	430.2611			6
10	**1037.5626**	519.2849	1020.5360	510.7717	1019.5520	510.2796	K	***777.4730***	389.2401	760.4464	380.7269			5
11	**1221.6838**	611.3455	1204.6572	602.8322	1203.6732	602.3402	K	***593.3518***	297.1795	576.3253	288.6663			4
12	**1318.7365**	659.8719	1301.7100	651.3586	1300.7260	650.8666	P	***409.2306***	205.1190	392.2041	196.6057			3
13	**1455.7954**	728.4014	1438.7689	719.8881	1437.7849	719.3961	H	312.1779	156.5926	295.1513	148.0793			2
14							R	175.1190	88.0631	158.0924	79.5498			1

H3K36	KacacSAPATGGVKacacKacPHR	Human MCF-7cell

MS/MS Fragmentation of **KSAPATGGVKKPHR**
Found in **Q71DI3**, Histone H3.2
Match to Query 13110: 1624.988172 from(542.670000,3+)

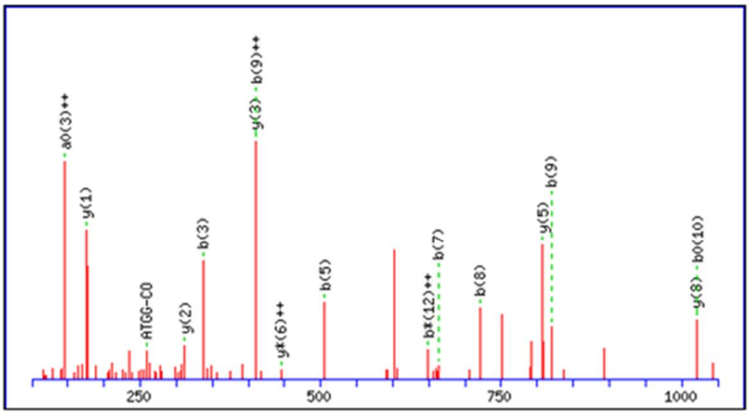

Monoisotopic mass of neutral peptide Mr(calc): 1624.98
Variable modifications:
K1 : Trimethyl (K8)
K10 : Acetoacetyl (K8)
K11 : Trimethyl (K8)
Ions Score: 49 **Expect:** 0.022
Matches (Bold Red): 16/319 fragment ions using 20 most intense peaks

#	Immon.	a	a^{++}	a^*	a^{*++}	a^0	a^{0++}	b	b^{++}	b^*	b^{*++}	b^0	b^{0++}	Seq.	y	y^{++}	y^*	y^{*++}	y^0	y^{0++}	#
1	151.17	151.17	76.09	134.14	67.57			179.16	90.09	162.14	81.57			K							14
2	60.04	238.20	119.60	221.17	111.09	220.19	110.60	266.20	133.60	249.17	125.09	248.18	124.60	S	1447.84	724.42	1430.81	715.91	1429.82	715.42	13
3	44.05	309.24	155.12	292.21	146.61	291.23	**146.12**	**337.23**	169.12	320.21	160.61	319.22	160.11	A	1360.80	680.91	1343.78	672.39	1342.79	671.90	12
4	70.07	406.29	203.65	389.26	195.14	388.28	194.64	434.29	217.65	417.26	209.13	416.27	208.64	P	1289.77	645.39	1272.74	636.87	1271.76	636.38	11
5	44.05	477.33	239.17	460.30	230.65	459.32	230.16	**505.32**	253.16	488.30	244.65	487.31	244.16	A	1192.71	596.86	1175.69	588.35	1174.70	587.85	10
6	74.06	578.38	289.69	561.35	281.18	560.36	280.69	606.37	303.69	589.34	295.18	588.36	294.68	T	1121.68	561.34	1104.65	552.83	1103.67	552.34	9
7	30.03	635.40	318.20	618.37	309.69	617.39	309.20	**663.39**	332.20	646.36	323.69	645.38	323.19	G	**1020.63**	510.82	1003.60	502.30			8
8	30.03	692.42	346.71	675.39	338.20	674.41	337.71	**720.41**	360.71	703.39	352.20	702.40	351.70	G	963.61	482.31	946.58	473.79			7
9	72.08	791.49	396.25	774.46	387.73	773.48	387.24	**819.48**	**410.24**	802.45	401.73	801.47	401.24	V	906.59	453.80	889.56	**445.28**			6
10	193.14	1011.62	506.31	994.59	497.80	993.61	497.31	1039.61	520.31	1022.59	511.80	**1021.60**	511.30	K	**807.52**	404.26	790.49	395.75			5
11	151.17	1189.77	595.39	1172.75	586.88	1171.76	586.38	1217.77	609.39	1200.74	600.87	1199.76	600.38	K	587.39	294.20	570.36	285.68			4
12	70.07	1286.83	643.92	1269.80	635.40	1268.82	634.91	1314.82	657.91	1297.79	**649.40**	1296.81	648.91	P	**409.23**	205.12	392.20	196.61			3
13	110.07	1423.88	712.45	1406.86	703.93	1405.87	703.44	1451.88	726.44	1434.85	717.93	1433.87	717.44	H	**312.18**	156.59	295.15	148.08			2
14	129.11													R	**175.12**	88.06	158.09	79.55			1

H3K37	KacacSAPATGGVKacKacacPHR	Human HepG2 cells

MS/MS Fragmentation of **KSAPATGGVKKPHR**
Found in **Q71DI3**, Histone H3.2 OS=Homo sapiens OX=9606 GN=HIST2H3A PE=1 SV=3

Match to Query 12271: 1642.910648 from(822.462600,2+)
Title: File:20201204_C12_HepG2_ingel_1_2.mzXML Scans:11664 RT:58.1875min Charge:2+ Fragmentation:cid
Data file 20201204_C12_HepG2_ingel_1_2.mgf

Click mouse within plot area to zoom in by factor of two about that point
Or, Plot from 200 to 1700 Da Full range
Label all possible matches ○ Label matches used for scoring ◉

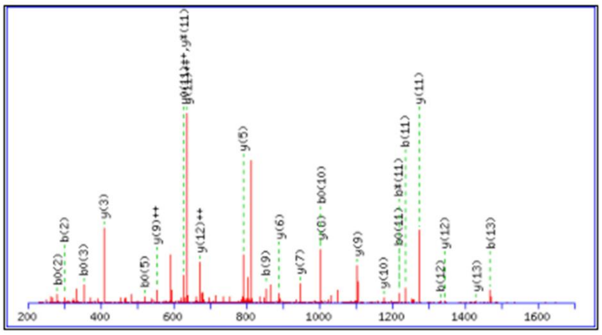

Monoisotopic mass of neutral peptide Mr(calc): 1642.8791
Variable modifications:
K1 : Acetoacetyl (K)
K10 : Acetyl (K)
K11 : Acetoacetyl (K)
Ions Score: 72 **Expect:** 1.8e-008
Matches : 28/188 fragment ions using 41 most intense peaks (help)

#	b	b^{++}	b^{*}	b^{*++}	b^{0}	b^{0++}	Seq	y	y^{++}	y^{*}	y^{*++}	y^{0}	y^{0++}	#
1	213.1234	107.0653	196.0968	98.5520			K							14
2	**300.1554**	150.5813	283.1288	142.0681	282.1448	141.5761	S	***1431.7703***	716.3888	1414.7437	707.8755	1413.7597	707.3835	13
3	371.1925	186.0999	354.1660	177.5866	353.1819	177.0946	A	***1344.7383***	672.8728	1327.7117	664.3595	1326.7277	663.8675	12
4	468.2453	234.6263	451.2187	226.1130	450.2347	225.6210	P	***1273.7011***	637.3542	1256.6746	628.8409	1255.6906	628.3489	11
5	539.2824	270.1448	522.2558	261.6316	521.2718	261.1395	A	***1176.6484***	588.8278	1159.6218	580.3146	1158.6378	579.8225	10
6	640.3301	320.6687	623.3035	312.1554	622.3195	311.6634	T	***1105.6113***	553.3093	1088.5847	544.7960	1087.6007	544.3040	9
7	697.3515	349.1794	680.3250	340.6661	679.3410	340.1741	G	***1004.5636***	502.7854	987.5370	494.2722			8
8	754.3730	377.6901	737.3464	369.1769	736.3624	368.6849	G	***947.5421***	474.2747	930.5156	465.7614			7
9	**853.4414**	427.2243	836.4149	418.7111	835.4308	418.2191	V	***890.5207***	445.7640	873.4941	437.2507			6
10	1023.5469	512.2771	1006.5204	503.7638	1005.5364	503.2718	K	***791.4522***	396.2298	774.4257	387.7165			5
11	**1235.6630**	618.3352	1218.6365	609.8219	1217.6525	609.3299	K	621.3467	311.1770	604.3202	302.6637			4
12	**1332.7158**	666.8615	1315.6892	658.3483	1314.7052	657.8563	P	***409.2306***	205.1190	392.2041	196.6057			3
13	**1469.7747**	735.3910	1452.7482	726.8777	1451.7641	726.3857	H	312.1779	156.5926	295.1513	148.0793			2
14							R	175.1190	88.0631	158.0924	79.5498			1

H3K56	RmeYQKacacSTELLIR	Human HepG2 cells

MS/MS Fragmentation of **RYQKSTELLIR**
Found in **Q71DI3**, Histone H3.2 OS=Homo sapiens OX=9606 GN=HIST2H3A PE=1 SV=3

Match to Query 10875: 1503.831448 from(752.923000,2+)
Title: File:20201204_C12_HepG2_ingel_1_1.mzXML Scans:14283 RT:67.9427min Charge:2+ Fragmentation:cid
Data file 20201204_C12_HepG2_ingel_1_1.mgf

Click mouse within plot area to zoom in by factor of two about that point
Or, Plot from 200 to 1600 Da Full range
Label all possible matches ○ Label matches used for scoring ◉

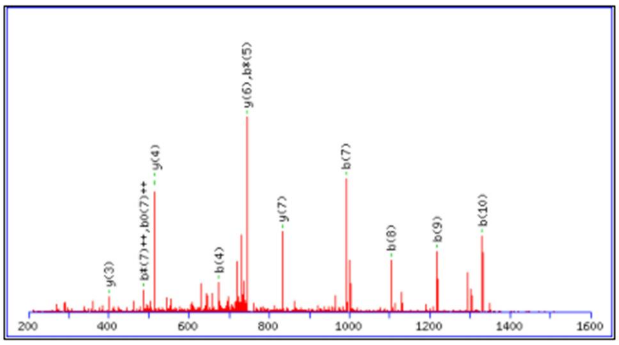

Monoisotopic mass of neutral peptide Mr(calc): 1503.8409
Variable modifications:
R1 : Methyl (KR)
K4 : Acetoacetyl (K)
Ions Score: 34 **Expect:** 0.058
Matches : 12/104 fragment ions using 13 most intense peaks (help)

#	b	b++	b*	b*++	b0	b0++	Seq	y	y++	y*	y*++	y0	y0++	#
1	171.1240	86.0657	154.0975	77.5524			R							11
2	334.1874	167.5973	317.1608	159.0840			Y	1334.7314	667.8694	1317.7049	659.3561	1316.7209	658.8641	10
3	462.2459	231.6266	445.2194	223.1133			Q	1171.6681	586.3377	1154.6416	577.8244	1153.6575	577.3324	9
4	***674.3620***	337.6847	657.3355	329.1714			K	1043.6095	522.3084	1026.5830	513.7951	1025.5990	513.3031	8
5	761.3941	381.2007	744.3675	372.6874	743.3835	372.1954	S	**831.4934**	416.2504	814.4669	407.7371	813.4829	407.2451	7
6	862.4417	431.7245	845.4152	423.2112	844.4312	422.7192	T	**744.4614**	372.7343	727.4349	364.2211	726.4509	363.7291	6
7	***991.4843***	496.2458	974.4578	487.7325	973.4738	487.2405	E	643.4137	322.2105	626.3872	313.6972	625.4032	313.2052	5
8	***1104.5684***	552.7878	1087.5419	544.2746	1086.5578	543.7826	L	**514.3711**	257.6892	497.3446	249.1759			4
9	***1217.6525***	609.3299	1200.6259	600.8166	1199.6419	600.3246	L	**401.2871**	201.1472	384.2605	192.6339			3
10	***1330.7365***	665.8719	1313.7100	657.3586	1312.7260	656.8666	I	288.2030	144.6051	271.1765	136.0919			2
11							R	175.1190	88.0631	158.0924	79.5498			1

H3K79	EIAQDFKacacTDLR	Human MCF-7 cells

MS/MS Fragmentation of **EIAQDFKTDLR**
Found in **Q71DI3**, Histone H3.2
Match to Query 5262: 1418.703318 from(710.358935,2+)

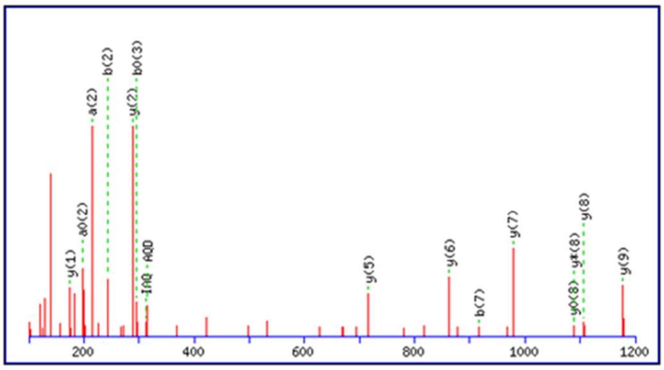

Monoisotopic mass of neutral peptide Mr(calc): 1418.70
Variable modifications:
K7 : Acetoacetyl (K)
Ions Score: 38 **Expect:** 0.12
Matches (Bold Red): 16/226 fragment ions using 30 most intense peaks

#	Immon.	a	a++	a*	a*++	a0	a0++	b	b++	b*	b*++	b0	b0++	Seq.	y	y++	y*	y*++	y0	y0++	#
1	102.05	102.05	51.53			84.04	42.53	130.05	65.53			112.04	56.52	E							11
2	86.10	**215.14**	108.07			**197.13**	99.07	**243.13**	122.07			225.12	113.07	I	1290.67	645.84	1273.64	637.32	1272.66	636.83	10
3	44.05	286.18	143.59			268.17	134.59	314.17	157.59			**296.16**	148.58	A	**1177.58**	589.30	1160.56	580.78	1159.57	580.29	9
4	101.07	414.23	207.62	397.21	199.11	396.22	198.62	442.23	221.62	425.20	213.11	424.22	212.61	Q	**1106.55**	553.78	**1089.52**	545.26	**1088.54**	544.77	8
5	88.04	529.26	265.13	512.24	256.62	511.25	256.13	557.26	279.13	540.23	270.62	539.25	270.13	D	**978.49**	489.75	961.46	481.23	960.48	480.74	7
6	120.08	676.33	338.67	659.30	330.16	658.32	329.66	704.32	352.67	687.30	344.15	686.31	343.66	F	**863.46**	432.23	846.44	423.72	845.45	423.23	6
7	185.13	888.45	444.73	871.42	436.21	870.44	435.72	**916.44**	458.72	899.41	450.21	898.43	449.72	K	**716.39**	358.70	699.37	350.19	698.38	349.70	5
8	74.06	989.49	495.25	972.47	486.74	971.48	486.25	1017.49	509.25	1000.46	500.73	999.48	500.24	T	504.28	252.64	487.25	244.13	486.27	243.64	4
9	88.04	1104.52	552.76	1087.49	544.25	1086.51	543.76	1132.52	566.76	1115.49	558.25	1114.51	557.76	D	403.23	202.12	386.20	193.61	385.22	193.11	3
10	86.10	1217.60	609.31	1200.58	600.79	1199.59	600.30	1245.60	623.30	1228.57	614.79	1227.59	614.30	L	**288.20**	144.61	271.18	136.09			2
11	129.11													R	**175.12**	88.06	158.09	79.55			1

H3K122	RVTIMPKacacDIQLAR	Human HepG2 cells

MS/MS Fragmentation of **RVTIMPKDIQLAR**
Found in **Q71DI3**, Histone H3.2 OS=Homo sapiens OX=9606 GN=HIST2H3A PE=1 SV=3

Match to Query 14762: 1623.910448 from(812.962500,2+)
Title: File:20201204_C12_HepG2_ingel_3_1.mzXML Scans:18221 RT:80.7747min Charge:2+ Fragmentation:cid
Data file 20201204_C12_HepG2_ingel_3_1.mgf

Click mouse within plot area to zoom in by factor of two about that point
Or, Plot from 200 to 1700 Da Full range
Label all possible matches ○ Label matches used for scoring ◉

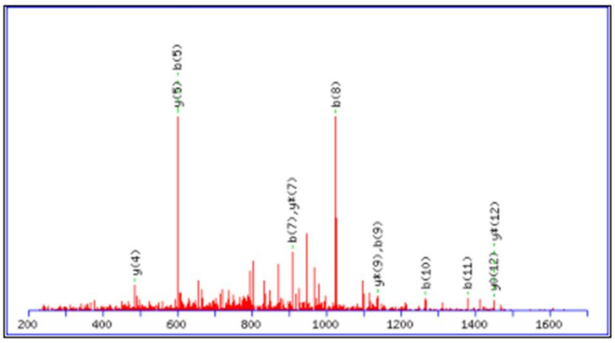

Monoisotopic mass of neutral peptide Mr(calc): 1623.9130
Variable modifications:
K7 : Acetoacetyl (K)
Ions Score: 31 **Expect:** 0.12
Matches : 13/130 fragment ions using 28 most intense peaks (help)

#	b	b^{++}	b*	b^{*++}	b^0	b^{0++}	Seq	y	y^{++}	y*	y^{*++}	y^0	y^{0++}	#
1	157.1084	79.0578	140.0818	70.5446			R							13
2	256.1768	128.5920	239.1503	120.0788			V	1468.8192	734.9132	1451.7927	726.4000	1450.8086	725.9080	12
3	357.2245	179.1159	340.1979	170.6026	339.2139	170.1106	T	1369.7508	685.3790	1352.7243	676.8658	1351.7402	676.3738	11
4	470.3085	235.6579	453.2820	227.1446	452.2980	226.6526	I	1268.7031	634.8552	1251.6766	626.3419	1250.6926	625.8499	10
5	***601.3490***	301.1782	584.3225	292.6649	583.3385	292.1729	M	1155.6191	578.3132	1138.5925	569.7999	1137.6085	569.3079	9
6	698.4018	349.7045	681.3752	341.1913	680.3912	340.6993	P	1024.5786	512.7929	1007.5520	504.2796	1006.5680	503.7876	8
7	***910.5179***	455.7626	893.4913	447.2493	892.5073	446.7573	K	927.5258	464.2665	910.4993	455.7533	909.5152	455.2613	7
8	***1025.5448***	513.2761	1008.5183	504.7628	1007.5343	504.2708	D	715.4097	358.2085	698.3832	349.6952	697.3991	349.2032	6
9	***1138.6289***	569.8181	1121.6023	561.3048	1120.6183	560.8128	I	**600.3828**	300.6950	583.3562	292.1817			5
10	***1266.6875***	633.8474	1249.6609	625.3341	1248.6769	624.8421	Q	**487.2987**	244.1530	470.2722	235.6397			4
11	***1379.7715***	690.3894	1362.7450	681.8761	1361.7610	681.3841	L	359.2401	180.1237	342.2136	171.6104			3
12	***1450.8086***	725.9080	1433.7821	717.3947	1432.7981	716.9027	A	246.1561	123.5817	229.1295	115.0684			2
13							R	175.1190	88.0631	158.0924	79.5498			1

H4K5	GKacacGGKacGLGK	Human HepG2 cells

MS/MS Fragmentation of **GKGGKGLGK**
Found in **P62805**, Histone H4 OS=Homo sapiens OX=9606 GN=HIST1H4A PE=1 SV=2

Match to Query 3021: 926.520448 from(464.267500,2+)
Title: File:20201204_C12_HepG2_ingel_3_1.mzXML Scans:8570 RT:44.5735min Charge:2+ Fragmentation:cid
Data file 20201204_C12_HepG2_ingel_3_1.mgf

Click mouse within plot area to zoom in by factor of two about that point
Or, Plot from 100 to 950 Da Full range
Label all possible matches ○ Label matches used for scoring ◉

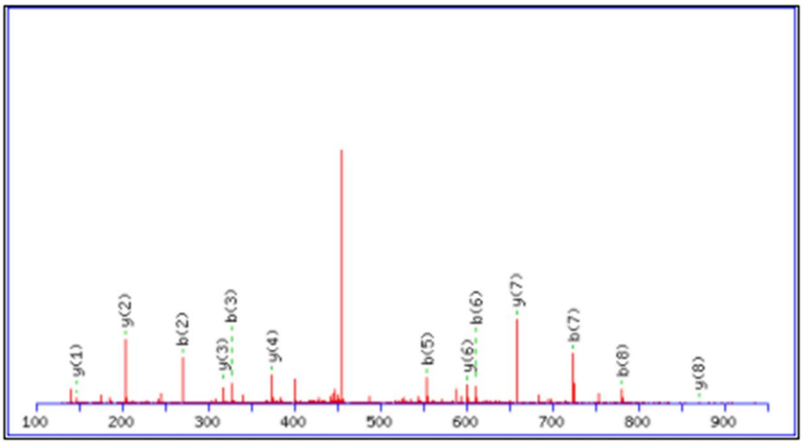

Monoisotopic mass of neutral peptide Mr(calc): 926.5185
Variable modifications:
K2 : Acetoacetyl (K)
K5 : Acetyl (K)
Ions Score: 47 **Expect:** 0.004
Matches : 13/62 fragment ions using 32 most intense peaks (help)

#	b	b^{++}	b*	b^{*++}	Seq	y	y^{++}	y*	y^{*++}	#
1	58.0287	29.5180			G					9
2	**270.1448**	135.5761	253.1183	127.0628	K	***870.5043***	435.7558	853.4778	427.2425	8
3	**327.1663**	164.0868	310.1397	155.5735	G	***658.3883***	329.6978	641.3617	321.1845	7
4	384.1878	192.5975	367.1612	184.0842	G	***601.3668***	301.1870	584.3402	292.6738	6
5	**554.2933**	277.6503	537.2667	269.1370	K	544.3453	272.6763	527.3188	264.1630	5
6	**611.3148**	306.1610	594.2882	297.6477	G	***374.2398***	187.6235	357.2132	179.1103	4
7	**724.3988**	362.7030	707.3723	354.1898	L	***317.2183***	159.1128	300.1918	150.5995	3
8	**781.4203**	391.2138	764.3937	382.7005	G	***204.1343***	102.5708	187.1077	94.0575	2
9					K	***147.1128***	74.0600	130.0863	65.5468	1

H4K8	GKprGGKacacGLGKdiGGAKprR	Human MCF-7 cells

MS/MS Fragmentation of **GKGGKGLGKGGAKR**
Found in **P62805**, Histone H4
Match to Query 11538: 1517.905448 from(759.960000,2+)

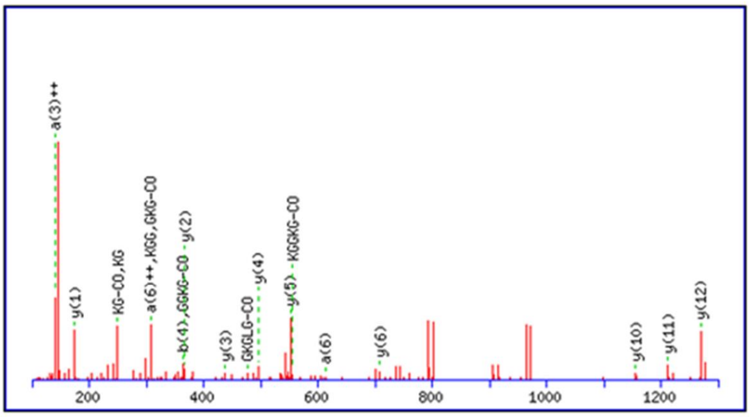

Monoisotopic mass of neutral peptide Mr(calc): 1517.91
Variable modifications:
K2 : Propionyl (K8)
K5 : Acetoacetyl (K8)
K9 : Dimethyl (K8R)
K13 : Propionyl (K8)
Ions Score: 39 **Expect:** 0.17
Matches (Bold Red): 26/262 fragment ions using 46 most intense peaks

#	Immon.	a	a^{++}	a*	a^{*++}	b	b^{++}	b*	b^{*++}	Seq.	y	y^{++}	y*	y^{*++}	#
1	30.03	30.03	15.52			58.03	29.52			G					14
2	165.15	222.17	111.59	205.14	103.07	**250.16**	125.59	233.14	117.07	K	1461.90	731.45	1444.87	722.94	13
3	30.03	279.19	**140.10**	262.16	131.59	**307.19**	154.10	290.16	145.58	G	**1269.76**	635.38	1252.73	626.87	12
4	30.03	336.21	168.61	319.19	160.10	**364.21**	182.61	347.18	174.09	G	**1212.74**	606.87	1195.71	598.36	11
5	193.14	**556.34**	278.67	539.32	270.16	584.34	292.67	567.31	284.16	K	**1155.72**	578.36	1138.69	569.85	10
6	30.03	**613.36**	**307.19**	596.34	298.67	641.36	321.18	624.33	312.67	G	935.59	468.30	918.56	459.78	9
7	86.10	726.45	363.73	709.42	355.21	754.44	377.73	737.42	369.21	L	878.57	439.79	861.54	431.27	8
8	30.03	783.47	392.24	766.44	383.73	811.46	406.24	794.44	397.72	G	765.48	383.24	748.46	374.73	7
9	129.14	939.60	470.30	922.57	461.79	967.59	484.30	950.56	475.79	K	**708.46**	354.73	691.43	346.22	6
10	30.03	996.62	498.81	979.59	490.30	1024.61	512.81	1007.59	504.30	G	**552.33**	276.67	535.31	268.16	5
11	30.03	1053.64	527.32	1036.61	518.81	1081.63	541.32	1064.61	532.81	G	**495.31**	248.16	478.29	239.65	4
12	44.05	1124.68	562.84	1107.65	554.33	1152.67	576.84	1135.64	568.33	A	**438.29**	219.65	421.26	211.14	3
13	165.15	1316.81	658.91	1299.78	650.40	1344.81	672.91	1327.78	664.39	K	**367.25**	184.13	350.23	175.62	2
14	129.11									R	**175.12**	88.06	158.09	79.55	1

H4K12	GLGKacacGGAKacR	Human HepG2 cells

MS/MS Fragmentation of **GLGKGGAKR**
Found in **P62805**, Histone H4 OS=Homo sapiens OX=9606 GN=HIST1H4A PE=1 SV=2

Match to Query 6961: 968.538248 from(485.276400,2+)
Title: File:20201205_C12_HepG2_target_2.mzXML Scans:7431 RT:48.2668min Charge:2+ Fragmentation:cid
Data file 20201205_C12_HepG2_target_2.mgf

Click mouse within plot area to zoom in by factor of two about that point
Or, Plot from 100 to 950 Da Full range
Label all possible matches ○ Label matches used for scoring ◉

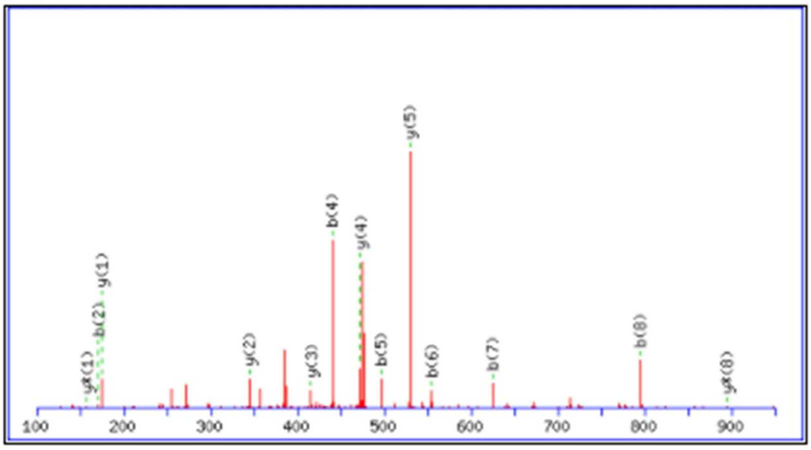

Monoisotopic mass of neutral peptide Mr(calc): 968.5403
Variable modifications:
K4 : Acetoacetyl (K)
K8 : Acetyl (K)
Ions Score: 28 **Expect:** 0.13
Matches : 13/58 fragment ions using 48 most intense peaks (help)

#	b	b++	b*	b*++	Seq.	y	y++	y*	y*++	#
1	58.0287	29.5180			G					9
2	171.1128	86.0600			L	912.5261	456.7667	895.4996	448.2534	8
3	228.1343	114.5708			G	799.4421	400.2247	782.4155	391.7114	7
4	440.2504	220.6288	423.2238	212.1155	K	742.4206	371.7139	725.3941	363.2007	6
5	497.2718	249.1395	480.2453	240.6263	G	530.3045	265.6559	513.2780	257.1426	5
6	554.2933	277.6503	537.2667	269.1370	G	473.2831	237.1452	456.2565	228.6319	4
7	625.3304	313.1688	608.3039	304.6556	A	416.2616	208.6344	399.2350	200.1212	3
8	795.4359	398.2216	778.4094	389.7083	K	345.2245	173.1159	328.1979	164.6026	2
9					R	175.1190	88.0631	158.0924	79.5498	1

H4K20	KacacVLRDNIQGITKprPAIR	Rat liver

MS/MS Fragmentation of **KVLRDNIQGITKPAIR**
Found in **P62804**, Histone H4 OS=Rattus norvegicus OX=10116 GN=Hist1h4b PE=1 SV=2
Match to Query 11928: 1961.172848 from(981.593700,2+)
Title: File:20201016_gel_6_global.mzXML Scans:11564 RT:62.4307min Charge:2+ Fragmentation:cid
Data file 20201016_gel_6_global.mgf

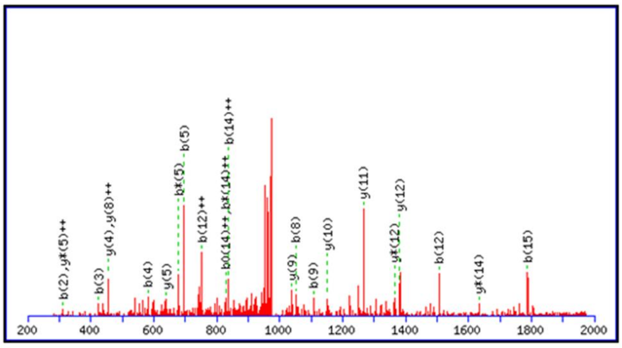

Monoisotopic mass of neutral peptide Mr(calc): 1961.1422
Variable modifications:
K1 : Acetoacetyl (K)
K12 : Propionyl (K)
Ions Score: 53 Expect: 0.00021
Matches : 23/162 fragment ions using 34 most intense peaks

#	b	b^{++}	b^{*}	b^{*++}	b^{0}	b^{0++}	Seq.	y	y^{++}	y^{*}	y^{*++}	y^{0}	y^{0++}	#
1	213.1234	107.0653	196.0968	98.5520			K							16
2	312.1918	156.5995	295.1652	148.0863			V	1750.0334	875.5203	1733.0068	867.0071	1732.0228	866.5150	15
3	425.2758	213.1416	408.2493	204.6283			L	1650.9650	825.9861	1633.9384	817.4728	1632.9544	816.9808	14
4	581.3770	291.1921	564.3504	282.6788			R	1537.8809	769.4441	1520.8544	760.9308	1519.8703	760.4388	13
5	696.4039	348.7056	679.3774	340.1923	678.3933	339.7003	D	1381.7798	691.3935	1364.7532	682.8803	1363.7692	682.3883	12
6	810.4468	405.7271	793.4203	397.2138	792.4363	396.7218	N	1266.7528	633.8801	1249.7263	625.3668	1248.7423	624.8748	11
7	923.5309	462.2691	906.5043	453.7558	905.5203	453.2638	I	1152.7099	576.8586	1135.6834	568.3453	1134.6994	567.8533	10
8	1051.5895	526.2984	1034.5629	517.7851	1033.5789	517.2931	Q	1039.6259	520.3166	1022.5993	511.8033	1021.6153	511.3113	9
9	1108.6109	554.8091	1091.5844	546.2958	1090.6004	545.8038	G	911.5673	456.2873	894.5407	447.7740	893.5567	447.2820	8
10	1221.6950	611.3511	1204.6684	602.8379	1203.6844	602.3459	I	854.5458	427.7765	837.5193	419.2633	836.5352	418.7713	7
11	1322.7427	661.8750	1305.7161	653.3617	1304.7321	652.8697	T	741.4618	371.2345	724.4352	362.7212	723.4512	362.2292	6
12	1506.8639	753.9356	1489.8373	745.4223	1488.8533	744.9303	K	640.4141	320.7107	623.3875	312.1974			5
13	1603.9166	802.4619	1586.8901	793.9487	1585.9061	793.4567	P	456.2929	228.6501	439.2663	220.1368			4
14	1674.9537	837.9805	1657.9272	829.4672	1656.9432	828.9752	A	359.2401	180.1237	342.2136	171.6104			3
15	1788.0378	894.5225	1771.0112	886.0093	1770.0272	885.5173	I	288.2030	144.6051	271.1765	136.0919			2
16							R	175.1190	88.0631	158.0924	79.5498			1

H4K31	DNIQGITKacacPAIR	Human MCF-7 cell

MS/MS Fragmentation of **DNIQGITKPAIR**
Found in **P62805**, Histone H4
Match to Query 5149: 1408.764696 from(705.389624,2+)

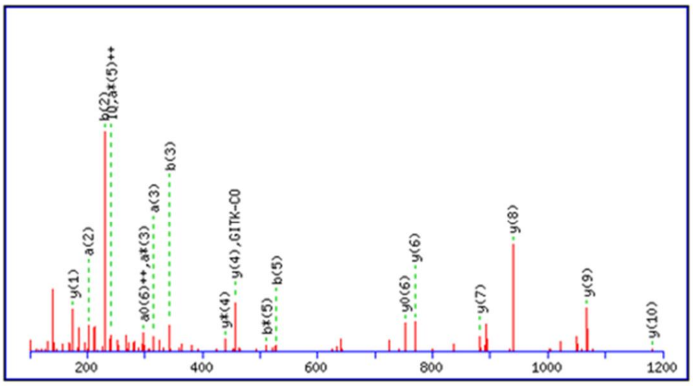

Monoisotopic mass of neutral peptide Mr(calc): 1408.77
Variable modifications:
K8 : Acetoacetyl (K)
Ions Score: 50 **Expect:** 0.012
Matches (Bold Red): 20/263 fragment ions using 27 most intense peaks

#	Immon.	a	a^{++}	a^*	a^{*++}	a^0	a^{0++}	b	b^{++}	b^*	b^{*++}	b^0	b^{0++}	Seq.	y	y^{++}	y^*	y^{*++}	y^0	y^{0++}	#
1	88.04	88.04	44.52			70.03	35.52	116.03	58.52			98.02	49.52	D							12
2	87.06	**202.08**	101.54	185.06	93.03	184.07	92.54	**230.08**	115.54	213.05	107.03	212.07	106.54	N	1294.75	647.88	1277.72	639.36	1276.74	638.87	11
3	86.10	**315.17**	158.09	**298.14**	149.57	297.16	149.08	**343.16**	172.08	326.13	163.57	325.15	163.08	I	**1180.70**	590.86	1163.68	582.34	1162.69	581.85	10
4	101.07	443.22	222.12	426.20	213.60	425.21	213.11	471.22	236.11	454.19	227.60	453.21	227.11	Q	**1067.62**	534.31	1050.59	525.80	1049.61	525.31	9
5	30.03	500.25	250.63	483.22	**242.11**	482.24	241.62	**528.24**	264.62	**511.21**	256.11	510.23	255.62	G	**939.56**	470.28	922.54	461.77	921.55	461.28	8
6	86.10	613.33	307.17	596.30	298.66	595.32	**298.16**	641.33	321.17	624.30	312.65	623.31	312.16	I	**882.54**	441.77	865.51	433.26	864.53	432.77	7
7	74.06	714.38	357.69	697.35	349.18	696.37	348.69	742.37	371.69	725.35	363.18	724.36	362.68	T	**769.46**	385.23	752.43	376.72	**751.45**	376.23	6
8	185.13	926.49	463.75	909.47	455.24	908.48	454.75	954.49	477.75	937.46	469.23	936.48	468.74	K	668.41	334.71	651.38	326.19			5
9	70.07	1023.55	512.28	1006.52	503.76	1005.54	503.27	1051.54	526.27	1034.52	517.76	1033.53	517.27	P	**456.29**	228.65	**439.27**	220.14			4
10	44.05	1094.58	547.80	1077.56	539.28	1076.57	538.79	1122.58	561.79	1105.55	553.28	1104.57	552.79	A	359.24	180.12	342.21	171.61			3
11	86.10	1207.67	604.34	1190.64	595.82	1189.66	595.33	1235.66	618.34	1218.64	609.82	1217.65	609.33	I	288.20	144.61	271.18	136.09			2
12	129.11													R	**175.12**	88.06	158.09	79.55			1

H4K44	GGVKacacRdiISGLIYEETR	Human HeLA cells

MS/MS Fragmentation of **GGVKRISGLIYEETR**

Found in **P62805**, Histone H4

Match to Query 9122: 1788.968172 from(597.330000,3+)

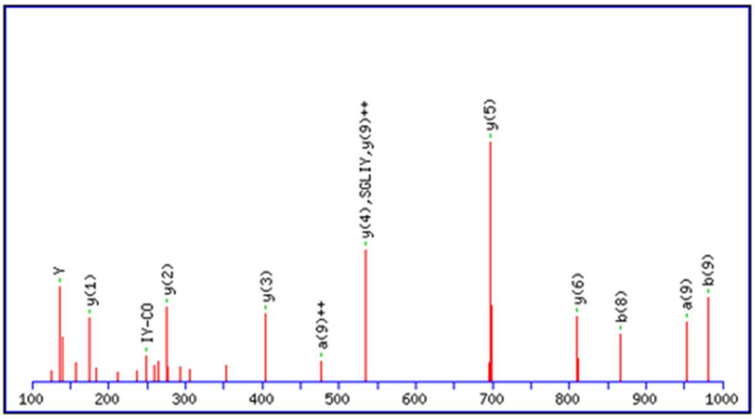

Monoisotopic mass of neutral peptide Mr(calc): 1788.97

Variable modifications:

K4 : Acetoacetyl (K)

R5 : Dimethyl0 (KR)

Ions Score: 38 **Expect:** 0.52

Matches (Bold Red): 14/320 fragment ions using 17 most intense peaks

#	Immon.	a	a^{++}	a^{*}	a^{*++}	a^{0}	a^{0++}	b	b^{++}	b^{*}	b^{*++}	b^{0}	b^{0++}	Seq.	y	y^{++}	y^{*}	y^{*++}	y^{0}	y^{0++}	#
1	30.03	30.03	15.52					58.03	29.52					G							15
2	30.03	87.06	44.03					115.05	58.03					G	1732.96	866.98	1715.93	858.47	1714.95	857.98	14
3	72.08	186.12	93.57					214.12	107.56					V	1675.94	838.47	1658.91	829.96	1657.93	829.47	13
4	185.13	398.24	199.62	381.21	191.11			426.23	213.62	409.21	205.11			K	1576.87	788.94	1559.84	780.43	1558.86	779.93	12
5	157.14	582.37	291.69	565.35	283.18			610.37	305.69	593.34	297.17			R	1364.75	682.88	1347.73	674.37	1346.74	673.87	11
6	86.10	695.46	348.23	678.43	339.72			723.45	362.23	706.42	353.72			I	1180.62	590.81	1163.59	582.30	1162.61	581.81	10
7	60.04	782.49	391.75	765.46	383.23	764.48	382.74	810.48	405.75	793.46	397.23	792.47	396.74	S	1067.54	**534.27**	1050.51	525.76	1049.53	525.27	9
8	30.03	839.51	420.26	822.48	411.75	821.50	411.25	**867.50**	434.26	850.48	425.74	849.49	425.25	G	980.50	490.76	963.48	482.24	962.49	481.75	8
9	86.10	**952.59**	**476.80**	935.57	468.29	934.58	467.80	**980.59**	490.80	963.56	482.28	962.58	481.79	L	923.48	462.25	906.46	453.73	905.47	453.24	7
10	86.10	1065.68	533.34	1048.65	524.83	1047.67	524.34	1093.67	547.34	1076.65	538.83	1075.66	538.33	I	**810.40**	405.70	793.37	397.19	792.39	396.70	6
11	**136.08**	1228.74	614.87	1211.71	606.36	1210.73	605.87	1256.74	628.87	1239.71	620.36	1238.73	619.87	Y	**697.32**	349.16	680.29	340.65	679.30	340.16	5
12	102.05	1357.78	679.40	1340.76	670.88	1339.77	670.39	1385.78	693.39	1368.75	684.88	1367.77	684.39	E	**534.25**	267.63	517.23	259.12	516.24	258.62	4
13	102.05	1486.83	743.92	1469.80	735.40	1468.82	734.91	1514.82	757.91	1497.79	749.40	1496.81	748.91	E	**405.21**	203.11	388.18	194.59	387.20	194.10	3
14	74.06	1587.87	794.44	1570.85	785.93	1569.86	785.44	1615.87	808.44	1598.84	799.92	1597.86	799.43	T	**276.17**	138.59	259.14	130.07	258.16	129.58	2
15	129.11													R	**175.12**	88.06	158.09	79.55			1

H4K59	GVLKacacVFLENVIR	Human HepG2 cells

MS/MS Fragmentation of GVLKVFLENVIR
Found in P62805, Histone H4 OS=Homo sapiens OX=9606 GN=HIST1H4A PE=1 SV=2

Match to Query 10362: 1469.861048 from(735.937800,2+)
Title: File:20201204_C12_HepG2_ingel_1_1.mzXML Scans:28113 RT:116.9038min Charge:2+ Fragmentation:cid
Data file 20201204_C12_HepG2_ingel_1_1.mgf

Click mouse within plot area to zoom in by factor of two about that point
Or, Plot from 200 to 1500 Da Full range
Label all possible matches ○ Label matches used for scoring ◉

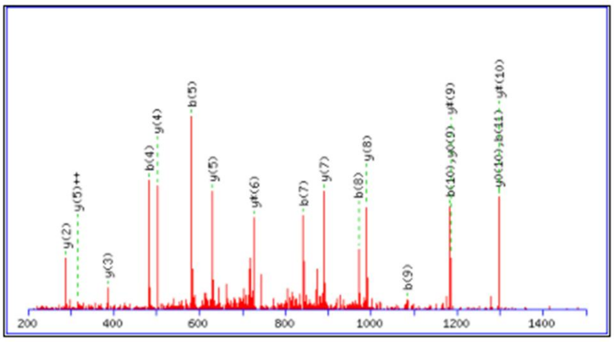

Monoisotopic mass of neutral peptide Mr(calc): 1469.8608
Variable modifications:
K4 : Acetoacetyl (K)
Ions Score: 48 **Expect:** 0.0021
Matches : 19/104 fragment ions using 28 most intense peaks (help)

#	b	b^{++}	b^{*}	b^{*++}	b^{0}	b^{0++}	Seq	y	y^{++}	y^{*}	y^{*++}	y^{0}	y^{0++}	#
1	58.0287	29.5180					G							12
2	157.0972	79.0522					V	1413.8464	707.4268	1396.8199	698.9136	1395.8358	698.4216	11
3	270.1812	135.5942					L	1314.7780	657.8926	1297.7514	649.3794	1296.7674	648.8874	10
4	***482.2973***	241.6523	465.2708	233.1390			K	1201.6939	601.3506	1184.6674	592.8373	1183.6834	592.3453	9
5	***581.3657***	291.1865	564.3392	282.6732			V	**989.5778**	495.2926	972.5513	486.7793	971.5673	486.2873	8
6	728.4341	364.7207	711.4076	356.2074			F	**890.5094**	445.7584	873.4829	437.2451	872.4989	436.7531	7
7	***841.5182***	421.2627	824.4917	412.7495			L	743.4410	372.2241	726.4145	363.7109	725.4304	363.2189	6
8	***970.5608***	485.7840	953.5342	477.2708	952.5502	476.7788	E	**630.3570**	315.6821	613.3304	307.1688	612.3464	306.6768	5
9	***1084.6037***	542.8055	1067.5772	534.2922	1066.5932	533.8002	N	**501.3144**	251.1608	484.2878	242.6475			4
10	***1183.6721***	592.3397	1166.6456	583.8264	1165.6616	583.3344	V	**387.2714**	194.1394	370.2449	185.6261			3
11	***1296.7562***	648.8817	1279.7297	640.3685	1278.7456	639.8765	I	**288.2030**	144.6051	271.1765	136.0919			2
12							R	175.1190	88.0631	158.0924	79.5498			1

H4K79	KacacTVTAMDVVYALK	Human HepG2 cells

MS/MS Fragmentation of **KTVTAMDVVYALK**
Found in **P62805**, Histone H4 OS=Homo sapiens OX=9606 GN=HIST1H4A PE=1 SV=2

Match to Query 13475: 1521.813848 from(761.914200,2+)
Title: File:20201204_C12_HepG2_ingel_3_1.mzXML Scans:19477 RT:85.5885min Charge:2+ Fragmentation:cid
Data file 20201204_C12_HepG2_ingel_3_1.mgf

Click mouse within plot area to zoom in by factor of two about that point
Or, [Plot from] [200] to [1600] Da [Full range]
Label all possible matches ○ Label matches used for scoring ◉

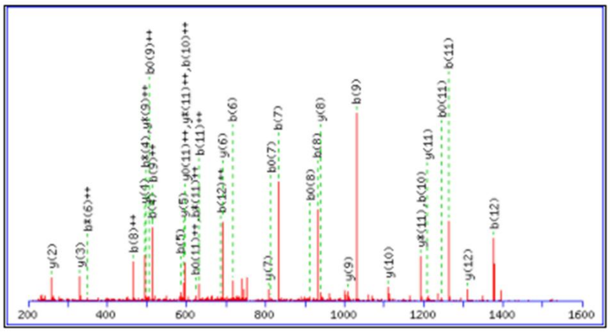

Monoisotopic mass of neutral peptide Mr(calc): 1521.8118
Variable modifications:
K1 : Acetoacetyl (K)
Ions Score: 52 **Expect:** 0.00057
Matches : 37/180 fragment ions using 90 most intense peaks (help)

#	b	b++	b*	b*++	b0	b0++	Seq	y	y++	y*	y*++	y0	y0++	#
1	213.1234	107.0653	196.0968	98.5520			K							13
2	314.1710	157.5892	297.1445	149.0759	296.1605	148.5839	T	1310.7025	655.8549	1293.6759	647.3416	1292.6919	646.8496	12
3	413.2395	207.1234	396.2129	198.6101	395.2289	198.1181	V	1209.6548	605.3310	1192.6282	596.8178	1191.6442	596.3257	11
4	514.2871	257.6472	497.2606	249.1339	496.2766	248.6419	T	1110.5864	555.7968	1093.5598	547.2835	1092.5758	546.7915	10
5	585.3243	293.1658	568.2977	284.6525	567.3137	284.1605	A	1009.5387	505.2730	992.5121	496.7597	991.5281	496.2677	9
6	716.3647	358.6860	699.3382	350.1727	698.3542	349.6807	M	938.5016	469.7544	921.4750	461.2411	920.4910	460.7491	8
7	831.3917	416.1995	814.3651	407.6862	813.3811	407.1942	D	807.4611	404.2342	790.4345	395.7209	789.4505	395.2289	7
8	930.4601	465.7337	913.4335	457.2204	912.4495	456.7284	V	692.4341	346.7207	675.4076	338.2074			6
9	1029.5285	515.2679	1012.5020	506.7546	1011.5179	506.2626	V	593.3657	297.1865	576.3392	288.6732			5
10	1192.5918	596.7996	1175.5653	588.2863	1174.5813	587.7943	Y	494.2973	247.6523	477.2708	239.1390			4
11	1263.6290	632.3181	1246.6024	623.8048	1245.6184	623.3128	A	331.2340	166.1206	314.2074	157.6074			3
12	1376.7130	688.8601	1359.6865	680.3469	1358.7025	679.8549	L	260.1969	130.6021	243.1703	122.0888			2
13							K	147.1128	74.0600	130.0863	65.5468			1
